# Primary ovarian insufficiency: update on clinical and genetic findings

**DOI:** 10.3389/fendo.2024.1464803

**Published:** 2024-09-26

**Authors:** Silvia Federici, Raffaella Rossetti, Silvia Moleri, Elisabetta V. Munari, Maria Frixou, Marco Bonomi, Luca Persani

**Affiliations:** ^1^ Department of Medical Biotechnologies and Translational Medicine, University of Milan, Milan, Italy; ^2^ Department of Endocrine and Metabolic Diseases, IRCCS Istituto Auxologico Italiano, Milan, Italy

**Keywords:** female hypogonadism, estradiol, estradiol replacement therapy, premature menopause, premature ovarian failure

## Abstract

Primary ovarian insufficiency (POI) is a disorder of insufficient ovarian follicle function before the age of 40 years with an estimated prevalence of 3.7% worldwide. Its relevance is emerging due to the increasing number of women desiring conception late or beyond the third decade of their lives. POI clinical presentation is extremely heterogeneous with a possible exordium as primary amenorrhea due to ovarian dysgenesis or with a secondary amenorrhea due to different congenital or acquired abnormalities. POI significantly impacts non only on the fertility prospect of the affected women but also on their general, psychological, sexual quality of life, and, furthermore, on their long-term bone, cardiovascular, and cognitive health. In several cases the underlying cause of POI remains unknown and, thus, these forms are still classified as idiopathic. However, we now know the age of menopause is an inheritable trait and POI has a strong genetic background. This is confirmed by the existence of several candidate genes, experimental and natural models. The most common genetic contributors to POI are the X chromosome-linked defects. Moreover, the variable expressivity of POI defect suggests it can be considered as a multifactorial or oligogenic defect. Here, we present an updated review on clinical findings and on the principal X-linked and autosomal genes involved in syndromic and non-syndromic forms of POI. We also provide current information on the management of the premature hypoestrogenic state as well as on fertility preservation in subjects at risk of POI.

## 1 Introduction

Primary ovarian insufficiency (POI) is characterized by impaired or intermittent ovarian follicle function before age 40 (1, 2), determined by diminished number of primordial follicles, accelerated follicular atresia, blocked follicle maturation or follicular dysfunction. The cut-off age of 40 years is used because it represents two standard deviations below the mean age of natural menopause. The definition and appropriate terminology of this condition have been debated for decades (3): the aforementioned definition of POI, which should replace the terminology of ‘premature ovarian failure (POF)’, has the advantage of clearly defining the ovarian origin of the condition, as is common practice in endocrinology. Moreover, the latter definition does not take into account the biological course of the condition, and is uninformative and stigmatizing for patients who may not experience the cessation of ovarian function at the time of diagnosis. POI should be intended as a wide clinical spectrum, with considerable variability of clinical presentation and its own natural history. It is a condition characterized by strong genetic susceptibility, occurring in up to 30% of cases in familial form, whose expression is modulated by various environmental factors (4, 5). It is a heterogeneous disorder, which can be acquired or congenital, although in 70-90% of cases it remains idiopathic (6), although more recent data suggest that, as a result of the effort made in identifying new genetic mechanisms, the percentage of idiopathic forms currently stands at 39% to 67% (2). Depletion or dysfunction of ovarian follicles leads to amenorrhea and subsequently to menopausal symptoms, infertility, and sexual dysfunction which adversely impact not only the physical and mental well-being of those affected but also their self-esteem and interpersonal relationships (7). Thus, the quality of life of women with POI is affected from a physical, psychological, and social point of view. The relevance of POI is emerging as the desire of women to conceive beyond the age of 30 grows, when the incidence of POI is the greatest, and life expectancy is extended, and, in turn, also the duration of hypoestrogenism. Considering the rising incidence of the condition in younger ages as well (8, 9), POI presents a growing challenge for women, as it interferes with their reproductive desires. At the same time, POI diagnosis, especially at younger ages, heightens the risk of associated morbidities and is expected to lead to early mortality, thereby having serious consequences on the health of those affected (10, 11). Therefore, gaining a deep understanding of POI is critical for its early diagnosis, development of an effective long-term management and patient counseling strategy. This would finally lead to improvements in the overall quality of life, including the physical and psychological well-being, reproductive health and primary life goals of the affected women.

## 2 Epidemiology

The Study of Women’s Health Across the Nation (SWAN) reported that approximately 1.1% of women under the age of 40 in the general population are affected by POI (12). However, a recent large-scale meta-analysis study estimated the global prevalence of POI to be 3.7% (13). The incidence of POI declines exponentially with decreasing age. Specifically, the incidence rate ratios are 1:100 for women between 35 and 40 years old, 1:1,000 for women between 25 and 30 years old, and 1:10,000 for women between 18 and 25 years old (14). Interestingly, a nationwide Israeli study in women under 21 years of age, showed that the incidence rate of POI diagnoses doubled in the period of 2009-2016 compared to the period of 2000-2008 (8). Additionally, the results of a recent Finnish study suggest an increase in the incidence rate of the condition in adolescent girls, aged 15-19, from 2007 to 2017 (9). These results place emphasis on the discrepancy between the past and recent epidemiologic data, whilst indicating a rise in the incidence of POI amongst younger women, that could also reflect a change towards a more active approach to primary amenorrhea among adolescent women. The prevalence and incidence rates also differ across ethnicities. A multi-ethnic cross-sectional study conducted by Luborsky et al. (12) demonstrated significantly higher incidence rates in Hispanic and African American women compared to Japanese and Chinese women. Additionally, two population-based cohort studies on the Swedish and Iranian populations showed a prevalence of 1.9% and 3.5%, respectively (15, 16). Remarkably, first-degree relatives of women with POI have an increased risk of having POI themselves (9, 17, 18).

## 3 Etiopathogenesis

### 3.1 Genetic causes

Although POI negatively affects fertility, several studies have indicated that this condition has a strong heritable, and therefore genetic, component. A decade ago, Stolk and colleagues identified common loci associated with the age at menopause by a genome wide association study (19). However, the exact mechanisms underlying the heritability of this condition are not yet completely understood. The fact that mothers and daughters show a tendency of inheritance of the menopausal age supports the view of the inheritable susceptibility to POI, with a demonstrated high prevalence (31%) of familial POI in patients (18). Moreover, the even higher incidence of early menopause (EM) occurring within the same family group or among first-degree relatives also indicates a variable expression of the same genetic disease predisposition (20, 21). Two recent population-based studies further assessed the familial clustering of POI. In Finland, it has been estimated an odds ratio of 4.6 (95% CI 3.3-6.5) for POI in first-degree relatives of 129 women with POI (9). Whereas in a cohort of 396 cases from Utah, first-degree relatives demonstrated an 18-fold increased risk of POI compared with controls relative risk (RR, 18.52; 95% CI, 10.12–31.07), second-degree relatives demonstrated a 4-fold increase (RR, 4.21; 95% CI, 1.15–10.79), and third-degree relatives demonstrated a 2.7-fold increase (RR, 2.65; 95% CI, 1.14–5.21) (17). Hereditary disorders can affect the functioning of the ovaries and contribute to the development of POI: among the genetic conditions that have been associated with an increased risk of POI, there are X chromosome aneuploidies, together with polymorphisms and mutations in several causative genes, that are associated with either pleiotropic genetic syndromes or isolated cases (**Tables 1**–**3**).

**Table 1:** Syndromic forms of POI.

CHROMOSOMAL ABNORMALITIES							
Syndrome			Kariotype	Molecular Mechanism	Phenotypes	Frequency	Ref.
*Turner Syndrome* (X monosomy)			(45,X/ 46,XX), mosaicisms	Short stature and other skeletal dysmorphism may associate to cardiac defects, hearing loss and hypothyroidism. Accelerated PmFs atresia with ovarian reserve reduction cause either SA or gonadal dysgenesis (with streak ovaries and elevated FSH levels since infancy)	X structural abnormalities, with partial/complete loss of one X chromosome due to deletion, translocation, inversion, or formation of an isochromosome. Possibly, chromosomal pairing fails during meiosis or dosage effect of X-linked genes escapes X inactivation	1:2500 live births; 4-5% of POI cases	(22–24)
*Triple X Syndrome* (X trisomy)			47,XXX	Low AMH with increased FSH and LH. Occasionally longer legs, delayed language development, poor motor coordination and increased risk of POI or genitourinary abnormalities	Non-disjunction errors in meiosis I or II in oogenesis	1:1000 women (often undiagnosed due to modest symptoms)	(25, 26)
*Xp and Xq deletions*				Large deletions associated with high POI incidence at a young age, but no simple relationship between phenotype and chromosomal loss. - Xp(del): Xp11 is the most common breakpoint for terminal deletions, leading to either PA (55%) or SA (45%). Deletion of the most telomeric portion (Xp22.3 - Xpter) does not result in amenorrhea. - Xq(del): Breakpoints cluster at Xq13 - Xq21 (balanced translocations) and Xq23 - Xq27 (interstitial deletions). Xq13del are associated with PA and absent secondary sexual development. Terminal deletions at Xq25 or Xq26 seem not linked to PA; more distal deletions have mild phenotypes	Disruption of gene expression balance	4%-12% of POI cases	(27–30)
*X autosome translocations*				PA or SA; Turner stigmata if translocation occurs within the critical region Xq13-q26	Haploinsufficiency/disruption of critical genes, positional effect on contiguous genes or non-specific/defective meiotic pairing		
*Gonadal Dysgenesis*			*46,XY*	External female genitalia, bilateral streak gonads, minimal breast enlargement, propensity for malignant transformation of the gonads	Several genetic causes have been identified, including mutation in *NR5A1, SOX9, SRY, GATA4, WT1*	rare	(31)
AUTOSOMAL POI SYNDROMES							
Pathology		OMIM	Gene	Gene Functions	Phenotypes	Inheritance (AD, AR)	Ref
*Pseudo-hypoparathyroidism type 1a*		#103580	*GNAS*	Ubiquitously expressed gene, but high levels in thyroid and brain. *GNAS* encodes for the α-subunit of the stimulatory GTP binding protein with key roles in several cellular responses triggered by receptor-ligand interactions	Resistance to parathyroid hormone, to thyroid-stimulating hormone, gonadotropins and growth-hormone-releasing hormone. Sometimes associated with short stature, skeletal anomalies, ectopic ossifications, obesity, mental retardation and hypogonadism with incomplete sexual maturation, delayed puberty, oligomenorrhea or SA	AD, maternally inherited. Parent-specific methylation and paternal protein expression reduction	(32–36)
*Blepharophimosis, ptosis, epicanthus inversus (BPES) type 1*		*#110100*	*FOXL2*	Expressed in the developing eyelids and in fetal/adult ovary. Role in GCs differentiation, folliculogenesis and postnatal maintenance of ovaries, estrogen production and steroidogenesis, repressing male sex determination	Bilateral eyelid dysplasia with small palpebral fissures, drooping eyelids and skin fold starting from the lower eyelid and running inward and upward, inherited with ovarian failure (PA or SA with absent or rare follicles). *FOXL2* (*605597) is occasionally described in isolated PA or SA with a prevalence of 1.0% – 3.2%	AD, AR	(37–42)
*Perrault Syndrome*		*#614129*	*CLPP*	Mitochondrial function	Mild to severe bilateral hearing loss and ovarian dysfunction, ranging from gonadal dysgenesis to SA, with streak, absent or small ovaries. In some cases, patients also present with variable neurological signs. *LARS2* (*604544) is occasionally described in non-syndromic SA	AR	(43–49)
		*#617565*	*ERAL1*				
		*#614926*	*HARS2*				
		*614917	*RMND1*				
		*609947	*PRORP*				
		*#616138*	*TWNK*				
		*#615300*	*LARS2*				
		*#233400*	*HSD17B4*	Cellular metabolism			
		*606982	*GGPS1*				
		*601498	*PEX6*				
		*605810	*MRPS7*	Mitochondrial large and small ribosomal subunit			
		*611972	*MRPL50*				
*Progressive external ophthalmoplegia* (PEO) *A1*		#157640	*POLG*	Encodes for the catalytic subunit of the mitochondrial DNA polymerase	Progressive weakness/paralysis of the eye muscle, myopathy, sensory axonal neuropathy, ataxia, parkinsonism and delayed sexual maturation, associated in women to PA or SA (with diminished follicle reserve). Multiple mtDNA deletions triggers mitochondrial depletion *POLG* (*174763) is occasionally described in isolated SA	AD, AR in isolated SA	(50–52)
*Mitochondrial DNA depletion* *syndrome 11*		#615084	*MGME1*	Mitochondrial genome integrity maintenance	PEO, respiratory insufficiency, emaciation, ptosis, gastrointestinal and skeletal abnormalities, dysphonia	AR	(53)
*Leuko-encephalolophaty*	*Ovarioleukodystrophy*	#603896	*EIF2B4*	Protein synthesis under cellular stress conditions; likely responsible of increased apoptosis of ovarian follicles	Unusual association of chronic and progressive neurological deterioration (brain white matter degeneration) and ovarian dysfunctions. *EIF2B2* (*606454) is occasionally described in isolated PA or SA	AR	(54)
			*EIF2B5*				
			*EIF2B2*				
	*LKENP*	#615889	*AARS2*	Mitochondrial alanyl-tRNA synthetase	Progressive loss of motor and cognitive skills with ovarian failure	AR	(55, 56)
Woodhouse-Sakati syndrome		#241080	*DCAF17*	Nuclear transmembrane protein that associates with ubiquitin ligase complexes	Hypogonadism, alopecia, and neurological defects	AR	(57)
*Prolyl endopeptidase-like deficiency*		*609557	*PREPL*	Regulation of exocytosis of synaptic vesicles	Neonatal hypotonia, feeding difficulties, ptosis, neuromuscular and cognitive symptoms, growth hormone deficiency and hypergonadotropic hypogonadism	AR	(58)
*Cockayne Syndrome B*		#133540	*ERCC6* (CSB-PGBD3)	Response to DNA damage	POI (principally PA) and neurological abnormalities	AD	(59)
*XRCC4-related Disorder*		#616541	*XRCC4*	Double strand breaks (DSBs) repair	Short stature, microcephaly, developmental delay, neurological defects and gonadal failure (associated with SA)	AR	(60)
*Nijmegen breakage syndrome*		#251260	*NBN*	Chromosomal stability	Facial dysmorphism, predisposition to cancer and POI. (PA or SA, with streak gonad, small ovaries). *NBN* (*602667) is occasionally described in isolated POI	AR	(61, 62)
*Ataxia telangiectasia*		#208900	*ATM*	Check-point, cellular response after DNA damages. Ovarian age control	Cerebellar ataxia, telangiectasis, immune defects, and predisposition to malignancy. Associated to PA. ATM (*607585) is occasionally described in isolated POI	AR	(63)
*Retinal Dystrophy*		#617175	*RCBTB1*	Ubiquitination processes	Retinal Dystrophy, with or without extraocular anomalies, such as POI	AR	(64)
		*616618	*ACBD5*	Peroxisomal membrane protein			(65)
*Premature aging (Progeria)*	*Bloom syndrome*	#210900	*BLM*	Helicases involved in homologous recombination repair	Acceleration of aging process and highly variable somatic abnormalities, together with hypogonadism and SA, associated with increased follicular atresia. Ovarian aging may be influenced by abnormal epigenetic modifications (changes in methylation levels, histone modifications or non-coding RNA expression) in germ cells and early embryos may influence	AR	(66)
	*Werner syndrome*	#277700	*WRN*			AR	(67)
	*Hutchinson-Gilford*	#176670	*LMNA*	Key component of the nuclear lamina		AD	(68)
*Rothmund-Thomson syndrome type 2*		*#268400*	*RECQL4*	DNA helicase	Poikiloderma, telangiectasia, photosensitivity, skin atrophy, congenital bone defects and increased risk of osteosarcoma/skin cancer together SA and gonadotropin resistance	AR	(69)
*Acromesomelic Dysplasya* *(Demirhan syndrome)*		#609441	*BMPR1B*	Gonadal and skeletal development	Chondrodysplasia characterized by short stature and hand/foot malformations, associated with PA, hypergonadotrophic hypogonadism and genital anomalies. *BMPR1B* (*603248) is occasionally described in isolated SA	AR, AD or AR in isolated POI	(70)
*Proximal symphalangism* *(SYM1)*		*#185800*	*NOG*	BMP4 and BMP7 ligand with roles in cartilage and ovarian development	Skeletal abnormalities (fusion of interphalangeal joints and carpal/tarsal bones) sometimes associated with hearing loss and SA	AD	(71)
*GAPO syndrome*		*#230740*	*ANTXR1*	Actin assembly, cell adhesion and matrix build-up	Growth retardation, alopecia, failure of tooth eruption and progressive optic atrophy, sometimes associated with SA and follicle depletion	AR	(72, 73)
*Fanconi Anemia*		#227650	*FANCA*	DNA repair after crosslinking damage (maintenance of genome stability)	Developmental defects, early-onset bone marrow failure and high predisposition to cancer. Impairment in follicular development due to defective oocyte meiosis and abnormal germ cell development with PA or SA. *FANCA* (*607139), *FANCL* (*608111), *FANCM* (* 609644), *FANCU* (*600375), *FANCD1* (*600185) are occasionally described in isolated POI (principally SA)	AR, AD or AR isolated POI	(74, 75)
		#227645	*FANCC*				
		#614083	*FANCL*				
		#605724	*FANCD1/BRCA2*				
		#617247	*FANCU/XRCC2*				
		#614082	*FANCG/XRCC9*				
Adrenal Hyperplasia		#201710	*STAR*	Steroid hormone biosynthesis	Adrenogenital syndrome, insufficient cortisol production, atypical genitalia, altered growth	AR	(76)
		#202110	*CYP17A1*				(77)
*Aromatase deficiency*		#613546	*CYP19A1*	Androgen aromatization to estrogen	Pseudohermaphroditism in female infants, delayed bone maturation during childhood and adolescence, puberty with primary amenorrhea, failure of breast development, virilization, and hypergonadotropic hypogonadism	AR	(78)
*Congenital Disorders of Glycosylation*		#212065	*PMM2*	Glycans or oligosaccharides synthesis and processing	Encephalopathy, psychomotor and growth retardation, cardiomyopathy, liver dysfunction, hypothyroidism associated with PA. *PMM2* (*601785) occasionally described in isolated PA or SA	AR	(79)
*Galactosemia*		#230400	*GALT*	Galactose metabolic pathway	Intellectual disability; developmental delay, PA with steak ovaries or few immature follicles	AR	(80)

AD, Autosomal Dominant; AR, Autosomal Recessive; PA, Primary Amenorrhea; SA, Secondary Amenorrhea; POI, Primary Ovarian Insufficiency. The symbol # before OMIM entry number indicates that it is a descriptive entry, usually of a phenotype, and does not represent a unique locus. The symbol * before an entry number indicates a gene.

**Table 2:** Classic candidate genes for POI.

Gene	OMIM	Ovarian Phenotype (PA, SA, OD); other phenotypic anomalies	Inheritance (AD, AR, XLD)	Prevalence	Ref.
*AMH*	*600957	SA	AD	0.2% - 2.0%	(81, 82)
*AMHR2*	*600956	SA	AD	0.8%– 2.4%	(81, 82)
*ANKRD31*	*618423	SA	AD	0.3%	(83)
*BMP15*	*300247	PA, SA	XLD (dominant or recessive)	0.4% – 12%	(81, 84)
*BMPR1A*	*601299	SA	AD	unknown	(85)
*BMPR2*	*600799	PA, SA	AD	1.4%	(81, 86)
*DMC1*	*602721	SA, DOR	AR	2.4%	(87, 88)
*ESR1*	*133430	SA	AD	unknown	(89, 90)
*FIGLA*	*608697	PA, OD	AR	0.3-2.5%	(91–96)
*FMR1 premutation* (55 to 200 CGG repeats)	*309550	Intellectual and developmental disabilities associated with ovarian dysfunction; Fragile X-Associated Primary Ovarian Insufficiency (FXPOI)	XLD	2% in sporadic cases 14% in familial cases	(97)
*FOXO3A*	*602681	PA, SA	AD	2.2%	(98)
*FSHR*	*136435	OD, PA, SA with pubertal disorder, oligomenorrea	AR, AD	0.1% – 42.3%	(81, 82)
*GDF9*	*601918	PA, SA, delayed puberty	AR, AD	0.2% - 4.7%	(81, 99)
*HFM1*	*615684	PA, SA	AR, AD	0.8-4.2%	(100–105)
*INHA*	*147380	OD, PA, SA	AR, AD	0% – 11%	(82)
*LHCGR*	*152790	Delayed or absent menarche, SA	AR, AD	unknown	(106, 107)
*LHX8*	*604425	Primary infertility, SA	AD	0.7% - 1%	(53, 108, 109)
*MCM8*	*608187	PA, SA	AR, AD	1.25-2%	(104, 110–112)
*MCM9*	*610098	OD, PA, SA	AR, AD	1.6-8%	(53, 111, 113–116)
*MEIOB*	*617670	SA	AR	unknown	(117, 118)
*MEIOSIN*	n.d.	SA	AD	rare	(119)
*MND1*	* 611422	OD, PA, SA	AR, AD	unknown	(120)
*MRPS22*	*605810	SA, delayed puberty	AR	unknown	(121)
*MSH4*	*602105	OD, PA, SA, DOR	AR	1.2%	(81, 121, 122)
*MSH5*	*603382	SA	AR	0.6%	(81, 123)
*NANOS3*	*608229	SA	AR, AD	0.5-2.5%	(124, 125)
*NOBOX*	*610934	PA, SA (with or without delayed puberty)	AD, AR	1.2% - 9%	(81, 126, 127)
*NOTCH2*	*600275	PA, SA	AD	0.04%	(99, 128)
*NR5A1*	*184757	PA, SA, delayed puberty	AR, AD	0.3% – 2.3%	(10, 81)
*PGRMC1*	*300435	SA	XLD	2.0%	(53, 81)
*REC8*	*608193	SA	AR, AD	1.25%	(114, 129)
*SMC1B*	*608685	PA, SA	AD	2%	(53, 129)
*SOHLH1*	*610224	PA, SA	AD, AR	0.2% - 2.2%	(81, 130, 131)
*SOHLH2*	*616066	SA	AD	1.9%	(53, 132)
*STAG3*	*608489	OD, PA	AR	unknown	(53, 105, 133–136)
*SYCE1*	*611486	SA	AR, AD	<2%	(101, 113, 137–139)
*TP63*	*603273	OD, PA, SA	AD	3.75%	(140–143)

AD, Autosomal Dominant; AR, Autosomal Recessive; DOR, Decreased Ovarian Reserve; OD, Ovarian Dysgenesis; PA, Primary Amenorrhea; SA, Secondary Amenorrhea; POI, Primary Ovarian Insufficiency; XLD, X-Linked Dominant. The symbol * before an entry number indicates a gene.

**Table 3:** Proposed candidate genes for POI with still uncertain pathogenic roles.

Gene	OMIM	Ovarian Phenotype (PA, SA, OD); other phenotypic anomalies	Inheritance (AD, AR, XLD)	Prevalence	Ref.
*AR*	*313700	PA, SA	XLD	0.03%	(144)
*BRCA1*	*113705	SA	AD	1.8%	(145)
*EXO1*	*606063	PA	AD	2%	(146)
*FANCM*	*609644	SA	AR	rare	(53)
*GATA4*	*600576	PA	AR	0.1%	(53, 113)
*GJA4*	*121012	SA	AD	unknown	(53, 147)
*INSL3*	*146738	PA	AR	0.02%	(53)
*POLR3H*	*619801	PA, delayed puberty	AR	1.5%	(53, 148)
*PRDM9*	* 609760	SA	AD	0.4%	(53, 83)
*PSMC3IP*	*608665	OD, PA, SA	AR	unknown	(120, 149, 150)
*RAD51*	*179617	PA	AD	2%	(146)
*SPATA22*	*617673	SA	AR	0.1%	(151)
*STRA8*	*609987	PA	AR	rare	(119)
*WT1*	*607102	PA, SA	AD, AR	0.5%	(82)

AD, Autosomal Dominant; AR, Autosomal Recessive; OD, Ovarian Dysgenesis; PA, Primary Amenorrhea; SA, Secondary Amenorrhea; XLD, X-Linked Dominant. The symbol * before an entry number indicates a gene.

#### 3.1.1 Overview of genetic candidates and stages of folliculogenesis

**Figure 1:**
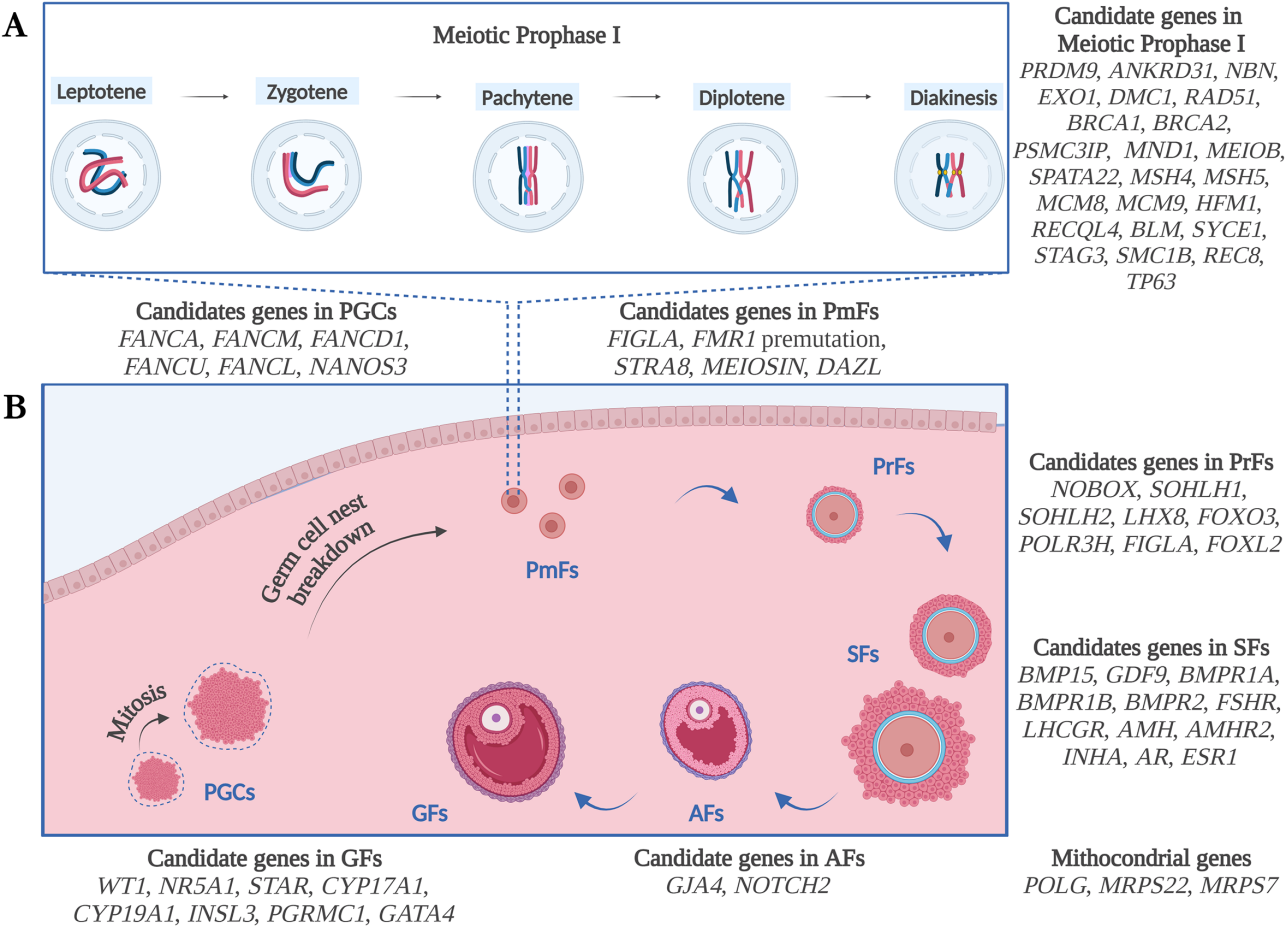
Schematic illustration of the genetic candidates for POI, according to the biological process of Meiotic Prophase I **(A)** and Folliculogenesis **(B)** and based on literature findings. PGCs, Primordial Germ Cells; PmFs, Primordial Follicles; PrFs, Primary Follicles; SFs, Secondary Follicles; Afs, Antral Follicles; GFs, Graafian Follicles. Figure created with Biorender.com.

In this section, the main genetic factors involved in non-syndromic forms of POI will be described, according to the biological process in which each gene participates, based on literature findings. See also **Figure 1** and **Tables 1**–**3** for details.

#### 3.1.1.1 Primordial germ cells and oogonia formation

Human primordial germ cells (PGCs) originate from the extraembryonic mesoderm 3 weeks after fertilization and migrate to the developing gonadal ridges, the bipotential gonads that will differentiate into either ovaries or testis. Here, female PGCs differentiate into oogonia and start proliferating in clusters of germ cells called syncytia or germ cell nests, which are connected and synchronized by intercellular connections. At this stage, an active phase of mitosis ensues to establish adequate ovarian reserve. During this phase of rapid proliferation, PGCs uphold genomic stability by crucially depending on precise DNA replication and repair mechanisms, which are tightly regulated through cell cycle blockades. The maximum number of oogonia resulting from mitotic divisions occurs approximately during the 20^th^ week of development, with the ovaries containing approximately 6-7 millions of germ cells. A recent study in knockout mice (KO) indicated that defects of Fance may act during rapid mitotic periods in PGCs, leading to impaired cell proliferation and genomic instability. Through mechanisms not completely understood, Fance^−/−^ mice showed reduced numbers of PGCs, decreased ovarian reserve, and infertility (152). In humans, previous studies have already shown that patients harboring biallelic pathogenic variants in genes such as *FANCA, FANCM, FANCD1*, *FANCU*, as well as those with monoallelic pathogenic variants in *FANCA, FANCD1, FANCL*, presented with gonadal dysfunction and infertility with or without other phenotypic features of Fanconi Anemia (74). During these early stages, mitotic oogonia expresses pluripotency-associated and germ cell-specific genes. Different groups identified *NANOS3* genetic variants in patients with POI (124, 125). The *NANOS3* gene is required for germ cell development coding an RNA-binding protein which functions by repressing apoptosis in germ cells. *ATG7* and *LIN28A* are other essential genes involved in female gametogenesis considered candidates for POI, however, no significant variations were found in POI cohorts yet (153). *ATG7* is an autophagy induction gene. Autophagy protects germ cells from over-loss in newborn ovaries. Loss of Atg7 leads to subfertility with a dramatic decrease of the ovarian follicles pool in female mice (154). *LIN28A* together with *LIN28B* encode highly conserved RNA-binding proteins regulating microRNA biogenesis by promoting germ cell proliferation and inhibiting differentiation (155); the disruption of Lin28a affects germ cell development in mice (156).

##### 3.1.1.2 Primordial to primary follicles transition

Around the 11th week of gestation, the upregulation of FIGLA drives the formation of primordial follicles. Female mice Figla^-/-^ exhibit impaired primordial follicle formation and infertility (157). Several deleterious variants of the *FIGLA* gene have been found in recent years by our group (91) and others (92–94). Each primordial follicle consists of an oocyte surrounded by a single layer of flattened somatic pre-Granulosa Cells (GCs) enclosed by a basal membrane which enters meiosis. The mechanism beyond the meiotic initiation is still poorly known. During human fetal ovarian development, increasing *FMR1* expression marks the developmental transition from primordial germ cells expressing *LIN28* to meiotic germ cells (158). The presence of the premutated *FMR1* allele is frequently associated with ovarian dysfunction. *FMR1* premutation is one of the most frequent alterations involved in the pathogenesis of SA and POI. Meiosin and Stra8 germ cell factors interact to trigger meiotic entry via the retinoic acid-dependent pathway, acting as transcription factors (159). Both *STRA8* and *MEIOSIN* variants have been found to be associated with POI (160). Once meiosis proceeds, several genes are upregulated (161). Among these, Dazl protein mediates transcription of synaptonemal complex proteins (162). SNPs and rare genetic variants of this gene could associate with an earlier onset of menopause by impairing germ cells number (163, 164).

##### 3.1.1.3 Meiotic phases

Key processes of prophase I are homologous chromosome pairing, synapsis formation, and repair of DNA Double Strand Breaks (DSBs) to enable recombination and crossover. The genetic disruption of either one of the meiotic genes leads to impaired meiosis progression and usually results in oocyte loss as shown in animal models thus making them candidates for human POI.

###### 3.1.1.3.1 DNA double strand breaks formation

The precise localization and formation of DSBs are essential for the accurate recognition and pairing of homologous chromosomes. PRDM9 is a meiosis-specific histone H3 methyltransferase which catalyzes H3K4 trimethylation, binds chromosome axis by interacting with CXXC1, HORMAD1, MEI4, REC114, ANKRD31 and IHO1, and together with Hells opens chromatin at hotspots, thus providing the access for the DSBs machinery. Subsequently, the endonuclease SPO11, along with MEI1 and TOPOVIB-Like complexes, is recruited at PRDM9-binding sites where it generates chromosome breaks. Except for Cxxc1 and Hormad1, the conditional KOs of the above-mentioned factors demonstrate female mice infertility with premature oocyte loss caused by impaired DSBs formation (165). A few pathogenic variants in *PRDM9* and *ANKRD31* (83) have been identified in patients with POI so far. Up to date, no causative variants of POI have been identified in the other meiotic DSBs genes which are rather associated with male infertility (166) or other female fertility issues different than POI, such as preimplantation embryonic arrest and recurrent implantation failure and female infertility (167, 168).

###### 3.1.1.3.2 DNA double strand breaks processing

Following the formation of DSBs, DNA ends undergo a maturation process which requires the tight cooperation of the MRE11-RAD50-NBS1 (MRN) complex. The relevance of the MRN complex in maintaining the primordial follicle pool has been demonstrated in animal models (169–171). In humans, biallelic nonsense mutations in the *NBS1/NBN* gene have been identified in compound heterozygosity in two siblings presenting the unique clinical symptom of fertility defects and POI (172). Then, the MRN complex recruits EXO1, an exonuclease which contributes to DSBs resection and formation of recombinant DNA structures and participates in mismatch repair. Inactivation of Exo1 in mice causes dynamic loss of chromosome chiasmata during the first meiotic prophase, leading to POI (173). Recently, WES performed in 50 sporadic patients with POI with PA identified in *EXO1* gene one heterozygous missense variant impairing homologous recombination (HR) (146).

###### 3.1.1.3.3 Synapsis generation

The 5’ to 3’ exonuclease activity of Exo1 generates long 3′ single-stranded tails, which serve as substrates for assembly of DMC1 and RAD51 recombinases. Both are necessary for strand invasion during synapsis formation (165). KO mice for Dmc1 show meiotic arrest at prophase I with ovaries devoid of follicles. Moreover, *DMC1* genetic variants have been described in POI (87, 174). Recently, a report described a family with a homozygous frameshift mutation as causative for both non-obstructive azoospermia and diminished ovarian reserve, suggesting that *DMC1* could be dispensable in human oogenesis (88). Contrarily, the lack of Rad51 in mice results in embryo lethality. WES in 50 sporadic patients with POI with PA identified a missense variation in *RAD51* impairing the nuclear localization of this factor (146).

The nucleation and stabilization of RAD51 at DSBs are mediated by direct interaction with BRCA2 and BRCA1-BARD1 complexes (175). *BRCA1*/2 act as tumor suppressor genes known to predispose the heterozygous carriers of deleterious mutations to breast, ovarian and other types of cancers (176) Conditional Brca2-deficient mice exhibit infertility due to defective follicular development and oocyte degeneration, whilst *BRCA2* transcript may be found downregulated in human POI oocytes (177). A few genetic studies contributed to the identification of pathogenic biallelic variants of *BRCA2* in patients with POI in absence of cancer or Fanconi Anemia trait (75, 178), thus supporting *BRCA2* haploinsufficiency as a possible mechanism leading to isolated POI (179). A tendency to accelerated decline of ovarian reserve, oocyte aging, and POI was also observed in patients carrying *BRCA1* germline variants as well as in Brca1 mouse model (180).

Other regulating factors of RAD51-DMC1 are HOP2/PSMC3IP and MND1. They function as heterodimer which enhances strand exchange on homologous DNA or containing a single mismatch (181). Aberrant synapses and DSBs repair result from the loss of this complex (182). Both the ovarian phenotype displayed by KO mice and the identification of variants in patients corroborates their key role in oogenesis and POI pathogenesis (120, 149, 150, 183). Meiob and Spata22 form a complex recruited to induce DSBs. Inactivation of Meiob and Spata22 induces meiotic arrest and leads to infertility in mice (184). Variants of *MEIOB* and *SPATA22* associated with POI are rare, with only 3 cases reported so far for *MEIOB* (117, 118) and only one for *SPATA22* (151).

###### 3.1.1.3.4 Homologous recombination

The heterodimeric complex MSH4-MSH5 stabilizes the interaction between parental chromosomes during DSBs repair (185). Disruption of either Msh4 or Msh5 genes in female mice results in infertility due to impaired and aberrant chromosome pairing, followed by apoptosis (186, 187). In the last years, WES in several POI pedigrees reported the contribution of *MSH4* and *MSH5* variants in the pathogenesis of POI. The recent identification of a digenic heterozygous variant in *MSH4/MSH5* suggested that a dysfunctional interaction or cumulative haploinsufficiency of both heterodimer subunits, may disrupt HR during meiosis, finally causing POI (81).

MCM8 and MCM9 proteins form a hexameric ATPase/helicase complex which mediates HR repair (188). HR results impaired in both Mcm8 and Mcm9 KO mice, also presenting genome instability and being predisposed to develop tumors, suggesting a role in cancer development as tumor suppressor genes (189). Evidence for an association between variants in either *MCM8* or *MCM9* comes forth in genetic studies in POI families and worldwide cohorts of patients (110). Importantly, oncologic screenings are recommended in mutations’ carriers for prevention and early diagnosis (190).

Another helicase gene involved in the realization of crossing over is *HFM1*. In line with the observed phenotype of the Hfm1-/- mice, variations in the human gene can be causative for sporadic POI (100–105). Instead, variants in helicase genes, *RECQL4* and *BLM*, which also participate in HR repair, have been identified in both Bloom and Rothmund-Thomson syndrome.

###### 3.1.1.3.5 Synaptonemal complex and cohesins

The synaptonemal complex (SC) is a protein complex that forms a zip-like structure between homologous chromosomes during meiotic prophase I. Its primary function is to uphold the pairing of homologous chromatids, thus ensuring crossing over. The central structure of SC consists of transverse filaments, constituted by Sycp1, and central elements, including among others Syce1. Whereas SYCP2 and SYCP3 form the parallel lateral elements of SC. In patients with POI, except for variants identified in *SYCE1* (101, 113, 137–139, 191, 192), no causative variations have been identified so far, although infertility has been described in animal models. Cohesins are proteins that directly associate with SC and ensure cohesion between sister chromatids. Some cases of POI have been associated with defective chromosomal cohesion due to variants in components of the ring-shaped protein structure made of cohesins. Cohesins core subunits may be meiosis-specific, such as STAG3, RAD21L, and SMC1B, or generic, such as SMC3 and REC8 (193). Pathogenic homozygous or compound heterozygous variants in *STAG3* gene have been found in several POI pedigrees. Up to date, 22 variants in *STAG3* have been reported in association with ovarian dysgenesis and PA, mostly with a predicted loss of function effect (53, 104, 105, 133–136). Variants in other cohesin genes have been firstly found for *SMC1B* and *REC8*, in heterozygosity, by a targeted NGS in 100 sporadic patients with POI (129). Accordingly, female mice KO for these cohesins are sterile with premature oocyte exhaustion and higher risk of developing ovarian tumors (Stag3^-/-^) (194) and early meiotic arrest (Smc1b^-/-^ and Rec8^-/-^) (195, 196).

###### 3.1.1.3.6 Dissolution of joint DNA intermediates

The dissolution of the SC takes place in the diplotene stage of prophase I, when recombined homologous chromosomes start separating except for the sites of crossovers. The joint DNA intermediates dissolution is executed by BLM helicase in complex with topoisomerase III alpha and other subunits. Genetic defects in this complex cause Bloom syndrome. Oocytes are then maintained at the diplotene stage for a prolonged period by high concentrations of cAMP inside the oocyte. During meiotic arrest, p63, a member of p53 transcription factor family, acts in maintaining the female germ line integrity. The alpha isoform is particularly expressed in oocytes of primordial and primary follicles in response to DNA damage and mediate apoptosis (197). The p63+/ΔTID transgenic female mice, in which the transactivation inhibitory domain of the protein is deleted, show rapid oocyte depletion through apoptosis after birth (198). Several variants located in the C-terminal region of the human *TP63* gene have been found. Recently, our group contributed to identify an intragenic duplication paternally inherited in two sisters diagnosed with ovarian dysgenesis and PA (140). A few nonsense and missense variants were then reported in literature in isolated or syndromic POI families (141, 142, 199). More recently, heterozygous pathogenic single nucleotide variants and intragenic copy number variations of *TP63* have also been described in sporadic patients with POI (143). All the identified variants are supposed to enhance oocyte apoptosis leading to premature depletion of the ovarian reserve, but further researchers may better elucidate the mechanism and pathways involved. Thereafter, through a still undeciphered mechanism, primordial follicles undergo growth and maturation, thus entering the growing pool of primary follicles (200).

##### 3.1.1.4 Follicular growth and maturation

The primordial follicle cohort may have different fates: some remain quiescent and constitute the ovarian reserve, while the majority undergo atresia, either directly or after an initial recruitment and growth, but a smaller portion is activated and then develops until ovulation. In physiological conditions, during transition from primordial to primary follicles, the oocyte resumes meiosis and gradually increases in size, while the surrounding pre-GCs enters a proliferative and differentiated state (201). However, in some pathological situations and after exposure to chemotherapy or environmental chemicals, primordial follicle depletion may accelerate thus leading to POI (202).

##### 3.1.1.5 Primary follicles development

In mammals, early follicular progression is carried on through PI3K/AKT/mTOR activation and PTEN inhibition: multiple players in these pathways have been identified although complete comprehension of these processes is still very limited, particularly in human (203, 204). In the oocyte, key events that trigger the development of primary follicles seem to be the activation of specific transcription factors, such as *NOBOX* and *SOHLH1/SOHLH2* together with *LHX8*. KO mice of these genes present with hypergonadotropic hypogonadism: in female Nobox^-/-^ fibrous tissues replace follicles (205), while in female Sohlh1-/- and Lhx8^-/-^ infertility is respectively caused by gonadal dysgenesis (206), and early oocytes loss (207). Studies in mouse indicate that NOBOX, SOHLH1/SOHLH2 and LHX8 are co-expressed and cross-regulate each other, either directly or indirectly, thus controlling oocyte development during early follicular progression and therefore their mis-regulation leads to infertility (95). Differently to NOBOX and SOHLH1/SOHLH2, LHX8 and FOXO3 maintain primordial follicles quiescent and inhibit follicular development (208). FOXO3 is a substrate of AKT. In mice, Foxo3 deficiency prematurely activates dormant follicles in the pubertal ovary, while its constitutive expression delays oocytes and follicles development (209). *NOBOX* gene is considered one of the major genetic causes of POI (126), whose variations have a high incidence in woman from sub-Saharan Africa (210), while *SOHLH1, SOHLH2, LHX8* and *FOXO3* variants are rather uncommon (98, 105, 108, 130, 132, 211). Recent WES data in a cohort of women with infertility and oocyte maturation arrest, however, report the identification of 5 novel heterozygous loss of function *LHX8* variants that produce truncated proteins (109). In turn, Foxo3a expression results diminished in mice with a homozygous point mutation in the Polr3h gene and characterized by delayed pubertal development. Remarkably, a pathogenic mutation in the *POLR3H* gene is described in two unrelated POI families, thus highlighting a new player in ovarian function and a new candidate gene (148). The continuous oocyte expression of *FIGLA* regulates the zona pellucida (ZP) genes, necessary for the production and assembly of an extracellular coat of glycoproteins which surrounds and separates the oocyte from the adjacent GCs, but allows the exchange of second messengers or small molecules through gap junctions (161, 212). At this stage, the ovarian follicle is defined a functional syncytium, that allows bidirectional communication between oocytes and GCs, and defects of ZP genes may results in female infertility in mice and humans (213). To support oocyte maturations, GCs first become cubically shaped and tight junctions appear. Then, GCs further enlarge and become stratified into multiple columnar cells, that progressively differentiate into internal cumulus cells (CCs) and external mural granulosa cells (MGCs), characterized by different metabolomes (214). FOXL2, a pleiotropic transcription factor with key roles throughout ovarian development, is critical in promoting differentiation and maintenance of GCs identity (215). Conditional loss of Foxl2 in mouse adult ovaries causes GCs reprogramming into testicular cells (216), and human *FOXL2* variants have been associated with POI (81, 217). However, follicles result histologically mostly abnormal and atretic in POI, characterized by a partial or complete absence of GCs and, therefore, an important challenge today is to identify new players in GCs development.

##### 3.1.1.6 Secondary follicles growth

Primary to small antral follicle transition depends on the oocyte production and secretion of two members of the transforming growth factor beta (TGFβ) family, GDF9 and BMP15. Evidence in natural and experimental animal models provide insight into their roles and demonstrate their oocyte-specific co-expression as cumulin (218). In particular, it has been reported that BMP15 is more relevant in mono-ovulating species (such as sheep and human) than in the poly-ovulating ones (mice) (5). In humans, mutations in *BMP15* have been first found in association with hypergonadotropic ovarian failure characterized by ovarian dysgenesis (219) and from then on other variants have been identified worldwide (220–226). *BMP15* maps on the Xp, in a locus critical for ovarian reserve determination, where several TS traits are located, including ovarian failure (227, 228). BMP15 as synergic heterodimer with GDF9 interacts with a tetrameric receptor complex on GCs formed by the kinase receptor BMPR2 and the ALK3 and ALK6 co-receptors, respectively BMPR1A and BMPR1B. *BMPR2* variants are described in potential functional association with POI (106). A variant in *BMPR1A* was found to alter downstream signaling, possibly causing POI (85), whereas mutations in *BMPR1B* have been associated with some cases of non-syndromic POI, besides those with Demirhan syndrome (99). Upon binding receptors, cumulin triggers SMAD proteins phosphorylation cascade and promotes the transcription of GCs proliferation genes (229). One of the known downstream targets of cumulin is FSHR, which expression is essential for follicle growth and later estrogen secretion (230). Mutations in *BMP15* or *GDF9* may negatively affect the FSH signaling, arresting folliculogenesis and causing POI (231). *FSHR* genetic defects frequently alter ovarian development in women with highly variable POI clinical manifestations (PA to SA), depending on the degree of resistance to FSH action in granulosa cells (27). *FSHR* mutations lead to POI when both alleles are affected and represent the first genetic cause that was linked to POI in the Finnish population (232). Other TGFβ-like growth factors produced by GCs have instead inhibitory roles on follicles development, such as AMH and INHA. AMH is a secreted factor that can either interact with its receptor AMHR2, mainly expressed in the adjacent mesenchyme, or circulate and play a role in controlling the gonadotropin-releasing hormone (GnRH) in the hypothalamus (233). *Amh* mutant female mice show accelerated ovarian primordial follicle recruitment, despite morphologically normal ovaries, suggesting a suppressor role in regulating germ cell development. AMH expression in developing follicles is highly dynamic: it starts in primary follicles, increases in preantral and small antral follicles, and then decreases in pre-ovulatory follicles, but not in CCs. Polymorphisms in this gene are associated with the age at menopause (234) but are rarely described in patients with POI, similar to mutations in *AMHR2* (235–237). INHA inhibits FSH production in the pituitary gland and genetic screenings in POI series revealed variations potentially associated (114, 238). Theca cells (TCs) originate from the ovary stroma, develop a blood supply and surround the follicle. TCs further differentiate into theca *externa* and *interna*, which develops LHCGR receptors and provide androgen hormone secretion. FSHR and LHCGR receptors are targets of the gonadotropins FSH and LHCG, respectively, both produced and released by the pituitary gland upon the hypothalamic stimulus. In turn, gonadotropin-dependent development allows the formation of antral and ovulatory follicles (239). *LHCGR* was among the first genes for which variations were related to POI (240), but its involvement in the pathogenesis of POI is rare. Female mice *Lhcgr* ^-/-^ are infertile with decreased estradiol and progesterone levels (241); however, after WT ovary transplantation, they can be fertilized (242). Women carrying homozygous pathogenic *LHCGR* variants usually exhibit normal secondary sex characteristics but may have delayed or absent menarche; however, heterozygous mutations might contribute to POI phenotype if combined with other genetic variants (106). Interestingly, infertile women carrying mutated *LHCGR* variants, coding for proteins with absent cell surface localization and signal transduction abilities, achieved successful oocyte retrieval and high-quality embryos leading to live births, indicating that *LHCGR* defects disrupt late folliculogenesis events and ovulation but have no effect on fertilization or embryo development (107).

Steroid hormone receptors, ESR1 and AR, are positive regulators of follicular maturation. ESR1 is a nuclear receptor expressed by TCs and GCs at this stage of follicular development, regulating growth and maturation to antral stage. *Esr1* ^-/-^mice present folliculogenesis blocked before antral formation or fail to ovulate and are infertile (243). Polymorphisms in *ESR1* have been associated with increased risk of POI (89, 244). AR is present in GCs and its deficiency in female mice may lead to dysregulation of important genes involved in folliculogenesis causing a POI-like phenotype (245), but few mutations of this gene have been linked to POI so far (144, 246).

##### 3.1.1.7 Antral follicles formation

Endocrine and paracrine factors from the hypothalamic-pituitary axis together with precise interactions among oocytes and GCs/TCs act in concert for antral follicle formation. Pre-antral follicles continue to extend their diameter, stimulated by FSH action and by the oocytes production of cumulin, and develop a fluid-filled cavity (antrum) with the oocyte eccentrically located in it. Simultaneously, oocyte further increases its volume, with cytoplasmic synthesis and accumulation of proteins, mRNAs, glycogen granules, ribosomes, mitochondria, and vesicles (161). Communication between the oocyte and GCs are facilitated by gap junctions, through which ions and small molecules pass mediated by Connexins, and filopodia-like structures with adherens junctions, where ligand-receptor interaction transduce the signal (i.e. KIT/KIT-ligand and Notch/Jagged) (247). Among connexins, GJA4 has a role in ovarian follicle development, and disruption of this gene in mice results in female infertility due ovarian folliculogenesis arrest at the preantral stage (248), but few variations have been reported in patients with POI so far (147). KIT-Ligand, a NOBOX target (249), activates the phosphatidylinositol 3-kinase/AKT pathway by interacting with its receptor (250). Variations in KIT/KIT-ligand cause ovarian insufficiency in rodents, but their role has yet to be elucidated in women affected by POI (53). Notch signaling regulates GCs proliferation (251): Jag1 ligand on oocyte interact with Notch2 and Notch3 receptors on GCs to activate the expression of target genes (i.e. Hey transcriptional repressors) (252). *NOTCH2* variants have been identified through WES in patients with POI (99, 128), while the role of other Notch genes in POI is still unclear. antral follicles are the most susceptible to atresia and may degenerate after GCs programmed death throughout apoptosis, autophagy (253), and ferroptosis (254). Increased circulating FSH levels and a fine balance of stimulatory and inhibitory growth factors, such as IGF1 and TGFβ family members, direct follicles to either atresia or development until ovulation (4). In monovulatory species, after puberty, just one dominant follicle, although several antral follicles are recruited, undergoes final development and maturation during each reproductive cycle, in response to cyclic hormonal changes (255, 256). Growing evidence suggests roles of miRNA in gonadal development, regulating genes involved in folliculogenesis, ovulation and steroidogenesis (257), although their functions and regulatory mechanisms remain inadequately understood (258).

##### 3.1.1.8 Ovulation and steroidogenesis

Ovulation is the fine-tuned remodeling process that ensures the follicle rupture when the uterus is receptive for embryo implantation. The dominant antral follicle rapidly grows to reach preovulatory stage (Graafian follicle) and produces higher levels of estradiol, that positively feedback to the hypothalamic-pituitary axis in a response required both for its further ovulatory process and for subordinate follicles growth inhibition after FSH levels lowering (259). Both FSH and estradiol signaling leads GCs to acquire LH receptors, thus the properly timed preovulatory LH surge from the pituitary gland activates the Graafian follicle and triggers a sequence of events that lead to ovulation: cAMP levels decrease in the oocyte with subsequent nuclear maturation (meiotic resumption), CCs mucificate and, at the end, oocyte-cumulus complex is released for fertilization while the remaining TCs and GCs of the ovulated follicle undergo dynamic transformation to become the corpus luteum, a progesterone producing structure needed for pregnancy (260). Conversely, inappropriate luteinization would impair follicle growth, reduce both the estradiol production in response to FSH and the negative feedback on ovary (261, 262). Previous studies provided evidence for the presence of luteinized Graafian follicles in the ovaries of women with karyotypically normal POI: in contrast with control population, in these cases a poor correlation between follicle diameter and serum estradiol levels and a failure in subsequent achievement ovulatory serum progesterone levels were found, suggesting that premature luteinization may be a major pathophysiological mechanism compromising follicle function in POI (263). However, the molecular mechanism underlying these pathophysiological processes are still unknown. The occurrence of luteinized Graafian follicles is the major source of the cyclic secretion of ovarian estrogens in women of reproductive age, in a joint two-cells system between TCs (that produce progesterone and precursor androgens under the control of LH signaling) and GCs (where FSH signaling regulates estradiol synthesis starting from androgens) (258). A recently identified player regulating this process of steroidogenesis is Epg5; its underproduction blocks autophagy in murine GCs and results in the accumulation of Wt1 transcription factor that finally leads to infertility (264). In humans, WES identified a rare *WT1* loss-of-function variant in a non-syndromic POI patient (265), and *NR5A1* is reported as a fundamental steroidogenic factor, whose variants were associated also with POI (81), either alone or in combination with other genetic variations (91). NR5A1 regulates the expression of *STAR, CYP17A1*, and *CYP19A1* (266), whose variations have been reported in syndromic POI. Another player with a role in estrogen production, *INSL3*, has been identified as mutated in a Brazilian patient with PA (164); Insl3 mouse KO promotes follicle atresia and disruption of female cycle (267). Similarly, targeted Pgrmc1 deletion in GCs suppresses antral follicles development and increased atresia (268) but its variations are rarely found in patients with POI and more research is needed (269, 270). *In vitro* studies point to a role of GATA4 in ovarian steroidogenesis by impairing estradiol synthesis (271), and conditional knockdown of Gata4 in mice showed female infertility. Variation of this gene has been found in two patients (99, 164). Recent data report that ovulation is initiated in mice by Progesterone receptor induction in GCs, which cooperate with RUNX1 to reprogram chromatin accessibility and alter gene expression (272). Noteworthy, the first *in vitro* model of GCs has been generated from human induced pluripotent stem cells (hiPSCs) after overexpression of NR5A1 and either RUNX1 or RUNX2; the procedure highlights the role of these transcription factors in folliculogenesis and is a central starting point in modelling several key ovarian phenotypes (273).

##### 3.1.1.9 Mitochondrial contribution

Oocyte viability and follicle maturation notably rely on mitochondrial biogenesis and bioenergetics (274, 275) and are the central sites for steroid hormone biosynthesis. Their swelling has been linked to GCs apoptosis and follicles atresia and, consistently, variations in genes involved in mitochondrial functions are responsible for POI (276), mainly syndromic cases. Mitochondrial defects due to mutations in the polymerase pol domain of the nuclear *POLG* gene predispose to Progressive external ophthalmoplegia (PEO) associated with POI (50, 277). Variants in *MRPS22* and *MRPS7* are respectively reported in isolated POI and in a patient with failure of pubertal development and hypogonadism (121, 278). We previously reported that the premature impairment of the ovarian reserve is associated with a significant decrease in the number of copies of the mitochondrial DNA (mtDNA) in blood cells and could then be considered a form of anticipated aging in which the ovarian defect may represent the first manifestation (279). The quantification of mtDNA in the peripheral blood could be used as a non-invasive biomarker for POI risk prediction (279, 280). The role of mitochondrial DNA content and of nuclear and mtDNA genes related to mitochondrial functions should be deepened with further investigations in larger populations, and with additional studies in model organisms.

### 3.2 Autoimmune causes

It is reported that autoimmune cover between 4 and 30% of POI cases (6, 281), however recent data suggest that number is closer to 4 to 15% (2, 282, 283). Dysimmune diathesis can be responsible for polyglandular diseases or result in oophoritis alone. Association with autoimmune diseases can be frequently found among women with POI (282, 284). The exact mechanism involved in the abnormal recognition by the immune system remains unknown, but both genetic and environmental factors are required for initiating the autoimmune response. The proofs for an autoimmune etiology are presence of lymphocytic oophoritis and associated autoimmune disorders (285), but this is not feasible in the clinical context. There are several reported target antigens involved in autoimmune oophoritis; importantly, among them, adrenocortical and steroidogenic autoantibodies, and in particular circulating 21-hydroxylase enzyme (21-OH-Ab), are recognized as the best markers of autoimmune POI (286, 287). A clear association between serum adrenal cortex autoantibodies and the presence of histologically confirmed autoimmune oophoritis was demonstrated (281). Between 2.5 and 20% of patients with POI result positive for adrenal autoantibodies, i.e. directed against the adrenal cortex (steroid cell adrenal antibodies, SCA-Ab) or the 21-OH-Ab (285). The other way round is also true, i.e. 10-20% of patients with Addison’s disease develop POI (6, 288). However, these antibodies are usually found in POI associated with autoimmune Addison’ disease, but they are not a frequent occurrence in non-adrenal autoimmunity or in isolated idiopathic POI (289). Anti-ovarian antibodies (AOAs) are found in 24-73% of patients with confirmed POI (285, 290), however, sources conflict in the accuracy of these findings and the exact prevalence remains unclear. Their role as a marker for POI is of no value due to the low specificity of existing tests leading to a high rate of false positive results and lack of validation (290). Autoimmune thyroiditis (defined as isolated finding of autoantibodies anti-thyreoperoxidase and/or anti-thyreoglobulin) appears be the most frequent pathology associated, with a percentage of patients suffering from clinical and subclinical hypothyroidism of 8-20% and up to 24% of POI cases, respectively (291). However, the prevalence of autoimmune thyroiditis in the female general population results nevertheless high, varying from 8.6% to 17.3% with prevalence increasing with age (292). Moreover, it is worth mentioning that a recent case-control study in 4302 euthyroid women with normal ovarian reserve and low ovarian reserve, had shown that among the whole population thyroid autoimmunity was not associated with low ovarian reserve but was significantly associated with overt POI in woman with TSH>2.5 mUI (293). Women with diabetes mellitus are also at higher risk of developing POI with an estimated prevalence of 2.5%. Therefore, fasting blood sugar or glycosylated hemoglobin can be recommended (2). POI has also been associated with numerous other disorders including rheumatoid arthritis, Crohn’s disease, myasthenia gravis, systemic lupus erythematosus, and multiple sclerosis (294). The different glandular diseases may then combine into different clinical and/or subclinical clusters, i.e. Autoimmune Polyglandular Syndromes (APS). In the specific case, patients with POI may fall into APS type 1, type 2 or type 3A in approximately 3% of cases (295). The APS type 1 typically develops in pediatric patients and is characterized by the presence of mucocutaneous candidiasis, Addison’s disease and hypoparathyroidism; auto-antibodies anti-steroidogenic cells can lead to lymphocytic oophoritis in 60% of cases. Type 2 APS is associated with Addison’s disease, type 1 diabetes mellitus, hypothyroidism (or Graves’ disease) and less frequently with POI (296). However, the causal association between autoimmunity and ovarian insufficiency remains difficult to establish, and the presence of autoimmunity (either clinically or biochemically) does not necessarily imply the autoimmune origin of the condition, also in view of the relatively broad prevalence of autoimmune disorders and the low specificity of autoantibody measurement. The autoimmune etiology of the POI can be consider substantiated in such case as the presence of adrenocortical and/or steroidogenic cell antibodies and autoimmune Addison’s disease (APS type 1 or 2), possible or probable in case of presence of autoantibodies and/or autoimmune disease other than autoimmune Addison’s disease (297). Interestingly, in terms of phenotype, it has been described that autoimmune oophoritis presents as a distinct clinical entity compared to women with idiopathic POI: the former were found to have significantly larger, and possibly multifollicular ovaries in association with elevated inhibin B values (281, 298).

### 3.3 Iatrogenic causes

Iatrogenic causes account for 6-47% of POI cases (2); they can in turn be distinguished into surgical forms, post-chemotherapy, or following radiotherapy (whether local or external, with exposures greater than 1 Gray). Also, common iatrogenic causes that lead to POI in the process of treating non-malignant gynecological diseases include uterine artery embolization and pelvic surgery for ovarian cysts, endometriosis, and ovarian torsion; in particular, it has been shown that excision of bilateral endometriosis can lead to POI in 2.4% of cases (299). Female survivors of childhood, adolescent, and young adult cancer, have an increased risk of POI, with a cumulative incidence of approximately 8% by age 40 years (300, 301). The effects of chemotherapy depend on the type, previous ovarian reserve, dosage, and age at administration (302, 303). Treatments with evidence of causing POI include alkylating agents in general, cyclophosphamide, procarbazine, and radiotherapy to which the ovaries were potentially exposed, in a dose-dependent manner (300, 304). Indeed, for at-risk pre- and peripubertal survivors the monitoring of growth and pubertal development and progression is strongly recommended. Whereas, for postpubertal women who were treated with alkylating agents and/or radiotherapy to which the ovaries were potentially exposed, is strongly recommended detailed menstrual history and physical examination, with specific attention paid to POI symptoms (300). Laboratory assessment should be performed only on the basis of clinical indication or when the patient desires valuation of potential future fertility, at least annually (300).

### 3.4 Other acquired causes

In 1% of the cases, POI may be related to toxic, metabolic or infectious causes (2). Regarding POI associated with a history of infectious diseases, there is some evidence to suggest that women affected with HIV experience menopause at an earlier age (305) and, although gonadal function is relatively understudied, data found that HIV-positive women were more likely to have lower levels of AMH, largely explained by lower CD4 counts (306). The parotitis virus that causes mumps and results in mumps oophoritis can lead to ovarian failure in 2-8% of cases; however, this tends to be transient in most affected women and normal ovarian function resumes after recovery (299). Anecdotal reports described other viral and microbial infections, such as tuberculosis, varicella, cytomegalovirus, malaria and shigella as causes of POI (2). Association with environmental and toxic causes is also described. Exposure to phthalates and bisphenol-A present in plastic production and other environmental pollutants has been suggested as a possible risk factor POI; these toxins have been shown to increase follicular depletion and accelerated atresia of preantral follicles resulting in an earlier onset of menopause (307). A 2024 meta-analysis described that environmental pollutants pose a serious threat to human and animal reproduction. Such substances, including persistent organic pollutants, heavy metals, phthalic acid esters, polycyclic aromatic hydrocarbons, cosmetic and pharmaceutical products and cigarette smoke, are indeed significant risk factors for POI, with pooled OR of 2.331 (308). Also, preclinical studies speculated that environmental pollutants lead to POI via improper hypothalamic-pituitary-gonadal axis functioning, changed follicular mRNA/hormones, reduced ovarian volume and obvious follicle atresia (308). Moreover, a relation between cigarette smoking and early menopause has been described, although no direct causal relation has been confirmed (295). However, women who are prone to POI should be advised to stop smoking (309).

## 4 Clinical presentation and diagnosis

Typically, POI may present as menstrual irregularities or secondary amenorrhea, associated with infertility and hypoestrogenism symptoms, such as hot flushes and night sweats, vaginal dryness and dyspareunia, diminished libido and sleep and mood disorder. They generally are more pronounced than those typical of climacteric, especially in acquired forms with sudden onset. In those cases with early onset it may occur as primary amenorrhea with varying degrees of pubertal development, eventually associated with gonadal dysgenesis. Associated symptoms and clinical findings can be variable due to intermittent production of ovarian hormones. In fact, it is worth emphasizing that it may be associated with intermittent resumption of ovarian activity in over 25% of women (310, 311). Estimates of the likelihood of spontaneous pregnancy vary widely in the literature, but from the available data it appears that about 5% of women with POI will conceive naturally. Most of these conceptions will occur within a year of diagnosis, but pregnancies have been reported many years later (312). Despite the clinical marks, studies report that up to 70% of women with POI have ovarian follicles remaining in the ovary (262, 263), one-half demonstrated ovarian follicle function and, remarkably, 16% of these women achieved an ovulation (263). From a biochemical point of view, POI results in hypergonadotropic hypogonadism, which represents the diagnostic cornerstone. European Society of Human Reproduction and Embryology (ESHRE) guideline recommended that in case of oligo/amenorrhea for at least 4 months diagnosis is confirmed by two elevated FSH tests (>25 U/L), 4–6 weeks apart (309), performed on precocious follicular phase (day 2–3 of the cycle) if menstrual flows are still present. Elevated FSH must be associated with low estradiol level, to rule out the possibility of gonadotropins pre-ovulation peak. In addition, another useful marker is represented by the serum anti-Mullerian hormone (AMH) levels. It is a hormone produced by granulosa cells of growing follicles (<8 mm in diameter) (313, 314), whose concentration reflects the number of follicles remaining in the ovary (315). It has emerged as the current best biomarker of the primordial follicle pool constituting the ovarian reserve (316), and it is known that AMH declines before the menopause in advance of elevated FSH concentrations (317). AMH generally results in low/undetectable (318–320), although there are still no defined cut-offs for diagnosis (6). A small number of studies that have investigated the value of AMH in the diagnosis of POI, demonstrating a progressive decline in women across the stages of deteriorating ovarian function to POI, although data estimated that AMH is detectable in approximately 6% of the POI population (321–323). The largest study to date suggested that an AMH of ≤ 0.25 ng/ml (1.78 pmol/l) was diagnostic of POI with high sensitivity and specificity (323). Furthermore, it was reported that AMH concentrations were lower in women who experienced primary amenorrhea than in those with secondary amenorrhea (324). A transvaginal ultrasound scan can also be helpful in estimating ovarian status. The ovaries can be found to be compact and small, and up to 50% of primary amenorrhea cases may have gonadal dysgenesis with “streak” ovaries. However, as mentioned above, it is not uncommon to detect evidence of ovarian function (262) (pre-antral, antral follicles or pre-ovulation follicles and/or ovarian corpus luteum). Thus, ultrasound findings can be misleadingly reassuring regarding ovarian function and fertility prognosis (325), but may also be useful in revealing any remaining ovarian activity. Ovarian reserve can be assessed sonographically by antral follicle count (AFC), that would be expected to be low in POI and usually correlate with AMH levels. Occasionally relatively normal AFCs are seen despite low AMH levels, however, AMH appears to be a stronger predictor of ovarian response (316). Unfortunately, because of the above, when the biochemical criteria of POI are met, the ovarian reserve is found to be already substantially reduced or follicular dysfunction is present with impaired responsivity to FSH (262, 263), consequently, the chances of fertility preservation are severely diminished (323). For this reason, the identification of early diagnostic markers to identify women at high risk of developing POI, enabling effective fertility planning, is of great interest. However, while it has been postulated that AMH may be of value in assessing family members of a proband with POI (326), current evidence showed low discriminatory performance of AMH in menopause prediction in young women (316, 322, 327). Once the diagnosis of POI has been confirmed, it is important to carry out investigations to establish the origin of POI. After ruling out possible acquired causes by means of a thorough medical history, autoimmune and genetic causes should be sought in the first instance. ESHRE guidelines recommend routine screening for the presence of thyroid autoantibodies and 21OH-Abs in every case of POI. Screening for anti-ovarian antibodies is not recommended. Screening beyond Hashimoto’s and Addison’s diseases is not routinely performed (309). On the other hand, in patients with multiple autoimmune diseases or Addison’s disease it should be advisable to consider screening for early detection of POI. Also, measurement of fasting blood glucose or glycosylated hemoglobin levels should be performed (2).

### 4.1 Genetic diagnosis

Turner Syndrome (45,X), mosaicisms of X chromosome, the partial loss of critical terminal regions of the long arm of the X, and X-autosomal translocations are well-known chromosomal abnormalities causing POI that could lead to either primary or secondary amenorrhea (328). Besides, one of the major genetic alterations implicated in POI is being a carrier for the FMR1 gene premutation. In women, this condition defines a higher risk (>20%) of developing the premature exhaustion of ovarian function, which can also be associated with other symptoms (i.e. ataxia, psychomotor developmental disorders, and cardiovascular pathologies). Additionally, premutated alleles are mitotically and meiotically unstable and could lead to the expansion to full mutation allele during the maternal transmission and cause the Fragile X syndrome in male offsprings of the next generation. The prevalence of premutated FMR1 allele in the general female population is estimated to be around 1:250 and even higher among different ethnic groups (329). Investigating the FMR1 premutation is of primary importance in patients with POI because the chance of evolving to the full mutation (>200 repeats) in the subsequent generation is close to 100% for expansions >100 repeats (330). Fragile X syndrome is characterized by dysmorphism, severe intellectual disability and autism in males. Therefore, first-level tests for the clinical and genetic evaluation of POI are high-resolution karyotype (that we suggest as first-line genetic test in PA and in SA cases <30 years of age) on 2 independent cultures by analyzing at least 30 metaphases (at 400–550 band resolution), according to the International System of Chromosome Nomenclature 2020 (ISCN 2020) (331), which can be extended to 100 in case of mosaicism and eventually replicated on a cutaneous biopsy, and the molecular analysis of the FMR1 gene; its involvement is highly unusual in PA cases while more frequent in SA cases >25 years of age. In case of negative or uncertain results for both FMR1 and karyotyping, Comparative Genomic Hybridization arrays could be performed to identify undetected chromosomal duplications/deletions or low level mosaicism (<10%). The further genetic screenings should be guided by the presence of clinical features suggestive of a specific syndrome. In the absence of any syndromic phenotype, *ad-hoc* target Next Generation Sequencing (NGS) panels of candidate genes can be used to identify single nucleotide variants potentially pathogenic. Within the NGS strategies, the analysis of families with POI and large POI series through whole exome sequencing or whole genome sequencing can be used to identify new variants and novel genes involved in the pathogenesis of the disorder and their application is considerably improving our understanding of the molecular basis of ovarian functions and dysfunctions (332). NGS has shed light on the role of oligogenicity as a significant contributor to the genetics of POI (105, 332). Discerning true oligogenicity from digenicity or rare, potentially deleterious variants associated with POI remains a significant challenge. Oligogenicity refers to the involvement of multiple genes in a phenotype, while digenicity specifically involves interactions between two genes. In the context of POI, understanding these genetic complexities is crucial for accurate diagnosis and counselling. Another significant challenge in the genetic diagnosis of POI is the huge number of variants of unknown significance that emerge from NGS. It is likely that several heterozygous VUS can predispose to POI in the context of an oligogenic or multifactorial origin. In the future, efforts should be directed toward a more precise genotype-phenotype correlation and the resulting causative relevance. These challenges are further complicated by the complex gene network involved in POI, as well as the environmental component. Further, in the same family, the occurrence of members affected by POI or early menopause could be observed, due to incomplete penetrance. Investigating the genetics of the pathogenesis of POI is essential to deepen ovarian physiology and to solve the pathogenic mechanisms involved. As we uncover novel pathogenic variants, genetics can serve as valuable tools for precise diagnosis, predicting POI risk in families, and furnishing improved genetic and reproductive counselling that help women to plan their fertility.

## 5 Clinical management and long-term consequences

POI has a multisystem impact with profound physical and emotional implications; as such, its optimal management should be handled by a multi-disciplinary team. Of course, the effects of the condition on quality of life depend on the age of onset, the underlying cause and inter-individual variability. Age at menopause has been shown to have an additive effect on all-cause mortality and an independent predictor of subsequent cardiovascular outcomes (333–336). In particular, regardless of etiology, POI increases the long-term risk for cardio-metabolic disease (337): in this metanalysis emerges that compared to women with menopause at age >45 years, women with POI had a higher risks of type 2 diabetes (RR: 1.32, 95% CI: 1.08–1.62), hyperlipidemia (RR: 1.21, 95% CI: 1.05–1.39), coronary heart disease (RR: 1.52, 95% CI: 1.22–1.91), stroke (RR: 1.27, 95% CI: 1.02–1.58) and total cardiovascular event (RR: 1.36, 95% CI: 1.16–1.60). In fact, hypoestrogenism exerts several deleterious effects on many contributor factors, including lipid profile, insulin resistance, centripetal obesity, chronic inflammation, hypertension, vasoconstriction, endothelial dysfunction, and autonomic nervous system dysfunction. It is interesting to note that in this large Chinese study (338)the Authors found that POI increased the risk of total and cancer-specific mortality (HR (95%CIs): 1.29 (1.08–1.54) and 1.38 (1.05–1.81), respectively), while decreasing incidence of breast cancer (OR (95%CI): 0.59 (0.38–0.91)), but they didn’t find any statistically significant association of POI with either mortality or morbidity related to CVD. Similar results were observed when HRT users were excluded from the analysis Nonetheless, in many of the reported studies women who used or had used HRT were excluded from analysis or data on HRT use was not available. Thus, data are lacking to demonstrate whether the results were independent of HRT. However, cardiovascular protection seems to be related to exposure time (339), with the greatest reduction in CVD incidence in women who used HRT for at least 10 years and within 1 year of diagnosis (336), and type of HRT (340). Given the well-characterized cardiometabolic and bone impact of POI, optimal management of this condition should include baseline assessment of insulin resistance and lipid profile. Monitoring of these parameters should be dependent on comorbidity, personal and family history; however annual assessment of cardiovascular risk markers may be appropriate, although evidence on cost-effectiveness is currently lacking (6).

Women with POI have a significantly lower bone mineral density (BMD) (341–343) and a 1.5- fold greater risk of fracture compared to women who experience menopause at the typical age (344), with an estimated prevalence of osteoporosis of approximately 8–27% (342, 345, 346), mainly due to insufficient acquisition of peak bone mass (in those with primary amenorrhea or early onset) and increased bone resorption associated with estrogen deficiency (347), with greater loss of trabecular bone than cortical bone. Identified risk factors for low BMD included young at onset of irregular menses and delay in diagnosis greater than one year and/or lack of compliance with HRT. Indeed, the most important risk factors contributing to BMD loss in POI are the degree and duration of estrogen deficiency. Also, it may contribute the presence of comorbidities and risk factors related to the specific etiology, e.g. women with Turner Syndrome have additional contributors to bone loss, skeletal fragility and falls risk; including genetic abnormality, coeliac disease, hearing impairment and visuo-spatial abnormalities (348, 349) while autoimmune conditions associated with POI may also directly contribute to bone loss. Dual-energy X-ray absorptiometry (DEXA) examination should be performed at diagnosis in all young patients with amenorrhea lasting more than 6 months as a result of hypoestrogenism (309). The frequency of bone densitometry should be evaluated according to the presence of other risk factors for osteoporosis, BMD at baseline, and its change with time (6), however in women with POI with low BMI, and treatment initiated, a repeat DEXA scan in 2–5 years to monitor response is recommended (309, 348). Nevertheless, DEXA has some limitations in the context of patients with POI: it can generally not be used until peak bone mass has been achieved; it does not differentiate between cortical bone and trabecular bone; it does not provide any information on bone quality or geometry; it underestimates BMD in women with short stature, such as Turner syndrome (TS) (345, 350). However, it has been proposed that Z score<−2 should be used to define low bone mass in pre-menopausal women, though, maintaining the use of T- score<−2.5 to diagnose osteoporosis in young adults suffering from chronic disorders known to affect bone metabolism (351). In the absence of more specific tools, trabecular bone score (TBS) could be is a promising adjunct to DEXA for guiding the evaluation of POI-related bone health (343).

Mental health sequelae can be explained primarily by estrogen deficiency, along with the denial or difficulty faced by diagnosed women in accepting the loss of fertility (312, 352). It has been demonstrated major prevalence of anxiety and depression in women with POI (353). Such psychological distress may not only negatively influence the treatment outcomes of these patients, but it can also cause social life disruption and issues in the affective and relational area and even social isolation (354, 355).

The inadequate levels of estrogen present in POI also result in symptomatic vulvovaginal atrophy and urinary incontinence, collectively defined as genitourinary syndrome of menopause (GSM). GSM is associated with symptoms such as dryness, itching, burning, irritation, decreased discharge, dysuria, urinary frequency and urgency, and recurrent urinary tract infections (356). Furthermore, women with GSM report low sexual desire, reduced orgasmic function and dyspareunia (357, 358). Sexual dysfunction and hypoactive sexual desire disorder (HSDD) can be attributed as well as the synergistic effect of estrogen and androgen deprivation related to reduced stimulation of sexual responses (359)). Personal, family, and psychosocial factors may further determine the severity of sexual impairment (360). In women diagnosed with POI, GSM and HSDD have a more detrimental effect on sexual function, body image and overall quality of life compared to women who experienced physiologic menopause (361, 362). However, some evidence showed that the arousal and lubrication domains are the most influential factors of sexual function among women with POI, while the desire domain played the lowest role. Additionally, GSM-related symptoms have a greater impact on quality of life compared to vasomotor symptoms, likely due to the fact that genitourinary symptoms deteriorate if not treated whereas vasomotor symptoms usually improve over time (363).

Education on a well-balanced diet with adequate physical activity in order to maintain an adequate weight, avoiding smoking and minimizing alcohol consumption is particularly important (6). Calcium and vitamin D supplementation are equally important. Women with inadequate dietary intake should take supplementary elemental calcium intake (diet and supplements) is approximately 1200 mg/day (350).

### 5.1 Hormonal replacement therapy

Women with POI experience symptoms due to low estrogen levels; however, whereas in the case of women who have gone through menopause at a physiological age HRT is aimed at alleviating symptoms due to hypoestrogenism, in women with POI HRT’s fundamental purpose should be to restore physiological estrogen levels, in line with patients’ age. Indeed, women should be informed that HRT may play a role in primary prevention of long-term consequences (309, 340, 364, 365). Estrogen replacement therapy has, therefore, multiple goals: to induce the development of secondary sexual characteristics (including uterine growth) in prepubertal girls with primary amenorrhea, alleviate typical vasomotor symptoms (VMS), urogenital problems due to vulvovaginal and bladder atrophy, mood/cognitive problems, reduced energy levels and musculoskeletal pain, and, finally, reduce long-term complications such as cardiovascular disease and osteoporosis. To optimize the achievement of the therapy, the choice of treatment regimen is therefore crucial. Among available formulations, hormone preparations based on 17β-estradiol are preferable because they are more physiological, safe, and effective than those based on ethinyl-estradiol and conjugated equine estrogens (366), especially when administered transdermally. The primary advantage of transdermal administration is that it bypasses the hepatic first-pass effect, reducing liver exposure to supraphysiologic doses of estrogen and the resulting increase in pro-coagulant factors, SHBG, triglycerides, and markers of inflammation (367), worsen insulin resistance, particularly in obese women who have an increased risk profile at baseline (368). Unfortunately, there are no products specifically designed for the long-term treatment of young women with POI, thus formulations marketed for the treatment of climacteric disorders in postmenopausal women or estro-progestin combinations for contraceptive purposes (COCs) are commonly used. The still widespread use of COCs (367) could certainly offer the advantage of greater patient acceptance, however, mounting evidence shows that its use has a less favorable impact on bone and cardiovascular health, as well as on uterine development. More physiological regimens should therefore be preferred, and synthetic estrogen-containing formulations should be avoided (328, 340, 366, 369, 370); even though the use of COCs containing estradiol valerate as HRT in POI is also off-label and requires further research. However, it must be recognized that much of the current evidence on HRT is not gained from studies specifically conducted on women with POI, but comes from extrapolations on data available for physiological menopausal women or from non-randomized controlled studies. Treatment with 17β-estradiol should be continuous to avoid periods of hypoestrogenism (371). Recommended doses should be generally higher than those used in postmenopausal replacement therapy (6), with the rationale of restoring estrogenic values typical of childbearing women, as a dose-response effect of estrogen regarding cardiovascular and bone benefits has also been suggested (340, 372, 373). HRT maintains or increases BMD at the lumbar spine, femoral neck and total hip, with the magnitude of response dependent on the POI etiology or HRT regimen used, being physiological estradiol more beneficial than synthetic estrogens (346, 374, 375). In particular, in the only one long-term, prospective, double-masked, controlled study on HRT in women with POI data showed that physiologic HRT (estradiol patch 100 mcg/d and cyclical oral medroxyprogesterone acetate 10 mg/d for 12 days of month) restored bone mineral density to normal over three years, being well tolerated and increasing estradiol levels to those of a control group of women with normal ovarian function (376). Higher estrogen doses (2 mg oral or 100–150 mcg/d transdermal estradiol) have been found to be superior in increasing BMD compared with lower doses or COCs (369, 377). In addition, transdermal estradiol was found to be associated with a better impact on BMD than oral estradiol (378, 379). However, to date studies available on the optimal regimen for women with POI are still lacking and future properly designed trials should aim to compare results to the validated regimens (376). HRT regimens must be individualized, carefully balancing benefits, risks, and side effects. It may be necessary to start with lower doses to test tolerance and increase the dose until the optimal dosage is reached. Examples of the dosages to be used are given in **Table 4**.

**Table 4:** Formulations and regimens for hormone replacement therapy.

Estrogen therapy		Progestin therapy		
Formulations	Doses	Formulations	Sequential regimens*	Continuative regimens**
Estradiol valerate mg (tablets)	2 - 4	Micronized progesterone mg (caps)	100+	200+
Estradiol mcg/24 (patchs)	50 - 150	Dydrogesterone mg (tablets)	5+	10+
Estradiol/Estradiol emiidrate mg (gel)	1 - 3	Medrossyprogesterone acetate mg (tablets)	2.5+	5+

*given for 14 day per month, **given every day.

Treatment with progestin is necessary in women with uterus to prevent endometrial hyperplasia and minimize irregular bleeding. Micronized natural progesterone, taken orally or vaginally, has demonstrated greater safety than traditional synthetic progestins (such as medroxyprogesterone acetate) regarding breast cancer risk, metabolic impact, and thromboembolic events (380, 381) and, therefore, is the first choice recommended by the most recent guidelines. Dihydrogesterone, commonly used in oral preparations in fixed combination, has similar metabolic and mammary benefits.

HRT regimen can be either combined sequential, with the introduction of progesterone in the second phase of each cycle (for 12 to 14 days), or combined continuous, with progesterone taken at lower doses but throughout the entire month. In the former, if the estrogen dose is sufficient to thicken the endometrial mucosa, there are menstrual-like flows induced upon discontinuation of progestin. The continuous combined scheme results in almost complete atrophy of the endo-uterine mucosa resulting in absence of bleeding in most cases. Endometrial protection seems to be greater for continuous administration (382), however, the risk of breast cancer appears to be increased with the latter in postmenopausal women (383). The choice between the two types of administration also depends on the patient’s preference. It is worth mentioning that it should be advisable to opt for a sequential combined regimen if the woman plans a pregnancy or if fertility treatment with egg donation is planned before long. It is also important to be aware that if doses of estrogen higher than standard are prescribed it may be necessary to adjust the progestin dosage using higher doses, depending on the clinical findings and the pattern of bleeding. Finally, the use of levonorgestrel-medicated intrauterine dispositive (IUD) can be suggested (in combination with transdermal or oral estrogens), especially if contraception is needed or in cases of irregular vaginal bleeding. It provides durable endometrial protection, with the advantage of resulting in negligible systemic concentrations of progestin and, therefore, fewer side effects than systemic therapy (384). The use of COCs should be considered if the patient is likely to have residual ovarian reserve and desires contraception. COCs provide contraception, menstrual cycle control and relief from VMS and other symptoms. Occurrence of VMS during the use of ethinylestradiol COCs that may be alleviated switching to an estradiol-containing COCs. VMS in the pill-free week can be managed by eliminating the placebo (385). In recent years, an estetrol-based contraceptive has also become available, appearing to have more favorable effects on metabolism and blood pressure (386). Women can transition toward HRT if contraception is no longer required, opting for a combined sequential therapy. However, these regimens do not suppress ovulation, therefore women with enduring ovarian function may frequently experience symptoms of estrogen excess including mastalgia and erratic bleeding due to their underlying ovarian function (385). COCs therapy can be also switched when the risk of an unwanted pregnancy is highly unlikely, typically >2 years after diagnosis (6). It is worth mentioning that some Authors proposed that, taking into account that inappropriate follicle luteinization due to the tonically elevated serum LH levels is recognized to be a key mechanism of follicular disfunction (262, 263), a trial of physiologic HRT, by reducing LH levels, may improve follicle function and increase the chance of ovulation and spontaneous pregnancy in some women with POI (387).

Concerning patient with primary amenorrhea, puberty should be induced or progressed with 17β-estradiol, preferably in the transdermal form, starting at the age of 12 with a low dose of approximately 10% of the adult replacement dose and increased every 6 months over a 2 to 3-year period (309, 328, 388, 389). After about 2 years of unopposed estrogen, or if more than one episode of significant breakthrough bleeding occurs, it is necessary to introduce a progestin to induce withdrawal bleeding. The progestin should not be added until there is substantial breast development, because premature initiation of progestin therapy can compromise ultimate breast growth, other than uterine maturation (328). Indeed, uterine maturation is a prerequisite for patients who want to carry on a pregnancy. Unfortunately, more than half of the patients with hypogonadism undergone to pubertal induction were found to have a suboptimal uterine outcome (390), and the risk appears to be greater in girls who had received pelvic irradiation (fibrotic damage to the uterine structure) or with Turner’s Syndrome (96). Emerging data showed that progestins, hampering further changes in uterine volume and breast development, should be introduced only in the presence of a concomitant adequate 17β-estradiol dose and an appropriate clinical response (96). Overall, the approach must be individualized, depending on the specific characteristics, circumstances and desires of patients. When hypogonadism is diagnosed late, or it develops after spontaneous pubertal start, estrogen dosing regimens can progress more rapidly, especially for those in which final height is not a concern (389, 391, 392). Guidelines (309, 328, 393) recommend that replacement therapy continue at least until the average age of menopause (51 years). Thereafter, the risk-benefit ratio and individual symptomatology should be evaluated on an individual basis. However, it should be taken into account in clinical practice that a number of women with POI have received inadequate treatment previously or have had prolonged interruptions in therapy, and therefore should be encouraged to continue for longer.

HRT is indicated in all women with POI, except where specific contraindications exist. The only absolute contraindication is in the patients surviving an estrogen-dependent cancer in young age. A personal history of breast cancer contraindicates the use of hormone replacement therapy (6, 309, 394). However, it should be noted that the risk of breast cancer with long-term use of estro-progestin therapy in POI is not higher than that of the age-matched general population (395–397). The use of HRT for the management of menopausal symptoms in carriers after risk-reducing bilateral salpingo-oophorectomy (rrBSO) is controversial since data from the few available studies are conflicting: guidelines approach this issue differently, with some recommending offering HRT while others suggest considering and discussing individually risks and benefits with the patients (398, 399). Regarding other tumor types, whose treatments may also lead to POI, HRT is indicated in most conditions but potentially harmful in some hormone-dependent malignancies (e.g., uterine sarcomas, ovarian cancer, meningioma or ER+/PR+ gastric carcinoma). Indeed, in some cancers or their subgroups, the risk of HRT may outweigh the potential benefits and careful individualized decision making is needed (e.g., some ovarian cancers) (394, 400). It also is worth mentioning that an increased thromboembolic risk is not an absolute contraindication to HRT but requires more caution. Women with a history of prior thrombosis or thrombophilic disorder should be evaluated by a hematologist before starting HRT. Screening for thrombophilic disorder should be performed only in patients with a personal or family history of thromboembolic events. Transdermal estradiol is the preferred route of administration for women at increased thromboembolic risk (309). Some clinical conditions, where there is a relative contraindication for postmenopausal contraceptive or replacement therapies, such as migraine, hypertension, or obesity, are not contraindications for HRT in POI. Even in these cases, transdermal administration is preferred for its lower hepatic effect (6, 309).

Once HRT has been established, women with POI should be clinically monitored once a year, to check compliance, satisfaction, side effects, and whether the regimen or route of administration needs to be changed. No routine monitoring tests are required but may be prompted by specific symptoms or concerns (309). Monitoring estrogen levels may be useful to assess the appropriateness of therapy, having as a target the average values typical of women of childbearing age (100-150 pg/mL) (328, 373, 401). However, there is currently no consensus on the usefulness of this practice. Likewise, there is no indication for monitoring FSH, although it has been shown to correlate with bone loss. Still, available data showed that serum FSH levels in women on physiological hormone replacement therapy were still significantly higher than control levels (376). Also, markers of bone turnover (BTMs) could potentially be used to monitor adequacy of the HRT dose, as decreases in BTM are observed in clinical trials of HRT in women with POI; however, this is not establish in current guidelines (309, 346).

### 5.2 Other therapies

If genitourinary symptoms persist despite systemic HRT, low-dose vaginal estrogen or prasterone can be additionally administered (6, 402). In addition, some women with POI may benefit from androgen replacement, as it is recognized that the levels of women with POI have lower androgen levels than age-matched controls (403), possibly adversely affecting sexual desire and function and physical performance. Nonetheless, data showed that the addition of physiological transdermal testosterone replacement did not provide additional benefit in BMD increase (376). Currently, the only indication for testosterone use in women is postmenopausal hypoactive sexual desire disorder (404). However, the lack of licensed treatment options that can easily provide the required physiological dose of 5 mg/day of testosterone makes it challenging to use this therapy.

Non-hormonal pharmacological options are indicated for the alleviation of vasomotor symptoms in cases where HRT is contraindicated (405, 406). Low doses of antidepressants drugs are effective for the relief of hot flashes are lower than those commonly used for the treatment of depression, with onset of relief generally occurring. Paroxetine mesylate (7.5 mg per day) is the only nonhormonal treatment for vasomotor symptoms that has been approved by the FDA. Trials have shown a similar reduction in vasomotor symptoms with low doses of oral estradiol (0.5 mg per day), venlafaxine XR (75 mg per day), and escitalopram (10 to 20 mg per day) (407). Neurokinin 3 receptor (NK3R) antagonists, as Fezolinetant, have recently been developed as novel therapeutic agents for the amelioration of VMS through their action on NK3 receptors within the hypothalamus and consequent regulation of the thermoregulatory center. Fezolinetant has demonstrated significant reductions in VMS frequency and severity, improving transform patients’ quality of life (408, 409). In order to alleviate genito-urinary symptoms, vaginal moisturizers and lubricants can be used.

Bisphosphonates should be avoided in this young population because of the potential desire for pregnancy and the possible need for long-term use with associated reduction in bone turnover. However, bisphosphonates may be necessary if HRT is contraindicated or considered if there is high risk of fracture despite optimal hormone therapy (6, 345, 348, 350).

### 5.3 Fertility preservation and future prospective

Fertility preservation techniques have made great advancement in the last years. While embryo and oocyte cryopreservation are now considered as established techniques, several positive data are now available on ovarian tissue cryopreservation (OTC), and new innovative technique like *in vitro* maturation of oocytes and *in vitro* activation are beginning to be explored with success. Nevertheless, in women with overt POI, the opportunity for fertility preservation is still missed, due to the loss of most of the reproductive material (410). In this case, recent guidelines still report oocyte donation as the only realistic therapeutic option for a pregnancy (6, 202, 309). Indeed, the possibility of preserving fertility strongly depends on an early diagnosis. However, current guidelines do not recommend specific indication on fertility preservation in patients with genetic risk of POI (202). The ESHRE guideline for female fertility preservation (410) recommend individualized counselling for women with a poor ovarian reserve (AMH <0.5 ng/ml and AFC <5) as the value of fertility preservation is unclear, adding, nevertheless, that OTC should not be offered to most of these patients considering the poor success rate. Still, more studies are needed to guide counseling and decision-making in patients seeking these services despite a poor ovarian reserve (411). For girls diagnosed with TS, karyotype-based management has been proposed: Oktay et al. suggest fertility preservation in patients with monosomal karyotype 45, X (in whom the ovarian reserve may be exhausted within the first few years of life) as soon as the diagnosis is made, even in childhood. For mosaic patients, on the contrary, it is proposed to delay fertility preservation to the post menarche age, if possible, based on ovarian reserve monitoring (328, 412). Same Authors state that ovarian reserve can be exhausted if AMH < 0.1 ng/ml, AFC 0 at ultrasound, and FSH > 30 IU/L, whereas it can be considered severely diminished if AMH < 1.1 ng/ml, AFC < 7, and FSH 12–30 IU/L, although evaluation should always be individualized (413). Cryopreservation of ovarian tissue and subsequent ovarian transplantation is identified by several authors as the most appropriate technique for prepubertal girls at risk of POI. This technique has been recently recognized as an established technique in cancer patients (414). Nonetheless, given the limited evidence existing, it should still be considered experimental in patients with idiopathic POI (412, 415, 416). However, given the frequent familiarity for POI and the improved sensitivity of NGS in identifying heritable predisposing variants in a woman with idiopathic POI, we propose the investigation of the carrier status in the young female relatives of an index case. The OTC could then be proposed only to the young relatives carrying the predisposing variant at the preclinical stage of POI, when FSH and AMH levels are still in the normal range and the ovarian reserve is still intact or not completely exhausted.

It is hopeful that in the future, it will be possible to associate to the OTC technique the *in vitro* maturation (IVM) and activation (IVA) of small antral follicles retrieved *ex vivo* from the taken ovarian tissue (202, 417–422). In this view, knowledge of underlying molecular processes of POI can be particularly useful: in fact, patients who harbor a defect in genes involved in the initial phases of ovarian reserve development will more likely present clinically ovarian dysgenesis or reduced pool of primordial follicles, while for mutations that impair the most advanced stages of follicle development, the presence of a residual follicular pool could be assumed. Moreover, specific clinical and ethical issues concerning possible association with syndromic clinical conditions need to be taken into consideration. However, to date, there are no established treatments to increase the chance of conception, although many approaches have been investigated. Several approaches involve the use of mesenchymal stem cells (MSC) (423), from a variety of sources including bone marrow, placenta and umbilical cord, that had shown to have efficacy in animal models of POI (424). Another approach involves the use of Platelet-rich Plasma (PRP), an autologous blood derivative with high concentration of growth factors (GFs) that can act in a paracrine manner, mediating tissue regeneration and homeostasis. According to preliminary data in animal models of POI, intraperitoneal or intraovarian administration of PRP is demonstrated to increase ovarian cortex volume, AFC and ovarian function, with recently emerging data from few clinical studies in women with POI support the efficacy of PRP in ovarian rejuvenation (425, 426). As mentioned, activation of primordial follicles (IVA) through physical or chemical manipulation of key regulatory pathways (notably the phosphoinositide 3-kinase (PI3K)/AKT/mammalian target of rapamycin (mTOR) and Hippo pathways) is also being developed (418–422). The latter two techniques appear to be quite effective in achieving IVF conception, with rates of 4% and 7%–8% respectively (426). However, currently, much of the clinical data are uncontrolled, and mostly in women with a reduced ovarian reserve rather than POI. Further studies are needed to substantiate the preliminary claims of success of these approaches (415, 426, 427).

Lastly, another thrilling area of current research which may change the course of fertility preservation treatments is human *in vitro* gametogenesis (IVG). IVG is the process of generating gametes in a dish, starting from pluripotent stem cells. Induced pluripotent stem cells (iPSCs) can be used to reproduce *in vitro* the process of gamete formation. iPSCs can be derived from any differentiated somatic cell, such as skin fibroblasts or peripheral blood mononuclear cells, and can be reprogrammed to acquire the ability to differentiate into various cell types, including germ cells. In the last decade, researchers have successfully reproduced *in vitro* the entire process of oogenesis, and also spermatogenesis, starting from pluripotent stem cells of mice (mPSCs). Briefly, mPSCs were first induced to obtain mouse primordial germ cell-like cells (mPGCLCs). The mPGCLCs were further differentiated into oogonia, which were then induced to enter meiosis and produce functional oocytes, either through *in vivo* transplantation or through *in vitro* culture. In particular, the second method takes advantage of the generation of cell aggregates, defined reconstituted ovaries (rOvaries), obtained by mixing the culture of mPGCLCs with somatic cells from mouse embryonic ovaries, which provide structural and hormonal support for the development of mature and functional oocytes. Moreover, efforts have been made to promote the growth and differentiation of mPGCLCs independently of ovarian somatic cells. The resultant oocytes were fully competent and after *in vitro* fertilization, produced live and fertile offspring (428, 429). The translation of the approach used in mouse IVG has been attempted to induce human PGCLCs (hPGCLCs) from hPSCs, pursuing various methods. The process of hPGCLC specification has been elucidated, revealing the involvement of essential transcription factors, driving specific hierarchical actions, and unique regulatory networks, different from those governing mPGCLC specification. Through culturing hPGCLC with mouse embryonic ovarian somatic cells, defined xenogeneic rOvaries (xrOvaries), hPGCLCs went through epigenetic reprogramming and differentiated through still unknown mechanisms into early oocytes, thus establishing the framework for human IVG (430). IGV provides a new tool for studying mammalian reproduction and prefigures applications in the field of reproductive medicine, enhancing diagnosis and modeling infertility (431). The application of human IVG for reproductive purposes is a possibility, it could be used to produce healthy gametes from patient- or donor-derived iPSCs and used for *in vitro* fertilization or assisted reproduction. This could be beneficial for couples unable to conceive due to fertility issues, genetic diseases, or other conditions. However, the actual low efficiency of the technique, the need of a source of cells equivalent to embryonic gonadal somatic cells in humans and other species, the genetic and epigenetic quality of iPSCs and the resultant gametes, and the risks of genetic abnormalities or diseases in offspring are challenges that researchers are required to solve in coming years (432). Nevertheless, IVG retains great potential for future applications, but inevitably implicates ethical and social considerations.

## 6 Conclusion

POI is a heterogeneous disorder, which can be acquired or congenital, although most of the cases remain idiopathic. It is a common cause of infertility in women in which X chromosome and autoimmune abnormalities play a prevalent pathogenic role. POI has a multisystem impact with profound physical and emotional implications; as such, its optimal management should be handled by a multidisciplinary team. Once it occurs, POI is relatively easily diagnosed since it is characterized clinically by persistent amenorrhea with typical symptoms and hormonally by a hypergonadotropic hypogonadism. Nonetheless, the real challenges are the certification of the underlying ethological cause and the identification of markers that can allow an early diagnosis or, even more important, its prediction. Patients with POI need HRT to restore physiological estrogen levels of the different tissues and organs, in line with patients’ age, and thus avoid the consequences of an early and prolonged hypoestrogenism. While therapeutic approaches in this respect are quite well consolidated, fertility preservation is still a matter of frontier and although recent advancement, hopefully in the future years we will assist novel and important perspectives and options. Moreover, it is critical to improve the educational health resources to raise women’s awareness, health related behaviors and informed decision making, bridging the gap between scientific knowledge and health care provided. The use of the internet and social media, correctly managed by the competent professionals, could be instrumental for this purpose (433).

## Glossary

AARS2: ALANYL-tRNA SYNTHETASE 2;
LIN28A: LIN28 HOMOLOG A
ACBD5: ACYL-CoA-BINDING DOMAIN-CONTAINING PROTEIN 5
LMNA: LAMIN A
AMH: ANTI-MÜLLERIAN HORMONE
MCM8/MCM9: MINICHROMOSOME MAINTENANCE COMPLEX COMPONENT 8/9
AMHR2: ANTI-MULLERIAN HORMONE TYPE II RECEPTOR
MEI4: MEIOTIC DOUBLE-STRANDED BREAK FORMATION PROTEIN 4
ANKRD31: ANKYRIN REPEAT DOMAIN-CONTAINING PROTEIN 31
MEIOB: MEIOSIS-SPECIFIC PROTEIN WITH OB DOMAINS
ANTXR1: ANTHRAX TOXIN RECEPTOR 1
MEIOSIN: MEIOSIS INITIATOR
AR: STEROID HORMONE RECEPTOR FOR ANDROGENS
MGME1: MITOCHONDRIAL GENOME MAINTENANCE EXONUCLEASE 1
ATG7: AUTOPHAGY-RELATED 7
MND1: MEIOTIC NUCLEAR DIVISIONS 1
ATM: ATAXIA-TELANGIECTASIA MUTATED GENE, ATM SERINE/THREONINE KINASE
MRE11: MRE11 HOMOLOG, DOUBLE-STRAND BREAK REPAIR NUCLEASE
BLM: BLOOM SYNDROME
MRPL50: MITOCHONDRIAL RIBOSOMAL PROTEIN L50
BMP15: BONE MORPHOGENETIC PROTEIN 15
MRPS22: MITOCHONDRIAL RIBOSOMAL PROTEIN S22;
BMPR1A/ALK3: BONE MORPHOGENETIC PROTEIN RECEPTOR, TYPE IA
MRPS7: MITOCHONDRIAL RIBOSOMAL PROTEIN S7
BMPR1B/ALK6: BONE MORPHOGENETIC PROTEIN RECEPTOR, TYPE IB
MSH4/MSH5: MutS HOMOLOG 4/5
BMPR2: BONE MORPHOGENETIC PROTEIN RECEPTOR, TYPE II
NANOS3: NANOS C2HC-TYPE ZINC FINGER 3
BRCA1/2: BREAST CANCER DNA REPAIR-ASSOCIATED PROTEIN
NBS1/NBN: NIMBRIN
CLPP: CASEINOLYTIC MITOCHONDRIAL MATRIX PEPTIDASE PROTEOLYTIC SUBUNIT
NOBOX: NEWBORN OVARY HOMEOBOX
CXXC1: CXXC FINGER PROTEIN 1
NOG: NOGGIN
CYP17A1: CYTOCHROME P450, FAMILY 17, SUBFAMILY A, POLYPEPTIDE 1
NOTCH2: NOTCH RECEPTOR 2
CYP19A1: CYTOCHROME P450, FAMILY 19, SUBFAMILY A, POLYPEPTIDE 1
NR5A1: NUCLEAR RECEPTOR SUBFAMILY 5, GROUP A, MEMBER 1
DAZL: DELETED IN AZOOSPERMIA-LIKE
PGRMC1: Progesterone receptor membrane component I
DCAF17: DDB1- AND CUL4-ASSOCIATED FACTOR 17
PMM2: PHOSPHOMANNOMUTASE 2
DMC1: DNA MEIOTIC RECOMBINASE 1
POLG: DNA POLYMERASE GAMMA
EIF2B2; EIF2B4; EIF2B5: EUKARYOTIC TRANSLATION INITIATION FACTOR 2B, SUBUNIT 2, 4 or 5
POLR3H: RNA POLYMERASE III, SUBUNIT H
ERAL1: ERA G-PROTEIN-LIKE 1
PRDM9: PR DOMAIN-CONTAINING PROTEIN 9
ERCC6: ERCC EXCISION REPAIR 6
PREPL: PROLYL ENDOPEPTIDASE-LIKE
ESR1/NR3A1: STEROID HORMONE RECEPTOR FOR ESTROGENS
PRORP: PROTEIN ONLY RNASE P CATALYTIC SUBUNIT
EXO1: EXONUCLEASE 1
RAD21L: RAD21 COHESIN COMPLEX COMPONENT-LIKE 1
FANCA; FANCC; FANCM; FANCD1; FANCU; XRCC9/FANCG: FANCONI ANEMIA, COMPLEMENTATION GROUP A; C; M; D; U; G
RAD50/RAD51: RAD50 DOUBLE-STRAND BREAK REPAIR PROTEIN/RAD51 RECOMBINASE
FIGLA: FOLLICULOGENESIS-SPECIFIC bHLH TRANSCRIPTION FACTOR
RCBTB1: RCC1 DOMAIN- AND BTB DOMAIN-CONTAINING PROTEIN 1
FMR1: FRAGILE X MENTAL RETARDATION 1
REC114: REC114 MEIOTIC RECOMBINATION PROTEIN
FOXL2: FORKHEAD TRANSCRIPTION FACTOR L2
REC8: REC8 MEIOTIC RECOMBINATION PROTEIN
FOXO3: FORKHEAD BOX O3
RECQL4: DNA HELICASE, RECQ-LIKE, TYPE 4
FSHR: FOLLICLE-STIMULATING HORMONE RECEPTOR
RMND1: REQUIRED FOR MEIOTIC NUCLEAR DIVISION 1 HOMOLOG
GALT: GALACTOSE-1-PHOSPHATE URIDYLYLTRANSFERASE
SMC1B: STRUCTURAL MAINTENANCE OF CHROMOSOMES 1B
GATA4: GATA-BINDING PROTEIN 4
SMC3: STRUCTURAL MAINTENANCE OF CHROMOSOMES 3
GDF9: GROWTH/DIFFERENTIATION FACTOR 9
SOHLH1; SOHLH2: OOGENESIS SPECIFIC BASIC HELIX-LOOP-HELIX 1 AND 2
GGPS1: GERANYLGERANYL DIPHOSPHATE SYNTHASE 1
SOX9: SRY-BOX 9
GJA4 connexin37: GAP JUNCTION PROTEIN, ALPHA-4
SPATA22: SPERMATOGENESIS-ASSOCIATED PROTEIN 22
GNAS: GUANINE NUCLEOTIDE-BINDING PROTEIN
SPO11: SPO11 INITIATOR OF MEIOTIC DOUBLE-STRANDED BREAKS
HARS2: HISTIDYL-tRNA SYNTHETASE 2
SRY: SEX REVERSAL 1
HELLS: HELICASE, LYMPHOID-SPECIFIC
STAG3: STROMAL ANTIGEN 3
HFM1: HELICASE FOR MEIOSIS 1
STAR: STEROIDOGENIC ACUTE REGULATORY PROTEIN
HOP2/PSMC3IP: PSMC3 Interacting Protein
STRA8: STIMULATED BY RETINOIC ACID 8
HORMAD1: HORMA DOMAIN-CONTAINING PROTEIN 1
SYCE1: SYNAPTONEMAL COMPLEX CENTRAL ELEMENT PROTEIN 1
HSD17B4: 17-BETA-HYDROXYSTEROID DEHYDROGENASE IV
SYCP1/SYCP2/SYCP3: SYNAPTONEMAL COMPLEX PROTEIN 1/2/3
IHO1: INTERACTOR OF HORMAD1 1
TOPOVIB-Like: TOPOISOMERASE VI-B LIKE
INHA: INHIBIN ALPHA
TP63: TUMOR PROTEIN p63
INSL3: INSULIN-LIKE 3
TWNK: TWINKLE mtDNA HELICASE
LARS2: LEUCYL-tRNA SYNTHETASE 2
WRN: WERNER SYNDROME
LHCGR: LUTEINIZING HORMONE/CHORIOGONADOTROPIN RECEPTOR
WT1: WILMS TUMOR TRANSCRIPTION FACTOR 1
LHX8: LIM homeobox 8
XRCC4: X-RAY REPAIR CROSS COMPLEMENTING 4

## References

[1] Nelson LM. Clinical practice. Primary ovarian insufficiency. N Engl J Med. (2009) 360:606–14. doi: 10.1056/NEJMcp0808697 PMC2762081 19196677

[2] Stuenkel CA Gompel A. Primary ovarian insufficiency. N Engl J Med. (2023) 388:154–63. doi: 10.1056/NEJMcp2116488 36630623

[3] Welt CK. Primary ovarian insufficiency: a more accurate term for premature ovarian failure. Clin Endocrinol (Oxf). (2008) 68:499–509. doi: 10.1111/j.1365-2265.2007.03073.x 17970776

[4] Huhtaniemi I Hovatta O La Marca A Livera G Monniaux D Persani L. Advances in the molecular pathophysiology, genetics, and treatment of primary ovarian insufficiency. Trends Endocrinol Metab. (2018) 29:400–19. doi: 10.1016/j.tem.2018.03.010 29706485

[5] Rossetti R Ferrari I Bonomi M Persani L. Genetics of primary ovarian insufficiency. Clin Genet. (2017) 91:183–98. doi: 10.1111/cge.12921 27861765

[6] Panay N Anderson RA Nappi RE Vincent AJ Vujovic S Webber L. Premature ovarian insufficiency: an International Menopause Society White Paper. Climacteric: J Int Menopause Society. (2020) 23:426–46. doi: 10.1080/13697137.2020.1804547 32896176

[7] Groff AA Covington SN Halverson LR Fitzgerald OR Vanderhoof V Calis K. Assessing the emotional needs of women with spontaneous premature ovarian failure. Fertil Steril. (2005) 83(6):1734–41. doi: 10.1016/j.fertnstert.2004.11.067 15950644

[8] Gruber N Kugler S de Vries L Segev-Becker A Shoenfeld Y Pinhas-Hamiel O. Primary ovarian insufficiency nationwide incidence rate and etiology among Israeli adolescents. J Adolesc Health. (2020) 66:603–9. doi: 10.1016/j.jadohealth.2019.11.315 31987720

[9] Silvén H Savukoski SM Pesonen P. Incidence and familial risk of premature ovarian insufficiency in the Finnish female population. Hum Reprod. (2022) 37):1030–6. doi: 10.1093/humrep/deac014 PMC9071220 35134918

[10] Podfigurna-Stopa A Czyzyk A Grymowicz M Smolarczyk R Katulski K Czajkowski K. Premature ovarian insufficiency: the context of long-term effects. J Endocrinol Invest. (2016) 39:983–90. doi: 10.1007/s40618-016-0467-z PMC4987394 27091671

[11] Tao X Zuo A Wang J Tao F. Effect of primary ovarian insufficiency and early natural menopause on mortality: a meta-analysis. Climacteric. (2015) 19:27–36. doi: 10.3109/13697137.2015.1094784 26576012

[12] Luborsky JL Meyer P Sowers MF Gold EB Santoro. N. Premature menopause in a multi-ethnic population study of the menopause transition. Hum Reproduction. (2003) 18:199–206. doi: 10.1093/humrep/deg005 12525467

[13] Golezar S Ramezani Tehrani F Khazaei S Ebadi A Keshavarz Z. The global prevalence of primary ovarian insufficiency and early menopause: a meta-analysis. Climacteric: J Int Menopause Society. (2019) 22:403–11. doi: 10.1080/13697137.2019.1574738 30829083

[14] Coulam CB Adamson SC Annegers. JF. Incidence of premature ovarian failure. Obstet Gynecol Surv. (1986) 67:604–6. 3960433

[15] Lagergren K Hammar M Nedstrand E Bladh M Sydsjö G. The prevalence of primary ovarian insufficiency in Sweden; a national register study. BMC Womens Health. (2018) 18:175. doi: 10.1186/s12905-018-0665-2 30359245 PMC6202813

[16] Rostami Dovom M Bidhendi-Yarandi R Mohammad K Farahmand M Azizi F Ramezani Tehrani F. Prevalence of premature ovarian insufficiency and its determinants in Iranian populations: Tehran lipid and glucose study. BMC Womens Health. (2021) 21:79. doi: 10.1186/s12905-021-01228-1 33622308 PMC7903639

[17] Verrilli L Johnstone E Welt C Allen-Brady K. Primary ovarian insufficiency has strong familiality: results of a multigenerational genealogical study. Fertil Steril. (2023) 119:128–34. doi: 10.1016/j.fertnstert.2022.09.027 PMC10024920 36283864

[18] Vegetti W Grazia Tibiletti M Testa G Yankowski DL Alagna F Castoldi E. Inheritance in idiopathic premature ovarian failure: analysis of 71 cases. Hum Reprod. (1998) 13:1796–800. doi: 10.1093/humrep/13.7.1796 9740426

[19] Stolk L Perry JR Mangino M Barbalic M Broer L Ernst F. Meta-analyses identify 13 loci associated with age at menopause and highlight DNA repair and immune pathways. Nat Genet. (2012) 44:260–8. doi: 10.1038/ng.1051 PMC3288642 22267201

[20] Tibiletti MG Testa G Vegetti W Alagna F Taborelli M Dalprà L. The idiopathic forms of premature menopause and early menopause show the same genetic pattern. Hum Reprod. (1999) 14:2731–4. doi: 10.1093/humrep/14.11.2731 10548611

[21] Mishra GD Davies MC Hillman S Chung H Roy S Maclaran K. Optimising health after early menopause. Lancet (British edition). (2024) 403:958–68. doi: 10.1016/S0140-6736(23)02800-3 38458215

[22] Ogata T Matsuo N. Turner syndrome and female sex chromosome aberrations: deduction of the principal factors involved in the development of clinical features. Hum Genet. (1995) 95:607–29. doi: 10.1007/BF00209476 7789944

[23] Hook EB Warburton D. Turner syndrome revisited: review of new data supports the hypothesis that all viable 45,X cases are cryptic mosaics with a rescue cell line, implying an origin by mitotic loss. Hum Genet. (2014) 133:417–24. doi: 10.1007/s00439-014-1420-x 24477775

[24] Fechner PY Davenport ML Qualy RL Ross JL Gunther DF Eugster EA. Differences in follicle-stimulating hormone secretion between 45,X monosomy turner syndrome and 45,X/46,XX mosaicism are evident at an early age. J Clin Endocrinol Metab. (2006) 91:4896–902. doi: 10.1210/jc.2006-1157 16968797

[25] Otter M Schrander-Stumpel CTRM Curfs LMG. Triple X syndrome: a review of the literature. Eur J Hum Genet. (2010) 18:265–71. doi: 10.1038/ejhg.2009.109 PMC2987225 19568271

[26] Rafique M AlObaid S Al-Jaroudi D. 47, XXX syndrome with infertility, premature ovarian insufficiency, and streak ovaries. Clin Case Rep. (2019) 7:1238–41. doi: 10.1002/ccr3.2207 PMC6552943 31183102

[27] Chen M Jiang H Zhang C. Selected genetic factors associated with primary ovarian insufficiency. Int J Mol Sci. (2023) 24:4423. doi: 10.3390/ijms24054423 36901862 PMC10002966

[28] Di-Battista A Favilla BP Zamariolli M Nunes N Defelicibus A Armelin-Correa L. Premature ovarian insufficiency is associated with global alterations in the regulatory landscape and gene expression in balanced X-autosome translocations. Epigenet chromatin. (2023) 16:19. doi: 10.1186/s13072-023-00493-8 PMC10197467 37202802

[29] Toniolo D. X-linked premature ovarian failure: a complex disease. Curr Opin Genet Dev. (2006) 16:293–300. doi: 10.1016/j.gde.2006.04.005 16650756

[30] Mercer CL Lachlan K Karcanias A Affara N Huang S Jacobs PA. Detailed clinical and molecular study of 20 females with Xq deletions with special reference to menstruation and fertility. Eur J Med Genet. (2013) 56:1–6. doi: 10.1016/j.ejmg.2012.08.012 23059468

[31] Meyer KF Freitas Filho LG Silva KI Trauzcinsky PA Reuter C Souza MBM. The XY female and SWYER syndrome. Urol Case Rep. (2019) 26:100939. doi: 10.1016/j.eucr.2019.100939 31275808 PMC6586948

[32] Mantovani G Spada A. Mutations in the Gs alpha gene causing hormone resistance. Best Pract Res Clin Endocrinol Metab. (2006) 20:501–13. doi: 10.1016/j.beem.2006.09.001 17161328

[33] Jüppner H. Molecular definition of pseudohypoparathyroidism variants. J Clin Endocrinol Metab. (2021) 106:1541–52. doi: 10.1210/clinem/dgab060 PMC8118362 33529330

[34] Jüppner H. Pseudohypoparathyroidism: complex disease variants with unfortunate names. J Mol Endocrinol. (2024) 72:e230104. doi: 10.1530/JME-23-0104 37965945 PMC10843601

[35] Thiele S Werner R Grötzinger J Brix B Staedt P Struve D. A positive genotype-phenotype correlation in a large cohort of patients with Pseudohypoparathyroidism Type Ia and Pseudo-pseudohypoparathyroidism and 33 newly identified mutations in the GNAS gene. Mol Genet Genomic Med. (2015) 3:111–20. doi: 10.1002/mgg3.117 PMC4367083 25802881

[36] Long XD Xiong J Mo ZH Dong CS Jin P. Identification of a novel GNAS mutation in a case of pseudohypoparathyroidism type 1A with normocalcemia. BMC Med Genet. (2018) 19:132. doi: 10.1186/s12881-018-0648-z 30060753 PMC6065144

[37] Tucker EJ. The genetics and biology of FOXL2. Sex Dev. (2022) 16:184–93. doi: 10.1159/000519836 34727551

[38] Nallathambi J Moumné L De Baere E Beysen D Usha K Sundaresan P. A novel polyalanine expansion in FOXL2: the first evidence for a recessive form of the blepharophimosis syndrome (BPES) associated with ovarian dysfunction. Hum Genet. (2007) 121:107–12. doi: 10.1007/s00439-006-0276-0 17089161

[39] Kaur I Hussain A Naik MN Murthy R Honavar SG. Mutation spectrum of fork-head transcriptional factor gene (FOXL2) in Indian Blepharophimosis Ptosis Epicanthus Inversus Syndrome (BPES) patients. Br J Ophthalmol. (2011) 95:881–6. doi: 10.1136/bjo.2009.177972 21325395

[40] Georges A Auguste A Bessière L Vanet A Todeschini AL Veitia RA. FOXL2: a central transcription factor of the ovary. J Mol Endocrinol. (2014) 52:17. doi: 10.1530/JME-13-0159 24049064

[41] Crisponi L Deiana M Loi A Chiappe F Uda M Amati P. The putative forkhead transcription factor FOXL2 is mutated in blepharophimosis/ptosis/epicanthus inversus syndrome. Nat Genet. (2001) 27:159–66. doi: 10.1038/84781 11175783

[42] Veitia RA. FOXL2 versus SOX9: a lifelong "battle of the sexes. Bioessays. (2010) 32:375–80. doi: 10.1002/bies.200900193 20414895

[43] Tucker EJ Rius R Jaillard S Bell K Lamont PJ Travessa A. Genomic sequencing highlights the diverse molecular causes of Perrault syndrome: a peroxisomal disorder (PEX6), metabolic disorders (CLPP, GGPS1), and mtDNA maintenance/translation disorders (LARS2, TFAM). Hum Genet. (2020) 139:1325–43. doi: 10.1007/s00439-020-02176-w 32399598

[44] Morino H Pierce SB Matsuda Y Walsh T Ohsawa R Newby M. Mutations in Twinkle primase-helicase cause Perrault syndrome with neurologic features. Neurology. (2014) 83:2054–61. doi: 10.1212/WNL.0000000000001036 PMC4248451 25355836

[45] Pierce SB Gersak K Michaelson-Cohen R Walsh T Lee MK Malach D. Mutations in LARS2, encoding mitochondrial leucyl-tRNA synthetase, lead to premature ovarian failure and hearing loss in Perrault syndrome. Am J Hum Genet. (2013) 92:614–20. doi: 10.1016/j.ajhg.2013.03.007 PMC3617377 23541342

[46] Pierce SB Chisholm KM Lynch ED Lee MK Walsh T Opitz JM. Mutations in mitochondrial histidyl tRNA synthetase HARS2 cause ovarian dysgenesis and sensorineural hearing loss of Perrault syndrome. Proc Natl Acad Sci U S A. (2011) 108:6543–8. doi: 10.1073/pnas.1103471108 PMC3081023 21464306

[47] Faridi R Rea A Fenollar-Ferrer C O'Keefe RT Gu S Munir Z. New insights into Perrault syndrome, a clinically and genetically heterogeneous disorder. Hum Genet. (2022) 141:805–19. doi: 10.1007/s00439-021-02319-7 PMC11330641 34338890

[48] Kline BL Jaillard S Bell KM Bakhshalizadeh S Robevska G van den Bergen J. Integral role of the mitochondrial ribosome in supporting ovarian function: MRPS7 variants in syndromic premature ovarian insufficiency. Genes (Basel). (2022) 13:2113. doi: 10.3390/genes13112113 36421788 PMC9690861

[49] Bakhshalizadeh S Hock DH Siddall NA Kline BL Sreenivasan R Bell KM. Deficiency of the mitochondrial ribosomal subunit, MRPL50, causes autosomal recessive syndromic premature ovarian insufficiency. Hum Genet. (2023) 142:879–907. doi: 10.1007/s00439-023-02563-z 37148394 PMC10329598

[50] Luoma P Melberg A Rinne JO Kaukonen JA Nupponen NN Chalmers RM. Parkinsonism, premature menopause, and mitochondrial DNA polymerase gamma mutations: clinical and molecular genetic study. Lancet. (2004) 364:875–82. doi: 10.1016/S0140-6736(04)16983-3 15351195

[51] Filosto M Mancuso M Nishigaki Y Pancrudo J Harati Y Gooch C. Clinical and genetic heterogeneity in progressive external ophthalmoplegia due to mutations in polymerase gamma. Arch Neurol. (2003) 60:1279–84. doi: 10.1001/archneur.60.9.1279 12975295

[52] Chen B Li L Wang J Zhou Y Zhu J Li T. Identification of the first homozygous POLG mutation causing non-syndromic ovarian dysfunction. Climacteric. (2018) 21:467–71. doi: 10.1080/13697137.2018.1467891 29992832

[53] França MM Mendonca BB. Genetics of ovarian insufficiency and defects of folliculogenesis. Best Pract Res Clin Endocrinol Metab. (2022) 36:101594. doi: 10.1016/j.beem.2021.101594 34794894

[54] Fogli A Gauthier-Barichard F Schiffmann R Vanderhoof VH Bakalov VK Nelson LM. Screening for known mutations in EIF2B genes in a large panel of patients with premature ovarian failure. BMC Womens Health. (2004) 4:8. doi: 10.1186/1472-6874-4-8 15507143 PMC529454

[55] Kiraly-Borri C Jevon G Ji W Jeffries L Ricciardi JL Konstantino M. Siblings with lethal primary pulmonary hypoplasia and compound heterozygous variants in the *AARS2* gene: further delineation of the phenotypic spectrum. Cold Spring Harb Mol Case Stud. (2019) 5:a003699. doi: 10.1101/mcs.a003699 30819764 PMC6549552

[56] Ferrer I. The primary microglial leukodystrophies: A review. Int J Mol Sci. (2022) 23:6341. doi: 10.3390/ijms23116341 35683020 PMC9181167

[57] Zhou M Shi N Zheng J Chen Y Wang S Xiao K. Case report: A chinese family of woodhouse-sakati syndrome with diabetes mellitus, with a novel biallelic deletion mutation of the DCAF17 gene. Front Endocrinol (Lausanne). (2021) 12:770871. doi: 10.3389/fendo.2021.770871 35002959 PMC8734028

[58] Yang Q Hua R Qian J Yi S Shen F Zhang Q. PREPL deficiency: A homozygous splice site PREPL mutation in a patient with congenital myasthenic syndrome and absence of ovaries and hypoplasia of uterus. Front Genet. (2020) 11:198. doi: 10.3389/fgene.2020.00198 32218803 PMC7078161

[59] Qin Y Guo T Li G Tang TS Zhao S Jiao X. CSB-PGBD3 mutations cause premature ovarian failure. PLoS Genet. (2015) 11:e1005419. doi: 10.1371/journal.pgen.1005419 26218421 PMC4517778

[60] de Bruin C Mericq V Andrew SF van Duyvenvoorde HA Verkaik NS Losekoot M. An XRCC4 splice mutation associated with severe short stature, gonadal failure, and early-onset metabolic syndrome. J Clin Endocrinol Metab. (2015) 100:789. doi: 10.1210/jc.2015-1098 PMC4422886 25742519

[61] Tucker EJ Grover SR Robevska G van den Bergen J Hanna C Sinclair AH. Identification of variants in pleiotropic genes causing "isolated" premature ovarian insufficiency: implications for medical practice. Eur J Hum Genet. (2018) 26:1319–28. doi: 10.1038/s41431-018-0140-4 PMC6117257 29706645

[62] Szeliga A Zysnarska A Szklarska Z Truszkowska E Podfigurna A Czyzyk A. A case of premature ovarian insufficiency in Nijmegen breakage syndrome patient and review of literature. From gene mutation to clinical management. Gynecol Endocrinol. (2019) 35:999–1002. doi: 10.1080/09513590.2019.1626366 31187634

[63] Turan V Oktay K. BRCA-related ATM-mediated DNA double-strand break repair and ovarian aging. Hum Reprod Update. (2020) 26:43–57. doi: 10.1093/humupd/dmz043 31822904 PMC6935693

[64] Coppieters F Ascari G Dannhausen K Nikopoulos K Peelman F Karlstetter M. Isolated and syndromic retinal dystrophy caused by biallelic mutations in RCBTB1, a gene implicated in ubiquitination. Am J Hum Genet. (2016) 99:470–80. doi: 10.1016/j.ajhg.2016.06.017 PMC4974088 27486781

[65] Rudaks LI Triplett J Morris K Reddel S Worgan L. ACBD5-related retinal dystrophy with leukodystrophy due to novel mutations in ACBD5 and with additional features including ovarian insufficiency. Am J Med Genet A. (2024) 194:346–50. doi: 10.1002/ajmg.a.63433 37789430

[66] Bouman A van Koningsbruggen S Karakullukcu MB Schreuder WH Lakeman P. Bloom syndrome does not always present with sun-sensitive facial erythema. Eur J Med Genet. (2018) 61:94–7. doi: 10.1016/j.ejmg.2017.10.010 29056561

[67] Lebel M Monnat RJ. Werner syndrome (WRN) gene variants and their association with altered function and age-associated diseases. Ageing Res Rev. (2018) 41:82–97. doi: 10.1016/j.arr.2017.11.003 29146545

[68] Gonzalo S Kreienkamp R Askjaer P. Hutchinson-Gilford Progeria Syndrome: A premature aging disease caused by LMNA gene mutations. Ageing Res Rev. (2017) 33:18–29. doi: 10.1016/j.arr.2016.06.007 27374873 PMC5195863

[69] Siitonen HA Sotkasiira J Biervliet M Benmansour A Capri Y Cormier-Daire V. The mutation spectrum in RECQL4 diseases. Eur J Hum Genet. (2009) 17:151–8. doi: 10.1038/ejhg.2008.154 PMC2986053 18716613

[70] Demirhan O Türkmen S Schwabe GC Soyupak S Akgül E Tastemir D. A homozygous BMPR1B mutation causes a new subtype of acromesomelic chondrodysplasia with genital anomalies. J Med Genet. (2005) 42:314–7. doi: 10.1136/jmg.2004.023564 PMC1736042 15805157

[71] Kosaki K Sato S Hasegawa T Matsuo N Suzuki T Ogata T. Premature ovarian failure in a female with proximal symphalangism and Noggin mutation. Fertil Steril. (2004) 81:1137–9. doi: 10.1016/j.fertnstert.2003.08.054 15066478

[72] Benetti-Pinto C Ferreira V Andrade L Yela DA De Mello MP. GAPO syndrome: a new syndromic cause of premature ovarian insufficiency. Climacteric. (2016) 19:594–8. doi: 10.1080/13697137.2016.1200551 27426988

[73] Stránecký V Hoischen A Hartmannová H Zaki MS Chaudhary A Zudaire E. Mutations in ANTXR1 cause GAPO syndrome. Am J Hum Genet. (2013) 92:792–9. doi: 10.1016/j.ajhg.2013.03.023 PMC3644626 23602711

[74] Zhao J Zhang Y Li W Yao M Liu C Zhang Z. Research progress of the Fanconi anemia pathway and premature ovarian insufficiency†. Biol Reprod. (2023) 109:570–85. doi: 10.1093/biolre/ioad110 37669135

[75] Weinberg-Shukron A Rachmiel M Renbaum P Gulsuner S Walsh T Lobel O. Essential role of BRCA2 in ovarian development and function. N Engl J Med. (2018) 379:1042–9. doi: 10.1056/NEJMoa1800024 PMC6230262 30207912

[76] Bakkar AA Alsaedi A Kamal NM Althobaiti E Aboulkhair LA Almalki AM. Lipoid congenital adrenal hyperplasia with a novel stAR gene mutation. Clin Med Insights Endocrinol Diabetes. (2023) 16:11795514231167059. doi: 10.1177/11795514231167059 37255966 PMC10226314

[77] Nazari M Yahya Vahidi Mehrjardi M Neghab N Aghabagheri M Ghasemi N. A novel mutation in CYP17A1 gene leads to congenital adrenal hyperplasia: A case report. Int J Reprod Biomed. (2019) 17:449–54. doi: 10.18502/ijrm.v17i6.4817 PMC6719517 31508570

[78] Dursun F Ceylaner S. A novel homozygous CYP19A1 gene mutation: aromatase deficiency mimicking congenital adrenal hyperplasia in an infant without obvious maternal virilisation. J Clin Res Pediatr Endocrinol. (2019) 11:196–201. doi: 10.4274/jcrpe 30074481 PMC6571529

[79] Masunaga Y Mochizuki M Kadoya M Wada Y Okamoto N Fukami M. Primary ovarian insufficiency in a female with phosphomannomutase-2 gene (PMM2) mutations for congenital disorder of glycosylation. Endocr J. (2021) 68:605–11. doi: 10.1507/endocrj.EJ20-0706 33583911

[80] Derks B Rivera-Cruz G Hagen-Lillevik S Vos EN Demirbas D Lai K. The hypergonadotropic hypogonadism conundrum of classic galactosemia. Hum Reprod Update. (2023) 29:246–58. doi: 10.1093/humupd/dmac041 PMC9976963 36512573

[81] Luo W Ke H Tang S Jiao X Li Z Zhao S. Next-generation sequencing of 500 POI patients identified novel responsible monogenic and oligogenic variants. J Ovarian Res. (2023) 16:39. doi: 10.1186/s13048-023-01104-6 36793102 PMC9930292

[82] Chon SJ Umair Z Yoon MS. Premature ovarian insufficiency: past, present, and future. Front Cell Dev Biol. (2021) 9:672890. doi: 10.3389/fcell.2021.672890 34041247 PMC8141617

[83] Wang Y Guo T Ke H Zhang Q Li S Luo W. Pathogenic variants of meiotic double strand break (DSB) formation genes PRDM9 and ANKRD31 in premature ovarian insufficiency. Genet Med. (2021) 23:2309–15. doi: 10.1038/s41436-021-01266-y PMC8629753 34257419

[84] Persani L Rossetti R Cacciatore C. Genes involved in human premature ovarian failure. J Mol Endocrinol. (2010) 45:257–79. doi: 10.1677/JME-10-0070 20668067

[85] Renault L Patiño LC Magnin F Delemer B Young J Laissue P. BMPR1A and BMPR1B missense mutations cause primary ovarian insufficiency. J Clin Endocrinol Metab. (2020) 105:dgz226. doi: 10.1210/clinem/dgz226 31769494

[86] Patiño LC Silgado D Laissue P. A potential functional association between mutant BMPR2 and primary ovarian insufficiency. Syst Biol Reprod Med. (2017) 63:145–9. doi: 10.1080/19396368.2017.1291767 28306340

[87] Mandon-Pépin B Touraine P Kuttenn F Derbois C Rouxel A Matsuda F. Genetic investigation of four meiotic genes in women with premature ovarian failure. Eur J Endocrinol. (2008) 158:107–15. doi: 10.1530/EJE-07-0400 18166824

[88] Cao D Shi F Guo C Liu Y Lin Z Zhang J. A pathogenic DMC1 frameshift mutation causes nonobstructive azoospermia but not primary ovarian insufficiency in humans. Mol Hum Reprod. (2021) 27:gaab058. doi: 10.1093/molehr/gaab058 34515795

[89] M'Rabet N Moffat R Helbling S Kaech A Zhang H de Geyter C. The CC-allele of the PvuII polymorphic variant in intron 1 of the α-estrogen receptor gene is significantly more prevalent among infertile women at risk of premature ovarian aging. Fertil Steril. (2012) 98:965–5. doi: 10.1016/j.fertnstert.2012.05.048 22749220

[90] Liu L Tan R Cui Y Liu J Wu J. Estrogen receptor α gene (ESR1) polymorphisms associated with idiopathic premature ovarian failure in Chinese women. Gynecol Endocrinol. (2013) 29:182–5. doi: 10.3109/09513590.2012.731113 23116284

[91] Cattoni A Spano A Tulone A Boneschi A Masera N Maitz S. The potential synergic effect of a complex pattern of multiple inherited genetic variants as a pathogenic factor for ovarian dysgenesis: A case report. Front Endocrinol (Lausanne). (2020) 11:540683. doi: 10.3389/fendo.2020.540683 33101191 PMC7545356

[92] Chen B Li L Wang J Li T Pan H Liu B. Consanguineous familial study revealed biallelic FIGLA mutation associated with premature ovarian insufficiency. J Ovarian Res. (2018) 11:48. doi: 10.1186/s13048-018-0413-0 29914564 PMC6006558

[93] Yuan P He Z Sun S Li Y Wang W Liang X. Bi-allelic recessive loss-of-function mutations in FIGLA cause premature ovarian insufficiency with short stature. Clin Genet. (2019) 95:409–14. doi: 10.1111/cge.13486 30474133

[94] Er E Aşıkovalı S Özışık H Sağsak E GÖkşen D Onay H. Investigation of the molecular genetic causes of non-syndromic primary ovarian ınsufficiency by next generation sequencing analysis. Arch Endocrinol Metab. (2023) 68:e220475. doi: 10.20945/2359-4292-2022-0475 37988663 PMC10916837

[95] Wang Z Liu CY Zhao Y Dean J. FIGLA, LHX8 and SOHLH1 transcription factor networks regulate mouse oocyte growth and differentiation. Nucleic Acids Res. (2020) 48:3525–41. doi: 10.1093/nar/gkaa101 PMC7144910 32086523

[96] Rodari G Federici S Todisco T Ubertini G Cattoni A Pagano M. Towards an individualized management of pubertal induction in girls with hypogonadism: insight into the best replacement outcomes from a large multicentre registry. Eur J endocrinology. (2023) 188:467–76. doi: 10.1093/ejendo/lvad056 37232247

[97] Wittenberger MD Hagerman RJ Sherman SL McConkie-Rosell A Welt CK Rebar RW. The FMR1 premutation and reproduction. Fertility sterility. (2007) 87:456–65. doi: 10.1016/j.fertnstert.2006.09.004 17074338

[98] Watkins WJ Umbers AJ Woad KJ Harris SE Winship IM Gersak K. Mutational screening of FOXO3A and FOXO1A in women with premature ovarian failure. Fertil Steril. (2006) 86:1518–21. doi: 10.1016/j.fertnstert.2006.03.054 16979636

[99] Patiño LC Beau I Carlosama C Buitrago JC González R Suárez CF. New mutations in non-syndromic primary ovarian insufficiency patients identified via whole-exome sequencing. Hum Reprod. (2017) 32:1512–20. doi: 10.1093/humrep/dex089 28505269

[100] Liu H Wei X Sha Y Liu W Gao H Lin J. Whole-exome sequencing in patients with premature ovarian insufficiency: early detection and early intervention. J Ovarian Res. (2020) 13:114. doi: 10.1186/s13048-020-00716-6 32962729 PMC7510158

[101] Zeng Y Li L Li Q Hu J Zhang N Wu L. Genetic screening in patients with ovarian dysfunction. Clin Genet. (2023) 103:352–7. doi: 10.1111/cge.14267 36373164

[102] Jin H Ahn J Park Y Sim J Park HS Ryu CS. Identification of potential causal variants for premature ovarian failure by whole exome sequencing. BMC Med Genomics. (2020) 13:159. doi: 10.1186/s12920-020-00813-x 33109206 PMC7590468

[103] Pu D Wang C Cao J Shen Y Jiang H Liu J. Association analysis between HFM1 variation and primary ovarian insufficiency in Chinese women. Clin Genet. (2016) 89:597–602. doi: 10.1111/cge.12718 26679638

[104] Tucker EJ Bell KM Robevska G van den Bergen J Ayers KL Listyasari N. Meiotic genes in premature ovarian insufficiency: variants in HROB and REC8 as likely genetic causes. Eur J Hum Genet. (2022) 30:219–28. doi: 10.1038/s41431-021-00977-9 PMC8821714 34707299

[105] Eskenazi S Bachelot A Hugon-Rodin J Plu-Bureau G Gompel A Catteau-Jonard S. Next generation sequencing should be proposed to every woman with "Idiopathic" Primary ovarian insufficiency. J Endocr Soc. (2021) 5:bvab032. doi: 10.1210/jendso/bvab032 34095689 PMC8169040

[106] Fonseca DJ Patiño LC Suárez YC de Jesús Rodríguez A Mateus HE Jiménez KM. Next generation sequencing in women affected by nonsyndromic premature ovarian failure displays new potential causative genes and mutations. Fertil Steril. (2015) 104:154,62.e2. doi: 10.1016/j.fertnstert.2015.04.016 25989972

[107] Lu X Yan Z Cai R Khor S Wu L Sun L. Pregnancy and live birth in women with pathogenic LHCGR variants using their own oocytes. J Clin Endocrinol Metab. (2019) 104:5877–92. doi: 10.1210/jc.2019-01276 31393569

[108] Qin Y Zhao H Kovanci E Simpson JL Chen ZJ Rajkovic A. Analysis of LHX8 mutation in premature ovarian failure. Fertil Steril. (2008) 89:1012–4. doi: 10.1016/j.fertnstert.2007.04.017 PMC2680741 17624344

[109] Zhao L Li Q Kuang Y Xu P Sun X Meng Q. Heterozygous loss-of-function variants in LHX8 cause female infertility characterized by oocyte maturation arrest. Genet Med. (2022) 24:2274–84. doi: 10.1016/j.gim.2022.07.027 36029299

[110] Helderman NC Terlouw D Bonjoch L Golubicki M Antelo M Morreau H. Molecular functions of MCM8 and MCM9 and their associated pathologies. iScience. (2023) 26:106737. doi: 10.1016/j.isci.2023.106737 37378315 PMC10291252

[111] Desai S Wood-Trageser M Matic J Chipkin J Jiang H Bachelot A. MCM8 and MCM9 nucleotide variants in women with primary ovarian insufficiency. J Clin Endocrinol Metab. (2017) 102:576–82. doi: 10.1210/jc.2016-2565 PMC5413161 27802094

[112] Dou X Guo T Li G Zhou L Qin Y Chen ZJ. Minichromosome maintenance complex component 8 mutations cause primary ovarian insufficiency. Fertil Steril. (2016) 106:1485,1489.e2. doi: 10.1016/j.fertnstert.2016.08.018 27573988

[113] França MM Funari MFA Lerario AM Santos MG Nishi MY Domenice S. Screening of targeted panel genes in Brazilian patients with primary ovarian insufficiency. PLoS One. (2020) 15:e0240795. doi: 10.1371/journal.pone.0240795 33095795 PMC7584253

[114] Alvarez-Mora M Todeschini AL Caburet S Perets LP Mila M Younis JS. An exome-wide exploration of cases of primary ovarian insufficiency uncovers novel sequence variants and candidate genes. Clin Genet. (2020) 98:293–8. doi: 10.1111/cge.13803 32613604

[115] Guo T Zheng Y Li G Zhao S Ma J Qin Y. Novel pathogenic mutations in minichromosome maintenance complex component 9 (MCM9) responsible for premature ovarian insufficiency. Fertil Steril. (2020) 113:845–52. doi: 10.1016/j.fertnstert.2019.11.015 32145932

[116] Shen J Qu D Gao Y Sun F Xie J Sun X. Genetic etiologic analysis in 74 Chinese Han women with idiopathic premature ovarian insufficiency by combined molecular genetic testing. J Assist Reprod Genet. (2021) 38:965–78. doi: 10.1007/s10815-021-02083-7 PMC8079602 33538981

[117] Caburet S Todeschini AL Petrillo C Martini E Farran ND Legois B. A truncating MEIOB mutation responsible for familial primary ovarian insufficiency abolishes its interaction with its partner SPATA22 and their recruitment to DNA double-strand breaks. EBioMedicine. (2019) 42:524–31. doi: 10.1016/j.ebiom.2019.03.075 PMC6491878 31000419

[118] Wang Y Liu L Tan C Meng G Meng L Nie H. Novel MEIOB variants cause primary ovarian insufficiency and non-obstructive azoospermia. Front Genet. (2022) 13:936264. doi: 10.3389/fgene.2022.936264 35991565 PMC9388730

[119] Zhang Q Zhang W Wu X Ke H Qin Y Zhao S. Homozygous missense variant in MEIOSIN causes premature ovarian insufficiency. Hum Reprod. (2023) 38:ii47–56. doi: 10.1093/humrep/dead084 37982418

[120] Jolly A Bayram Y Turan S Aycan Z Tos T Abali ZY. Exome sequencing of a primary ovarian insufficiency cohort reveals common molecular etiologies for a spectrum of disease. J Clin Endocrinol Metab. (2019) 104:3049–67. doi: 10.1210/jc.2019-00248 PMC6563799 31042289

[121] Chen A Tiosano D Guran T Baris HN Bayram Y Mory A. Mutations in the mitochondrial ribosomal protein MRPS22 lead to primary ovarian insufficiency. Hum Mol Genet. (2018) 27:1913–26. doi: 10.1093/hmg/ddy098 PMC5961111 29566152

[122] Wan Y Hong Z Ma B He X Ma L Wang M. Identification of compound heterozygous variants in MSH4 as a novel genetic cause of diminished ovarian reserve. Reprod Biol Endocrinol. (2023) 21:76. doi: 10.1186/s12958-023-01127-0 37620942 PMC10464148

[123] Guo T Zhao S Chen M Li G Jiao X Wang Z. Mutations in MSH5 in primary ovarian insufficiency. Hum Mol Genet. (2017) 26:1452–7. doi: 10.1093/hmg/ddx044 PMC5393145 28175301

[124] Wu X Wang B Dong Z Zhou S Liu Z Shi G. A NANOS3 mutation linked to protein degradation causes premature ovarian insufficiency. Cell Death Dis. (2013) 4:e825. doi: 10.1038/cddis.2013.368 24091668 PMC3824677

[125] Santos MG MaChado AZ Martins CN Domenice S Costa EM Nishi MY. Homozygous inactivating mutation in NANOS3 in two sisters with primary ovarian insufficiency. BioMed Res Int. (2014) 2014:787465. doi: 10.1155/2014/787465 25054146 PMC4098983

[126] Jordan P Verebi C Perol S Grotto S Fouveaut C Christin-Maitre S. NOBOX gene variants in premature ovarian insufficiency: ethnicity-dependent insights. J Assist Reprod Genet. (2024) 41:135–46. doi: 10.1007/s10815-023-02981-y PMC10789696 37921973

[127] Ferrari I Bouilly J Beau I Guizzardi F Ferlin A Pollazzon M. Impaired protein stability and nuclear localization of NOBOX variants associated with premature ovarian insufficiency. Hum Mol Genet. (2016) 25:5223–33. doi: 10.1093/hmg/ddw342 27798098

[128] Li L Feng F Zhao M Li T Yue W Ma X. NOTCH2 variant D1853H is mutated in two non-syndromic premature ovarian insufficiency patients from a Chinese pedigree. J Ovarian Res. (2020) 13:41. doi: 10.1186/s13048-020-00645-4 32312275 PMC7171760

[129] Bouilly J Beau I Barraud S Bernard V Azibi K Fagart J. Identification of multiple gene mutations accounts for a new genetic architecture of primary ovarian insufficiency. J Clin Endocrinol Metab. (2016) 101:4541–50. doi: 10.1210/jc.2016-2152 27603904

[130] Bayram Y Gulsuner S Guran T Abaci A Yesil G Gulsuner HU. Homozygous loss-of-function mutations in SOHLH1 in patients with nonsyndromic hypergonadotropic hypogonadism. J Clin Endocrinol Metab. (2015) 100:808. doi: 10.1210/jc.2015-1150 PMC4422898 25774885

[131] Zhao S Li G Dalgleish R Vujovic S Jiao X Li J. Transcription factor SOHLH1 potentially associated with primary ovarian insufficiency. Fertil Steril. (2015) 103:548,53.e5. doi: 10.1016/j.fertnstert.2014.11.011 25527234

[132] Qin Y Jiao X Dalgleish R Vujovic S Li J Simpson JL. Novel variants in the SOHLH2 gene are implicated in human premature ovarian failure. Fertil Steril. (2014) 101:1104,1109.e6. doi: 10.1016/j.fertnstert.2014.01.001 24524832

[133] Demain LAM Boetje E Edgerley JJ Miles E Fitzgerald CT Busby G. Biallelic loss of function variants in STAG3 result in primary ovarian insufficiency. Reprod BioMed Online. (2021) 43:899–902. doi: 10.1016/j.rbmo.2021.07.003 34497033

[134] Mellone S Zavattaro M Vurchio D Ronzani S Caputo M Leone I. A long contiguous stretch of homozygosity disclosed a novel STAG3 biallelic pathogenic variant causing primary ovarian insufficiency: A case report and review of the literature. Genes. (2021) 12:1709. doi: 10.3390/genes12111709 34828315 PMC8622734

[135] Akbari A Zoha Tabatabaei S Salehi N Padidar K Almadani N Ali Sadighi Gilani M. Novel STAG3 variant associated with primary ovarian insufficiency and non-obstructive azoospermia in an Iranian consanguineous family. Gene. (2022) 821:146281. doi: 10.1016/j.gene.2022.146281 35176428

[136] Gómez-Rojas S Aristizábal-Duque JE Muñoz-Fernández LF Sarmiento-Ramón MP Pereira-Gómez MDP. New STAG3 gene variant as a cause of premature ovarian insufficiency. Rev Colomb Obstet Ginecol. (2022) 73:142–8. doi: 10.18597/rcog.3806 PMC9097685 35503298

[137] McGuire MM Bowden W Engel NJ Ahn HW Kovanci E Rajkovic A. Genomic analysis using high-resolution single-nucleotide polymorphism arrays reveals novel microdeletions associated with premature ovarian failure. Fertil Steril. (2011) 95:1595–600. doi: 10.1016/j.fertnstert.2010.12.052 PMC3062633 21256485

[138] de Vries L Behar DM Smirin-Yosef P Lagovsky I Tzur S Basel-Vanagaite L. Exome sequencing reveals SYCE1 mutation associated with autosomal recessive primary ovarian insufficiency. J Clin Endocrinol Metab. (2014) 99:2129. doi: 10.1210/jc.2014-1268 25062452

[139] Hou D Yao C Xu B Luo W Ke H Li Z. Variations of C14ORF39 and SYCE1 identified in idiopathic premature ovarian insufficiency and nonobstructive azoospermia. J Clin Endocrinol Metab. (2022) 107:724–34. doi: 10.1210/clinem/dgab777 34718620

[140] Bestetti I Castronovo C Sironi A Caslini C Sala C Rossetti R. High-resolution array-CGH analysis on 46,XX patients affected by early onset primary ovarian insufficiency discloses new genes involved in ovarian function. Hum Reprod. (2019) 34:574–83. doi: 10.1093/humrep/dey389 PMC6389867 30689869

[141] Tucker EJ Jaillard S Grover SR van den Bergen J Robevska G Bell KM. TP63-truncating variants cause isolated premature ovarian insufficiency. Hum Mutat. (2019) 40:886–92. doi: 10.1002/humu.23744 30924587

[142] Huang C Zhao S Yang Y Guo T Ke H Mi X. TP63 gain-of-function mutations cause premature ovarian insufficiency by inducing oocyte apoptosis. J Clin Invest. (2023) 133:e162315. doi: 10.1172/JCI162315 36856110 PMC9974095

[143] Vanderschelden RK Rodriguez-Escriba M Chan SH Berman AJ Rajkovic A Yatsenko SA. Heterozygous TP63 pathogenic variants in isolated primary ovarian insufficiency. J Assist Reprod Genet. (2023) 40:2211–8. doi: 10.1007/s10815-023-02886-w PMC10440319 37453019

[144] Panda B Rao L Tosh D Dixit H Padmalatha V Kanakavalli M. Germline study ofARgene of Indian women with ovarian failure. Gynecological Endocrinology. (2010) 27:572–8. doi: 10.3109/09513590.2010.507282 20672904

[145] Wang ET Pisarska MD Bresee C Chen YD Lester J Afshar Y. BRCA1 germline mutations may be associated with reduced ovarian reserve. Fertility sterility. (2014) 102:1723–8. doi: 10.1016/j.fertnstert.2014.08.014 PMC4372188 25256924

[146] Luo W Guo T Li G Liu R Zhao S Song M. Variants in homologous recombination genes EXO1 and RAD51 related with premature ovarian insufficiency. J Clin Endocrinol Metab. (2020) 105:dgaa505. doi: 10.1210/clinem/dgaa505 32772095

[147] Bachelot A Gilleron J Meduri G Guberto M Dulon J Boucherie S. A common African variant of human connexin 37 is associated with Caucasian primary ovarian insufficiency and has a deleterious effect *in vitro* . Int J Mol Med. (2018) 41:640–8. doi: 10.3892/ijmm.2017.3257 PMC5752242 29207017

[148] Franca MM Han X Funari MFA Lerario AM Nishi MY Fontenele EGP. Exome sequencing reveals the POLR3H gene as a novel cause of primary ovarian insufficiency. J Clin Endocrinol Metab. (2019) 104:2827–41. doi: 10.1210/jc.2018-02485 PMC6543511 30830215

[149] Zangen D Kaufman Y Zeligson S Perlberg S Fridman H Kanaan M. XX ovarian dysgenesis is caused by a PSMC3IP/HOP2 mutation that abolishes coactivation of estrogen-driven transcription. Am J Hum Genet. (2011) 89:572–9. doi: 10.1016/j.ajhg.2011.09.006 PMC3188834 21963259

[150] Sirchia F Giorgio E Cucinella L Valente EM Nappi RE. Biallelic mutations in PSMC3IP are associated with secondary amenorrhea: expanding the spectrum of premature ovarian insufficiency. J Assist Reprod Genet. (2022) 39:1177–81. doi: 10.1007/s10815-022-02471-7 PMC9107541 35352317

[151] Yao C Hou D Ji Z Pang D Li P Tian R. Bi-allelic SPATA22 variants cause premature ovarian insufficiency and nonobstructive azoospermia due to meiotic arrest. Clin Genet. (2022) 101:507–16. doi: 10.1111/cge.14129 35285020

[152] Zhou Z Yin H Suye S Ren Z Yan L Shi L. Fance deficiency inhibits primordial germ cell proliferation associated with transcription-replication conflicts accumulate and DNA repair defects. J Ovarian Res. (2023) 16:160. doi: 10.1186/s13048-023-01252-9 37563658 PMC10416540

[153] El-Khairi R Parnaik R Duncan AJ Lin L Gerrelli D Dattani MT. Analysis of LIN28A in early human ovary development and as a candidate gene for primary ovarian insufficiency. Mol Cell Endocrinol. (2012) 351:264–8. doi: 10.1016/j.mce.2011.12.016 PMC3314903 22240064

[154] Song ZH Yu HY Wang P Mao GK Liu WX Li MN. Germ cell-specific Atg7 knockout results in primary ovarian insufficiency in female mice. Cell Death Dis. (2015) 6:e1589. doi: 10.1038/cddis.2014.559 25590799 PMC4669757

[155] Viswanathan SR Daley GQ Gregory RI. Selective blockade of microRNA processing by Lin28. Science. (2008) 320:97–100. doi: 10.1126/science.1154040 18292307 PMC3368499

[156] Zhu H Shah S Shyh-Chang N Shinoda G Einhorn WS Viswanathan SR. Lin28a transgenic mice manifest size and puberty phenotypes identified in human genetic association studies. Nat Genet. (2010) 42:626–30. doi: 10.1038/ng.593 PMC3069638 20512147

[157] Soyal SM Amleh A Dean J. FIGalpha, a germ cell-specific transcription factor required for ovarian follicle formation. Development. (2000) 127:4645–54. doi: 10.1242/dev.127.21.4645 11023867

[158] Rosario R Filis P Tessyman V Kinnell H Childs AJ Gray NK. FMRP associates with cytoplasmic granules at the onset of meiosis in the human oocyte. PLoS One. (2016) 11:e0163987. doi: 10.1371/journal.pone.0163987 27695106 PMC5047637

[159] Ishiguro KI Matsuura K Tani N Takeda N Usuki S Yamane M. MEIOSIN directs the switch from mitosis to meiosis in mammalian germ cells. Dev Cell. (2020) 52:429,445.e10. doi: 10.1016/j.devcel.2020.01.010 32032549

[160] Zhang T He M Zhang J Tong Y Chen T Wang C. Mechanisms of primordial follicle activation and new pregnancy opportunity for premature ovarian failure patients. Front Physiol. (2023) 14. doi: 10.3389/fphys.2023.1113684 PMC10011087 36926197

[161] Coxir SA Costa GMJ Santos CFD Alvarenga RLLS Lacerda SMDS. From *in vivo* to *in vitro*: exploring the key molecular and cellular aspects of human female gametogenesis. Hum Cell. (2023) 36:1283–311. doi: 10.1007/s13577-023-00921-7 37237248

[162] Reynolds N Collier B Bingham V Gray NK Cooke HJ. Translation of the synaptonemal complex component Sycp3 is enhanced *in vivo* by the germ cell specific regulator Dazl. RNA. (2007) 13:974–81. doi: 10.1261/rna.465507 PMC1894923 17526644

[163] Tung JY Rosen MP Nelson LM Turek PJ Witte JS Cramer DW. Novel missense mutations of the Deleted-in-AZoospermia-Like (DAZL) gene in infertile women and men. Reprod Biol Endocrinol. (2006) 4:40. doi: 10.1186/1477-7827-4-40 16884537 PMC1557510

[164] França MM Mendonca BB. Genetics of primary ovarian insufficiency in the next-generation sequencing era. J Endocr Soc. (2020) 4:bvz037. doi: 10.1210/jendso/bvz037 32099950 PMC7033037

[165] Huang C Guo T Qin Y. Meiotic recombination defects and premature ovarian insufficiency. Front Cell Dev Biol. (2021) 9:652407. doi: 10.3389/fcell.2021.652407 33763429 PMC7982532

[166] Okutman O Boivin M Muller J Charlet-Berguerand N Viville S. A biallelic loss of function variant in HORMAD1 within a large consanguineous Turkish family is associated with spermatogenic arrest. Hum Reprod. (2023) 38:306–14. doi: 10.1093/humrep/deac259 36524333

[167] Dong J Zhang H Mao X Zhu J Li D Fu J. Novel biallelic mutations in MEI1: expanding the phenotypic spectrum to human embryonic arrest and recurrent implantation failure. Hum Reprod. (2021) 36:2371–81. doi: 10.1093/humrep/deab118 34037756

[168] Pan Z Wang W Wu L Yao Z Chen Y Gu H. Bi-allelic missense variants in MEI4 cause preimplantation embryonic arrest and female infertility. Hum Genet. (2024). doi: 10.1007/s00439-023-02633-2 38252283

[169] Anand R Jasrotia A Bundschuh D Howard SM Ranjha L Stucki M. NBS1 promotes the endonuclease activity of the MRE11-RAD50 complex by sensing CtIP phosphorylation. EMBO J. (2019) 38:e101005. doi: 10.15252/embj.2018101005 30787182 PMC6443204

[170] Kang J Bronson RT Xu Y. Targeted disruption of NBS1 reveals its roles in mouse development and DNA repair. EMBO J. (2002) 21:1447–55. doi: 10.1093/emboj/21.6.1447 PMC125926 11889050

[171] Inagaki A Roset R Petrini JH. Functions of the MRE11 complex in the development and maintenance of oocytes. Chromosoma. (2016) 125:151–62. doi: 10.1007/s00412-015-0535-8 PMC4734907 26232174

[172] Warcoin M Lespinasse J Despouy G Dubois d'Enghien C Laugé A Portnoï MF. Fertility defects revealing germline biallelic nonsense NBN mutations. Hum Mutat. (2009) 30:424–30. doi: 10.1002/humu.20904 19105185

[173] Wei K Clark AB Wong E Kane MF Mazur DJ Parris T. Inactivation of Exonuclease 1 in mice results in DNA mismatch repair defects, increased cancer susceptibility, and male and female sterility. Genes Dev. (2003) 17:603–14. doi: 10.1101/gad.1060603 PMC196005 12629043

[174] He WB Tu CF Liu Q Meng LL Yuan SM Luo AX. mutation that causes human non-obstructive azoospermia and premature ovarian insufficiency identified by whole-exome sequencing. J Med Genet. (2018) 55:198–204. doi: 10.1136/jmedgenet-2017-104992 29331980

[175] Janisiw E Dello Stritto MR Jantsch V Silva N. BRCA1-BARD1 associate with the synaptonemal complex and pro-crossover factors and influence RAD-51 dynamics during Caenorhabditis elegans meiosis. PLoS Genet. (2018) 14:e1007653. doi: 10.1371/journal.pgen.1007653 30383754 PMC6211622

[176] Lord CJ Ashworth A. BRCAness revisited. Nat Rev Cancer. (2016) 16:110–20. doi: 10.1038/nrc.2015.21 26775620

[177] Miao Y Wang P Xie B Yang M Li S Cui Z. BRCA2 deficiency is a potential driver for human primary ovarian insufficiency. Cell Death Dis. (2019) 10:474. doi: 10.1038/s41419-019-1720-0 31209201 PMC6572856

[178] Caburet S Heddar A Dardillac E Creux H Lambert M Messiaen S. Homozygous hypomorphic BRCA2 variant in primary ovarian insufficiency without cancer or Fanconi anaemia trait. J Med Genet. (2021) 58:125–34. doi: 10.1136/jmedgenet-2019-106672 32482800

[179] Qin Y Zhang F Chen ZJ. BRCA2 in ovarian development and function. N Engl J Med. (2019) 380:1086. doi: 10.1056/NEJMc1813800 30865812

[180] Titus S Li F Stobezki R Akula K Unsal E Jeong K. Impairment of BRCA1-related DNA double-strand break repair leads to ovarian aging in mice and humans. Sci Transl Med. (2013) 5:172ra21. doi: 10.1126/scitranslmed.3004925 PMC5130338 23408054

[181] Borgogno MV Monti MR Zhao W Sung P Argaraña CE Pezza RJ. Tolerance of DNA mismatches in dmc1 recombinase-mediated DNA strand exchange. J Biol Chem. (2016) 291:4928–38. doi: 10.1074/jbc.M115.704718 PMC4777831 26709229

[182] Sansam CL Pezza RJ. Connecting by breaking and repairing: mechanisms of DNA strand exchange in meiotic recombination. FEBS J. (2015) 282:2444–57. doi: 10.1111/febs.13317 PMC4573575 25953379

[183] de la Fuente R Pratto F Hernández-Hernández A Manterola M López-Jiménez P Gómez R. Epigenetic dysregulation of mammalian male meiosis caused by interference of recombination and synapsis. Cells. (2021) 10:2311. doi: 10.3390/cells10092311 34571960 PMC8467405

[184] Xu Y Greenberg RA Schonbrunn E Wang PJ. Meiosis-specific proteins MEIOB and SPATA22 cooperatively associate with the single-stranded DNA-binding replication protein A complex and DNA double-strand breaks. Biol Reprod. (2017) 96:1096–104. doi: 10.1093/biolre/iox040 PMC6355104 28453612

[185] Snowden T Shim KS Schmutte C Acharya S Fishel R. hMSH4-hMSH5 adenosine nucleotide processing and interactions with homologous recombination machinery. J Biol Chem. (2008) 283:145–54. doi: 10.1074/jbc.M704060200 PMC2841433 17977839

[186] de Vries SS Baart EB Dekker M Siezen A de Rooij DG de Boer P. Mouse MutS-like protein Msh5 is required for proper chromosome synapsis in male and female meiosis. Genes Dev. (1999) 13:523–31. doi: 10.1101/gad.13.5.523 PMC316502 10072381

[187] Kneitz B Cohen PE Avdievich E Zhu L Kane MF Hou H. MutS homolog 4 localization to meiotic chromosomes is required for chromosome pairing during meiosis in male and female mice. Genes Dev. (2000) 14:1085–97. doi: 10.1101/gad.14.9.1085 PMC316572 10809667

[188] Nishimura K Ishiai M Horikawa K Fukagawa T Takata M Takisawa H. Mcm8 and Mcm9 form a complex that functions in homologous recombination repair induced by DNA interstrand crosslinks. Mol Cell. (2012) 47:511–22. doi: 10.1016/j.molcel.2012.05.047 22771115

[189] Lutzmann M Grey C Traver S Ganier O Maya-Mendoza A Ranisavljevic N. MCM8- and MCM9-deficient mice reveal gametogenesis defects and genome instability due to impaired homologous recombination. Mol Cell. (2012) 47:523–34. doi: 10.1016/j.molcel.2012.05.048 22771120

[190] Goldberg Y Aleme O Peled-Perets L Castellvi-Bel S Nielsen M Shalev SA. MCM9 is associated with germline predisposition to early-onset cancer-clinical evidence. NPJ Genom Med. (2021) 6:78. doi: 10.1038/s41525-021-00242-4 34556653 PMC8460657

[191] Zhe J Ye D Chen X Liu Y Zhou X Li Y. Consanguineous chinese familial study reveals that a gross deletion that includes the SYCE1 gene region is associated with premature ovarian insufficiency. Reprod Sci. (2020) 27:461–7. doi: 10.1007/s43032-019-00037-0 31925770

[192] Hernández-López D Geisinger A Trovero MF Santiñaque FF Brauer M Folle GA. Familial primary ovarian insufficiency associated with an SYCE1 point mutation: defective meiosis elucidated in humanized mice. Mol Hum Reprod. (2020) 26:485–97. doi: 10.1093/molehr/gaaa032 32402064

[193] Ishiguro KI. The cohesin complex in mammalian meiosis. Genes Cells. (2019) 24:6–30. doi: 10.1111/gtc.12652 30479058 PMC7379579

[194] Caburet S Arboleda VA Llano E Overbeek PA Barbero JL Oka K. Mutant cohesin in premature ovarian failure. N Engl J Med. (2014) 370:943–9. doi: 10.1056/NEJMoa1309635 PMC4068824 24597867

[195] Revenkova E Eijpe M Heyting C Hodges CA Hunt PA Liebe B. Cohesin SMC1 beta is required for meiotic chromosome dynamics, sister chromatid cohesion and DNA recombination. Nat Cell Biol. (2004) 6:555–62. doi: 10.1038/ncb1135 15146193

[196] Xu H Beasley MD Warren WD van der Horst GT McKay MJ. Absence of mouse REC8 cohesin promotes synapsis of sister chromatids in meiosis. Dev Cell. (2005) 8:949–61. doi: 10.1016/j.devcel.2005.03.018 15935783

[197] Suh EK Yang A Kettenbach A Bamberger C Michaelis AH Zhu Z. p63 protects the female germ line during meiotic arrest. Nature. (2006) 444:624–8. doi: 10.1038/nature05337 17122775

[198] Lena AM Rossi V Osterburg S Smirnov A Osterburg C Tuppi M. The p63 C-terminus is essential for murine oocyte integrity. Nat Commun. (2021) 12:383. doi: 10.1038/s41467-020-20669-0 33452256 PMC7810856

[199] Tucker EJ Gutfreund N Belaud-Rotureau M Gilot D Brun T Kline BL. Dominant TP63 missense variants lead to constitutive activation and premature ovarian insufficiency. Hum Mutat. (2022) 43:1443–53. doi: 10.1002/humu.24432 PMC9542062 35801529

[200] McGee EA Hsueh AJ. Initial and cyclic recruitment of ovarian follicles. Endocr Rev. (2000) 21:200–14. doi: 10.1210/edrv.21.2.0394 10782364

[201] Liu YX Zhang Y Li YY Liu XM Wang XX Zhang CL. Regulation of follicular development and differentiation by intra-ovarian factors and endocrine hormones. Front Biosci (Landmark Ed). (2019) 24:983–93. doi: 10.2741/4763 30844725

[202] La Marca A Mastellari E. Fertility preservation for genetic diseases leading to premature ovarian insufficiency (POI). J Assist Reprod Genet. (2021) 38:759–77. doi: 10.1007/s10815-021-02067-7 PMC8079553 33495935

[203] Ford EA Beckett EL Roman SD McLaughlin EA Sutherland JM. Advances in human primordial follicle activation and premature ovarian insufficiency. Reproduction. (2020) 159:R15–29. doi: 10.1530/REP-19-0201 31376814

[204] Maidarti M Anderson RA Telfer EE. Crosstalk between PTEN/PI3K/akt signalling and DNA damage in the oocyte: implications for primordial follicle activation, oocyte quality and ageing. Cells. (2020) 9:200. doi: 10.3390/cells9010200 31947601 PMC7016612

[205] Rajkovic A Pangas SA Ballow D Suzumori N Matzuk MM. NOBOX deficiency disrupts early folliculogenesis and oocyte-specific gene expression. Science. (2004) 305:1157–9. doi: 10.1126/science.1099755 15326356

[206] Rodríguez-Escribà M Rodríguez-Alonso B Belur S Rajkovic A. Sohlh1 loss of function male and female infertility model impacts overall health beyond gonadal dysfunction in mice†. Biol Reprod. (2023) 108:619–28. doi: 10.1093/biolre/ioad008 PMC10106844 36723967

[207] Choi Y Ballow DJ Xin Y Rajkovic A. Lim homeobox gene, lhx8, is essential for mouse oocyte differentiation and survival. Biol Reprod. (2008) 79:442–9. doi: 10.1095/biolreprod.108.069393 PMC2710541 18509161

[208] Jagarlamudi K Rajkovic A. Oogenesis: transcriptional regulators and mouse models. Mol Cell Endocrinol. (2012) 356:31–9. doi: 10.1016/j.mce.2011.07.049 21856374

[209] Liu L Rajareddy S Reddy P Du C Jagarlamudi K Shen Y. Infertility caused by retardation of follicular development in mice with oocyte-specific expression of Foxo3a. Development. (2007) 134:199–209. doi: 10.1242/dev.02667 17164425

[210] Bouilly J Bachelot A Broutin I Touraine P Binart N. Novel NOBOX loss-of-function mutations account for 6.2% of cases in a large primary ovarian insufficiency cohort. Hum Mutat. (2011) 32:1108–13. doi: 10.1002/humu.21543 21837770

[211] Gallardo TD John GB Bradshaw K Welt C Reijo-Pera R Vogt PH. Sequence variation at the human FOXO3 locus: a study of premature ovarian failure and primary amenorrhea. Hum Reprod. (2008) 23:216–21. doi: 10.1093/humrep/dem255 PMC2579771 17959613

[212] Cecconi S Ciccarelli C Barberi M Macchiarelli G Canipari R. Granulosa cell-oocyte interactions. Eur J Obstet Gynecol Reprod Biol. (2004) 115 Suppl 1:19. doi: 10.1016/j.ejogrb.2004.01.010 15196711

[213] Wassarman PM Litscher ES. Female fertility and the zona pellucida. Elife. (2022) 11:e76106. doi: 10.7554/eLife.76106 PMC8789258 35076396

[214] Gao EM Turathum B Wang L Zhang D Liu YB Tang RX. The differential metabolomes in cumulus and mural granulosa cells from human preovulatory follicles. Reprod Sci. (2022) 29:1343–56. doi: 10.1007/s43032-021-00691-3 PMC8907092 34374964

[215] Dompe C Kulus M Stefańska K Kranc W Chermuła B Bryl R. Human granulosa cells-stemness properties, molecular cross-talk and follicular angiogenesis. Cells. (2021) 10:1396. doi: 10.3390/cells10061396 34198768 PMC8229878

[216] Uhlenhaut NH Jakob S Anlag K Eisenberger T Sekido R Kress J. Somatic sex reprogramming of adult ovaries to testes by FOXL2 ablation. Cell. (2009) 139:1130–42. doi: 10.1016/j.cell.2009.11.021 20005806

[217] Harris SE Chand AL Winship IM Gersak K Aittomäki K Shelling AN. Identification of novel mutations in FOXL2 associated with premature ovarian failure. Mol Hum Reprod. (2002) 8:729–33. doi: 10.1093/molehr/8.8.729 12149404

[218] Mottershead DG Sugimura S Al-Musawi SL Li J Richani D White MA. Cumulin, an oocyte-secreted heterodimer of the transforming growth factor-β Family, is a potent activator of granulosa cells and improves oocyte quality. J Biol Chem. (2015) 290:24007–20. doi: 10.1074/jbc.M115.671487 PMC4583026 26254468

[219] Di Pasquale E Beck-Peccoz P Persani L. Hypergonadotropic ovarian failure associated with an inherited mutation of human bone morphogenetic protein-15 ( BMP15) gene. Am J Hum Genet. (2004) 75:106–11. doi: 10.1086/422103 PMC1181993 15136966

[220] Di Pasquale E Rossetti R Marozzi A Bodega B Borgato S Cavallo L. Identification of new variants of human BMP15 gene in a large cohort of women with premature ovarian failure. J Clin Endocrinol Metab. (2006) 91:1976–9. doi: 10.1210/jc.2005-2650 16464940

[221] Dixit H Rao LK Padmalatha VV Kanakavalli M Deenadayal M Gupta N. Missense mutations in the BMP15 gene are associated with ovarian failure. Hum Genet. (2006) 119:408–15. doi: 10.1007/s00439-006-0150-0 16508750

[222] Laissue P Christin-Maitre S Touraine P Kuttenn F Ritvos O Aittomaki K. Mutations and sequence variants in GDF9 and BMP15 in patients with premature ovarian failure. Eur J Endocrinol. (2006) 154:739–44. doi: 10.1530/eje.1.02135 16645022

[223] Rossetti R Di Pasquale E Marozzi A Bione S Toniolo D Grammatico P. BMP15 mutations associated with primary ovarian insufficiency cause a defective production of bioactive protein. Hum mutation. (2009) 30:804–10. doi: 10.1002/humu.v30:5 PMC2677132 19263482

[224] Tiotiu D Alvaro Mercadal B Imbert R Verbist J Demeestere I De Leener A. Variants of the BMP15 gene in a cohort of patients with premature ovarian failure. Hum Reprod. (2010) 25:1581–7. doi: 10.1093/humrep/deq073 20364024

[225] Wang B Wen Q Ni F Zhou S Wang J Cao Y. Analyses of growth differentiation factor 9 (GDF9) and bone morphogenetic protein 15 (BMP15) mutation in Chinese women with premature ovarian failure. Clin Endocrinol (Oxford). (2010) 72:135–6. doi: 10.1111/j.1365-2265.2009.03613.x 19438907

[226] Rossetti R Ferrari I Bestetti I Moleri S Brancati F Petrone L. Fundamental role of BMP15 in human ovarian folliculogenesis revealed by null and missense mutations associated with primary ovarian insufficiency. Hum mutation. (2020) 41:983–97. doi: 10.1002/humu.23988 31957178

[227] Zinn AR Tonk VS Chen Z Flejter WL Gardner HA Guerra R. Evidence for a turner syndrome locus or loci at xp11.2-p22.1. Am J Hum Genet. (1998) 63:1757–66. doi: 10.1086/302152 PMC1377648 9837829

[228] Persani L Rossetti R Cacciatore C Bonomi M. Primary Ovarian Insufficiency: X chromosome defects and autoimmunity. J Autoimmun. (2009) 33:35–41. doi: 10.1016/j.jaut.2009.03.004 19346101

[229] Dalbies-Tran R Cadoret V Desmarchais A Elis S Maillard V Monget P. A comparative analysis of oocyte development in mammals. Cells. (2020) 9:1002. doi: 10.3390/cells9041002 32316494 PMC7226043

[230] Shimizu K Nakamura T Bayasula Nakanishi N Kasahara Y Nagai T. Molecular mechanism of FSHR expression induced by BMP15 in human granulosa cells. J Assist Reprod Genet. (2019) 36:1185–94. doi: 10.1007/s10815-019-01469-y PMC6603124 31079267

[231] Liu MN Zhang K Xu TM. The role of BMP15 and GDF9 in the pathogenesis of primary ovarian insufficiency. Hum Fertil (Camb). (2021) 24:325–32. doi: 10.1080/14647273.2019.1672107 31607184

[232] Aittomäki K Lucena JL Pakarinen P Sistonen P Tapanainen J Gromoll J. Mutation in the follicle-stimulating hormone receptor gene causes hereditary hypergonadotropic ovarian failure. Cell. (1995) 82:959–68. doi: 10.1016/0092-8674(95)90275-9 7553856

[233] Cimino I Casoni F Liu X Messina A Parkash J Jamin SP. Novel role for anti-Müllerian hormone in the regulation of GnRH neuron excitability and hormone secretion. Nat Commun. (2016) 7:10055. doi: 10.1038/ncomms10055 26753790 PMC4729924

[234] Kevenaar ME Themmen AP Rivadeneira F Uitterlinden AG Laven JS van Schoor NM. A polymorphism in the AMH type II receptor gene is associated with age at menopause in interaction with parity. Hum Reprod. (2007) 22:2382–8. doi: 10.1093/humrep/dem176 17636279

[235] Qin C Yuan Z Yao J Zhu W Wu W Xie J. AMH and AMHR2 genetic variants in Chinese women with primary ovarian insufficiency and normal age at natural menopause. Reprod BioMed Online. (2014) 29:311–8. doi: 10.1016/j.rbmo.2014.05.003 24912417

[236] Li L Zhou X Wang X Wang J Zhang W Wang B. A dominant negative mutation at the ATP binding domain of AMHR2 is associated with a defective anti-Müllerian hormone signaling pathway. Mol Hum Reprod. (2016) 22:669–78. doi: 10.1093/molehr/gaw040 27430550

[237] Alvaro Mercadal B Imbert R Demeestere I Gervy C De Leener A Englert Y. AMH mutations with reduced *in vitro* bioactivity are related to premature ovarian insufficiency. Hum Reprod. (2015) 30:1196–202. doi: 10.1093/humrep/dev042 25750103

[238] Marozzi A Porta C Vegetti W Crosignani PG Tibiletti MG Dalprà L. Mutation analysis of the inhibin alpha gene in a cohort of Italian women affected by ovarian failure. Hum Reprod. (2002) 17:1741–5. doi: 10.1093/humrep/17.7.1741 12093833

[239] Macklon NS Fauser BC. Follicle-stimulating hormone and advanced follicle development in the human. Arch Med Res. (2001) 32:595–600. doi: 10.1016/S0188-4409(01)00327-7 11750735

[240] Latronico AC Anasti J Arnhold IJ Rapaport R Mendonca BB Bloise W. Brief report: testicular and ovarian resistance to luteinizing hormone caused by inactivating mutations of the luteinizing hormone-receptor gene. N Engl J Med. (1996) 334:507–12. doi: 10.1056/NEJM199602223340805 8559204

[241] Rao CV Lei ZM. Consequences of targeted inactivation of LH receptors. Mol Cell Endocrinol. (2002) 187:57–67. doi: 10.1016/S0303-7207(01)00694-3 11988312

[242] Pakarainen T Zhang FP Poutanen M Huhtaniemi I. Fertility in luteinizing hormone receptor-knockout mice after wild-type ovary transplantation demonstrates redundancy of extragonadal luteinizing hormone action. J Clin Invest. (2005) 115:1862–8. doi: 10.1172/JCI24562 PMC1143591 15951841

[243] Drummond AE Findlay JK. The role of estrogen in folliculogenesis. Mol Cell Endocrinol. (1999) 151:57–64. doi: 10.1016/S0303-7207(99)00038-6 10411320

[244] Cordts EB Santos AA Peluso C Bianco B Barbosa CP Christofolini DM. Risk of premature ovarian failure is associated to the PvuII polymorphism at estrogen receptor gene ESR1. J Assist Reprod Genet. (2012) 29:1421–5. doi: 10.1007/s10815-012-9884-x PMC3528877 23150099

[245] Shiina H Matsumoto T Sato T Igarashi K Miyamoto J Takemasa S. Premature ovarian failure in androgen receptor-deficient mice. Proc Natl Acad Sci U S A. (2006) 103:224–9. doi: 10.1073/pnas.0506736102 PMC1324980 16373508

[246] Venturella R De Vivo V Carlea A D'Alessandro P Saccone G Arduino B. The genetics of non-syndromic primary ovarian insufficiency: A systematic review. Int J Fertil Steril. (2019) 13:161–8. doi: 10.22074/ijfs.2019.5599 PMC6642427 31310068

[247] Clarke HJ. Transzonal projections: Essential structures mediating intercellular communication in the mammalian ovarian follicle. Mol Reprod Dev. (2022) 89:509–25. doi: 10.1002/mrd.23645 36112806

[248] Simon AM Goodenough DA Li E Paul DL. Female infertility in mice lacking connexin 37. Nature. (1997) 385:525–9. doi: 10.1038/385525a0 9020357

[249] Bouilly J Roucher-Boulez F Gompel A Bry-Gauillard H Azibi K Beldjord C. New NOBOX mutations identified in a large cohort of women with primary ovarian insufficiency decrease KIT-L expression. J Clin Endocrinol Metab. (2015) 100:994–1001. doi: 10.1210/jc.2014-2761 25514101

[250] Carlsson IB Laitinen MP Scott JE Louhio H Velentzis L Tuuri T. Kit ligand and c-Kit are expressed during early human ovarian follicular development and their interaction is required for the survival of follicles in long-term culture. Reproduction. (2006) 131:641–9. doi: 10.1530/rep.1.00868 16595715

[251] Zhang CP Yang JL Zhang J Li L Huang L Ji SY. Notch signaling is involved in ovarian follicle development by regulating granulosa cell proliferation. Endocrinology. (2011) 152:2437–47. doi: 10.1210/en.2010-1182 21427220

[252] Hubbard N Prasasya RD Mayo KE. Activation of notch signaling by oocytes and jag1 in mouse ovarian granulosa cells. Endocrinology. (2019) 160:2863–76. doi: 10.1210/en.2019-00564 PMC6850001 31609444

[253] Lv S Sun J. Identification and validation of autophagy-related genes in primary ovarian insufficiency by gene expression profile and bioinformatic analysis. Anal Cell Pathol (Amst). (2022) 2022:9042380. doi: 10.1155/2022/9042380 35837294 PMC9273469

[254] Liu M Wu K Wu Y. The emerging role of ferroptosis in female reproductive disorders. BioMed Pharmacother. (2023) 166:115415. doi: 10.1016/j.biopha.2023.115415 37660655

[255] Pei Z Deng K Xu C Zhang S. The molecular regulatory mechanisms of meiotic arrest and resumption in Oocyte development and maturation. Reprod Biol Endocrinol. (2023) 21:90. doi: 10.1186/s12958-023-01143-0 37784186 PMC10544615

[256] Kanamori M Oikawa K Tanemura K Hara K. Mammalian germ cell migration during development, growth, and homeostasis. Reprod Med Biol. (2019) 18:247–55. doi: 10.1002/rmb2.12283 PMC6613016 31312103

[257] Nouri N Shareghi-Oskoue O Aghebati-Maleki L Danaii S Ahmadian Heris J Soltani-Zangbar M. Role of miRNAs interference on ovarian functions and premature ovarian failure. Cell Commun Signal. (2022) 20:198. doi: 10.1186/s12964-022-00992-3 36564840 PMC9783981

[258] Zhou Q Liu Z Liao Z Zhang Y Qu M Wu F. miRNA profiling of granulosa cell-derived exosomes reveals their role in promoting follicle development. J Cell Physiol. (2024) 239:20–35. doi: 10.1002/jcp.31140 38149730

[259] Telfer EE Grosbois J Odey YL Rosario R Anderson RA. Making a good egg: human oocyte health, aging, and *in vitro* development. Physiol Rev. (2023) 103:2623–77. doi: 10.1152/physrev.00032.2022 PMC10625843 37171807

[260] Mehlmann LM. Stops and starts in mammalian oocytes: recent advances in understanding the regulation of meiotic arrest and oocyte maturation. Reproduction. (2005) 130:791–9. doi: 10.1530/rep.1.00793 16322539

[261] Welt CK Hall JE Adams JM Taylor AE. Relationship of estradiol and inhibin to the follicle-stimulating hormone variability in hypergonadotropic hypogonadism or premature ovarian failure. J Clin Endocrinol Metab. (2005) 90:826–30. doi: 10.1210/jc.2004-1319 15562017

[262] Hubayter ZR Popat V Vanderhoof VH Ndubizu O Johnson D Mao E. A prospective evaluation of antral follicle function in women with 46,XX spontaneous primary ovarian insufficiency. Fertil Steril. (2010) 94:1769–74. doi: 10.1016/j.fertnstert.2009.10.023 PMC2888894 19939372

[263] Nelson LM Anasti JN Kimzey LM Defensor RA Lipetz KJ White BJ. Development of luteinized graafian follicles in patients with karyotypically normal spontaneous premature ovarian failure. J Clin Endocrinol Metab. (1994) 79:1470–5. doi: 10.1210/jcem.79.5.7962345 7962345

[264] Liu W Chen M Liu C Wang L Wei H Zhang R. Epg5 deficiency leads to primary ovarian insufficiency due to WT1 accumulation in mouse granulosa cells. Autophagy. (2023) 19:644–59. doi: 10.1080/15548627.2022.2094671 PMC9851269 35786405

[265] Wang Y Chen Q Zhang F Yang X Shang L Ren S. Whole exome sequencing identified a rare WT1 loss-of-function variant in a non-syndromic POI patient. Mol Genet Genomic Med. (2022) 10:e1820. doi: 10.1002/mgg3.1820 34845858 PMC8801142

[266] Meinsohn MC Smith OE Bertolin K Murphy BD. The orphan nuclear receptors steroidogenic factor-1 and liver receptor homolog-1: structure, regulation, and essential roles in mammalian reproduction. Physiol Rev. (2019) 99:1249–79. doi: 10.1152/physrev.00019.2018 30810078

[267] Ivell R Anand-Ivell R. Insulin-like peptide 3 (INSL3) is a major regulator of female reproductive physiology. Hum Reprod Update. (2018) 24:639–51. doi: 10.1093/humupd/dmy029 30204868

[268] Peluso JJ. Progesterone receptor membrane component 1 and its role in ovarian follicle growth. Front Neurosci. (2013) 7:99. doi: 10.3389/fnins.2013.00099 23781168 PMC3680780

[269] Mansouri MR Schuster J Badhai J Stattin EL Lösel R Wehling M. Alterations in the expression, structure and function of progesterone receptor membrane component-1 (PGRMC1) in premature ovarian failure. Hum Mol Genet. (2008) 17:3776–83. doi: 10.1093/hmg/ddn274 PMC2722898 18782852

[270] Wang JL Li SL Qin YY Chen ZJ. Analysis of progesterone receptor membrane component 1 mutation in Han Chinese women with premature ovarian failure. Reprod BioMed Online. (2014) 29:640–3. doi: 10.1016/j.rbmo.2014.08.001 25246111

[271] Bennett-Toomey J Stocco C. GATA regulation and function during the ovarian life cycle. Vitam Horm. (2018) 107:193–225. doi: 10.1016/bs.vh.2018.01.014 29544631 PMC12057165

[272] Dinh DT Breen J Nicol B Foot NJ Bersten DC Emery A. Progesterone receptor mediates ovulatory transcription through RUNX transcription factor interactions and chromatin remodelling. Nucleic Acids Res. (2023) 51:5981–96. doi: 10.1093/nar/gkad271 PMC10325896 37099375

[273] Pierson Smela MD Kramme CC Fortuna PRJ Adams JL Su R Dong E. Directed differentiation of human iPSCs to functional ovarian granulosa-like cells via transcription factor overexpression. Elife. (2023) 12:e83291. doi: 10.7554/eLife.83291 36803359 PMC9943069

[274] May-Panloup P Chretien M Malthiery Y Reynier P. Mitochondrial DNA in the oocyte and the developing embryo. Curr Topics Dev Biol. (2007) 77:51–83. doi: 10.1016/S0070-2153(06)77003-X 17222700

[275] Wai T Ao A Zhang X Cyr D Dufort D Shoubridge EA. The role of mitochondrial DNA copy number in mammalian fertility. Biol Reprod. (2010) 83:52–62. doi: 10.1095/biolreprod.109.080887 20130269 PMC2888963

[276] Sreerangaraja Urs DB Wu WH Komrskova K Postlerova P Lin YF Tzeng CR. Mitochondrial function in modulating human granulosa cell steroidogenesis and female fertility. Int J Mol Sci. (2020) 21:3592. doi: 10.3390/ijms21103592 32438750 PMC7279321

[277] Pagnamenta AT Taanman J-W Wilson CJ Anderson NE Marotta R Duncan AJ. Dominant inheritance of premature ovarian failure associated with mutant mitochondrial DNA polymerase gamma. Hum Reprod. (2006) 21:2467–73. doi: 10.1093/humrep/del076 16595552

[278] Menezes MJ Guo Y Zhang J Riley LG Cooper ST Thorburn DR. Mutation in mitochondrial ribosomal protein S7 (MRPS7) causes congenital sensorineural deafness, progressive hepatic and renal failure and lactic acidemia. Hum Mol Genet. (2015) 24:2297–307. doi: 10.1093/hmg/ddu747 25556185

[279] Bonomi M Somigliana E Cacciatore C Busnelli M Rossetti R Bonetti S. Blood cell mitochondrial DNA content and premature ovarian aging. PLoS One. (2012) 7:e42423. doi: 10.1371/journal.pone.0042423 22879975 PMC3411770

[280] Busnelli A Lattuada D Rossetti R Paffoni A Persani L Fedele L. Mitochondrial DNA copy number in peripheral blood: a potential non-invasive biomarker for female subfertility. J Assist Reprod Genet. (2018) 35:1987–94. doi: 10.1007/s10815-018-1291-5 PMC6240551 30120634

[281] Bakalov VK Anasti JN Calis KA Vanderhoof VH Premkumar A Chen S. Autoimmune oophoritis as a mechanism of follicular dysfunction in women with 46,XX spontaneous premature ovarian failure. Fertil Steril. (2005) 84:958–65. doi: 10.1016/j.fertnstert.2005.04.060 16213850

[282] Ishizuka B. Current understanding of the etiology, symptomatology, and treatment options in premature ovarian insufficiency (POI). Front Endocrinol. (2021) 12. doi: 10.3389/fendo.2021.626924 PMC7949002 33716979

[283] Vogt EC Real FG Husebye ES Björnsdottir S Benediktsdottir B Bertelsen RJ. Premature menopause and autoimmune primary ovarian insufficiency in two international multi-center cohorts. Endocrine connections. (2022) 11:e220024. doi: 10.1530/EC-22-0024 35521804 PMC9175594

[284] Grossmann B Saur S Rall K Pecher AC Hübner S Henes J. Prevalence of autoimmune disease in women with premature ovarian failure. Eur J contraception Reprod Health care: Off J Eur Soc Contraception. (2020) 25:72–5. doi: 10.1080/13625187.2019.1702638 31852274

[285] Hoek A Schoemaker J Drexhage HA. Premature ovarian failure and ovarian autoimmunity. Endocr Rev. (1997) 18:107–34. doi: 10.1210/edrv.18.1.0291 9034788

[286] Betterle C Volpato M. Adrenal and ovarian autoimmunity. Eur J endocrinology. (1998) 138:16–25. doi: 10.1530/eje.0.1380016 9461309

[287] Falorni A Laureti S Santeusanio F. Autoantibodies in autoimmune polyendocrine syndrome type II. Endocrinol Metab Clin North Am. (2002) 31(2):369–89. doi: 10.1016/S0889-8529(01)00010-X 12092456

[288] Lebovic DI Naz R. Premature ovarian failure: Think ‘autoimmune disorder’. Sexuality Reprod menopause. (2004) 2:230–3. doi: 10.1016/j.sram.2004.11.010

[289] Goswami D Conway GS. Premature ovarian failure. Hum Reprod Update. (2005) 11:391–410. doi: 10.1093/humupd/dmi012 15919682

[290] Dal Pra C Chen S Furmaniak J Smith BR Pedini B Moscon A. Autoantibodies to steroidogenic enzymes in patients with premature ovarian failure with and without Addison's disease. Eur J endocrinology. (2003) 148:565–70. doi: 10.1530/eje.0.1480565 12720541

[291] Yan G Schoenfeld D Penney C Hurxthal K Taylor AE Faustman D. Identification of premature ovarian failure patients with underlying autoimmunity. J Womens Health Gend Based. (2000) 9:275–87. doi: 10.1089/152460900318461 10787223

[292] McLeod DSA Cooper DS. The incidence and prevalence of thyroid autoimmunity. Endocrine. (2012) 42:252–65. doi: 10.1007/s12020-012-9703-2 22644837

[293] Li Z Xu S Luo W Hu J Zhang T Jiao X. Association between thyroid autoimmunity and the decline of ovarian reserve in euthyroid women. Reprod BioMed Online. (2022) 45:615–22. doi: 10.1016/j.rbmo.2022.05.015 35732549

[294] Collins G Patel B Thakore S Liu J. Primary ovarian insufficiency: current concepts. South Med J. (2017) 110:147–53. doi: 10.14423/SMJ.0000000000000611 28257537

[295] Ebrahimi M Firoozeh AA. Pathogenesis and causes of premature ovarian failure: an update. Int J Fertil Steril. (2011) 5:54–65. 24963360 PMC4059950

[296] Schlessinger D Herrera L Crisponi L Mumm S Percesepe A Pellegrini M. Genes and translocations involved in POF. Am J Med Genet. (2002) 111:328–33. doi: 10.1002/ajmg.10565 12210333

[297] Silva CA Yamakami LYS Aikawa NE Araujo DB Carvalho JF Bonfá E. Autoimmune primary ovarian insufficiency. Autoimmun Rev. (2014) 13:427–30. doi: 10.1016/j.autrev.2014.01.003 24418305

[298] Welt CK Falorni A Taylor AE Martin KA Hall JE. Selective theca cell dysfunction in autoimmune oophoritis results in multifollicular development, decreased estradiol, and elevated inhibin B levels. J Clin Endocrinol Metab. (2005) 90:3069–76. doi: 10.1210/jc.2004-1985 15705922

[299] Rudnicka E Kruszewska J Klicka K Kowalczyk J Grymowicz M Skórska J. Premature ovarian insufficiency - aetiopathology, epidemiology, and diagnostic evaluation. Przeglad menopauzalny = Menopause review. (2018) 17:105–8. doi: 10.5114/pm.2018.78550 PMC6196779 30357004

[300] Van Dorp W Mulder RL Kremer LCM Hudson MM Van Den Heuvel-Eibrink MM Van Den Berg MH. Recommendations for premature ovarian insufficiency surveillance for female survivors of childhood, adolescent, and young adult cancer: A report from the international late effects of childhood cancer guideline harmonization group in collaboration with the panCareSurFup consortium. J Clin oncology: Off J Am Soc Clin Oncol. (2016) 34:3440–50. doi: 10.1200/JCO.2015.64.3288 PMC5569686 27458300

[301] Gracia CR Sammel MD Freeman E Prewitt M Carlson C Ray A. Impact of cancer therapies on ovarian reserve. Fertil Steril. (2012) 97:134-40.e1. doi: 10.1016/j.fertnstert.2011.10.040 22137491 PMC4005036

[302] Spears N Lopes F Stefansdottir A Rossi V De Felici M Anderson RA. Ovarian damage from chemotherapy and current approaches to its protection. Hum Reprod Update. (2019) 25:673–93. doi: 10.1093/humupd/dmz027 PMC6847836 31600388

[303] Oktem O Oktay K. Quantitative assessment of the impact of chemotherapy on ovarian follicle reserve and stromal function. Cancer. (2007) 110:2222–9. doi: 10.1002/cncr.23071 17932880

[304] Sklar CA Mertens AC Mitby P Whitton J Stovall M Kasper C. Premature menopause in survivors of childhood cancer: a report from the childhood cancer survivor study. J Natl Cancer Inst. (2006) 98:890–6. doi: 10.1093/jnci/djj243 16818852

[305] Tariq S Anderson J Burns F Delpech V Gilson R Sabin C. The menopause transition in women living with HIV: current evidence and future avenues of research. J Virus Eradication. (2016) 2:114. doi: 10.1016/S2055-6640(20)30476-3 PMC4965243 27482447

[306] Scherzer R Bacchetti P Messerlian G Goderre J Maki PM Seifer DB. Impact of CD4+ lymphocytes and HIV infection on Anti-Müllerian Hormone levels in a large cohort of HIV-infected and HIV-uninfected women. Am J Reprod Immunol (New York N.Y.: 1989). (2015) 73:273–84. doi: 10.1111/aji.12332 PMC4323676 25339186

[307] Vabre P Gatimel N Moreau J Gayrard V Picard-Hagen N Parinaud J. Environmental pollutants, a possible etiology for premature ovarian insufficiency: a narrative review of animal and human data. Environ health: Global Access Sci Source. (2017) 16:37. doi: 10.1186/s12940-017-0242-4 PMC5384040 28388912

[308] Zhu X Liu M Dong R Gao L Hu J Zhang X. Mechanism exploration of environmental pollutants on premature ovarian insufficiency: a systematic review and meta-analysis. Reprod Sci (Thousand Oaks Calif.). (2024) 31:99–106. doi: 10.1007/s43032-023-01326-5 37612521

[309] Webber L Davies M Anderson R Bartlett J Braat D Cartwright B. ESHRE Guideline: management of women with premature ovarian insufficiency. Hum Reprod. (2016) 31:926–37. doi: 10.1093/humrep/dew027 27008889

[310] Bachelot A Nicolas C Bidet M Dulon J Leban M Golmard JL. Long-term outcome of ovarian function in women with intermittent premature ovarian insufficiency. Clin Endocrinology. (2016) 86:223–8. doi: 10.1111/cen.13105 27177971

[311] Bidet M Bachelot A Bissauge E Golmard JL Gricourt S Dulon J. Resumption of ovarian function and pregnancies in 358 patients with premature ovarian failure. J Clin Endocrinol Metab. (2011) 96:3864–72. doi: 10.1210/jc.2011-1038 21994953

[312] Fraison E Crawford G Casper G Harris V Ledger W. Pregnancy following diagnosis of premature ovarian insufficiency: a systematic review. Reprod BioMedicine Online. (2019) 39:467–76. doi: 10.1016/j.rbmo.2019.04.019 31279714

[313] Weenen C Laven JSE Von Bergh ARM Cranfield M Groome NP Visser JA. Anti-Mullerian hormone expression pattern in the human ovary: potential implications for initial and cyclic follicle recruitment. Mol Hum Reprod. (2004) 10:77–83. doi: 10.1093/molehr/gah015 14742691

[314] Jeppesen JV Anderson RA Kelsey TW Christiansen SL Jayaprakasan K Raine-Fenning N. Which follicles make the most anti-Mullerian hormone in humans? Mol Hum Reprod. (2013) 19:519–27. doi: 10.1093/molehr/gat024 23562944

[315] Hansen KR Hodnett GM Knowlton N Craig LB. Correlation of ovarian reserve tests with histologically determined primordial follicle number. Fertility Sterility. (2011) 95:170–5. doi: 10.1016/j.fertnstert.2010.04.006 20522327

[316] Nelson SM Davis SR Kalantaridou S Lumsden MA Panay N Anderson RA. Anti-Mü llerian hormone for the diagnosis and prediction of menopause: a systematic review. Hum Reprod Update. (2023) 29:327–46. doi: 10.1093/humupd/dmac045 PMC10152172 36651193

[317] de Vet A Laven JS de Jong FH Themmen AP Fauser BC. Antimüllerian hormone serum levels: a putative marker for ovarian aging. Fertility Sterility. (2002) 77:357–62. doi: 10.1016/s0015-0282(01)02993-4 11821097

[318] Meduri G Massin N Guibourdenche J Bachelot A Fiori O Kuttenn F. Serum anti-Mullerian hormone expression in women with premature ovarian failure. Hum Reprod. (2007) 22:117–23. doi: 10.1093/humrep/del346 16954410

[319] Sahmay S Usta TA Erel T Atakul N Aydogan B. Elevated LH levels draw a stronger distinction than AMH in premature ovarian insufficiency. Climacteric. (2014) 17:197–203. doi: 10.3109/13697137.2013.870149 24299186

[320] Li HWR Anderson RA Yeung WSB Ho PC Ng EHY. Evaluation of serum antimullerian hormone and inhibin B concentrations in the differential diagnosis of secondary oligoamenorrhea. Fertil Steril. (2011) 96:774–9. doi: 10.1016/j.fertnstert.2011.06.016 21737073

[321] Knauff EA Eijkemans MJ Lambalk CB ten Kate-Booij MJ Hoek A Beerendonk CC. Anti-müllerian hormone, inhibin B, and antral follicle count in young women with ovarian failure. J Clin Endocrinol Metab. (2009) 94:786–92. doi: 10.1210/jc.2008-1818 19066296

[322] Nelson SM Anderson RA. Prediction of premature ovarian insufficiency: foolish fallacy or feasible foresight? Climacteric. (2021) 24:438–43. doi: 10.1080/13697137.2020.1868426 33522318

[323] Jiao X Meng T Zhai Y Zhao L Luo W Liu P. Ovarian reserve markers in premature ovarian insufficiency: within different clinical stages and different etiologies. Front endocrinology. (2021) 12:601752. doi: 10.3389/fendo.2021.601752 PMC8015703 33815272

[324] Kallio S Aittomäki K Piltonen T Veijola R Liakka A Vaskivuo TE. Anti-Müllerian hormone as a predictor of follicular reserve in ovarian insufficiency: special emphasis on FSH-resistant ovaries. Hum Reprod (Oxford). (2012) 27:854–60. doi: 10.1093/humrep/der473 22258659

[325] Christin-Maitre S Givony M Albarel F Bachelot A Bidet M Blanc JV. Position statement on the diagnosis and management of premature/primary ovarian insufficiency (except Turner Syndrome). Ann Endocrinol. (2021) 82:555–71. doi: 10.1016/j.ando.2021.09.001 34508691

[326] Guzel Y Aba YA Yakin K Oktem O. Menstrual cycle characteristics of young females with occult primary ovarian insufficiency at initial diagnosis and one-year follow-up with serum amh level and antral follicle count. PLoS One. (2017) 12:e0188334. doi: 10.1371/journal.pone.0188334 29176793 PMC5703527

[327] de Kat AC Broekmans FJM Lambalk CB. Role of AMH in prediction of menopause. Front Endocrinol. (2021) 12:733731. doi: 10.3389/fendo.2021.733731 PMC8476919 34594304

[328] Gravholt CH Andersen NH Christin-Maitre S Davis SM Duijnhouwer A Gawlik A. Clinical practice guidelines for the care of girls and women with Turner syndrome. Eur J Endocrinol. (2024) 190:G53–G151. doi: 10.1093/ejendo/lvae050 38748847 PMC11759048

[329] Tassone F Iong KP Tong T Lo J Gane LW Berry-Kravis E. FMR1 CGG allele size and prevalence ascertained through newborn screening in the United States. Genome Med. (2012) 4:100. doi: 10.1186/gm401 23259642 PMC4064316

[330] Nolin SL Glicksman A Ding X Ersalesi N Brown WT Sherman SL. Fragile X analysis of 1112 prenatal samples from 1991 to 2010. Prenatal Diagnosis. (2011) 31:925–31. doi: 10.1002/pd.2815 21717484

[331] McGowan-Jordan J Hastings RJ Moore S. International System for Human Cytogenetic or Cytogenomic Nomenclature (ISCN): Some Thoughts. In: Liehr T. Cytogenet Genome Res. (2021) 161(5):225–6. doi: 10.1159/000516655 34407535

[332] Rossetti R Moleri S Guizzardi F Gentilini D Libera L Marozzi A. Targeted next-generation sequencing indicates a frequent oligogenic involvement in primary ovarian insufficiency onset. Front Endocrinol (Lausanne). (2021) 12:664645. doi: 10.3389/fendo.2021.664645 34803902 PMC8600266

[333] Zhang X Liu L Song F Song Y Dai H. Ages at menarche and menopause, and mortality among postmenopausal women. Maturitas. (2019) 130:50–6. doi: 10.1016/j.maturitas.2019.10.009 31706436

[334] Savonitto S Morici N Franco N Misuraca L Lenatti L Ferri LA. Age at menopause, extent of coronary artery disease and outcome among postmenopausal women with acute coronary syndromes. Int J Cardiol. (2018) 259:8–13. doi: 10.1016/j.ijcard.2018.02.065 29486998

[335] Muka T Oliver-Williams C Kunutsor S Laven JSE Fauser BCJM Chowdhury R. Association of age at onset of menopause and time since onset of menopause with cardiovascular outcomes, intermediate vascular traits, and all-cause mortality: A systematic review and meta-analysis. JAMA Cardiol. (2016) 1:767–76. doi: 10.1001/jamacardio.2016.2415 27627190

[336] Zhu D Chung HF Dobson AJ Pandeya N Giles GG Bruinsma F. Age at natural menopause and risk of incident cardiovascular disease: a pooled analysis of individual patient data. Lancet.Public Health. (2019) 4:e553–64. doi: 10.1016/S2468-2667(19)30155-0 PMC7118366 31588031

[337] Liu J Jin X Liu W Chen W Wang L Feng Z. The risk of long-term cardiometabolic disease in women with premature or early menopause: A systematic review and meta-analysis. Front Cardiovasc Med. (2023) 10. doi: 10.3389/fcvm.2023.1131251 PMC10072266 37025693

[338] Wu X Cai H Kallianpur A Li H Yang G Gao J. Impact of premature ovarian failure on mortality and morbidity among Chinese women. PLoS One. (2014) 9:e89597. doi: 10.1371/journal.pone.0089597 24603759 PMC3945971

[339] Christ JP Gunning MN Palla G Eijkemans MJC Lambalk CB Laven JSE. Estrogen deprivation and cardiovascular disease risk in primary ovarian insufficiency. Fertil Steril. (2018) 109:594,600.e1. doi: 10.1016/j.fertnstert.2017.11.035 29605405

[340] Langrish JP Mills NL Bath LE Warner P Webb DJ Kelnar CJ. Cardiovascular effects of physiological and standard sex steroid replacement regimens in premature ovarian failure. Hypertension (Dallas Tex.: 1979). (2009) 53:805–11. doi: 10.1161/HYPERTENSIONAHA.108.126516 19332659

[341] Popat VB Calis KA Vanderhoof VH Cizza G Reynolds JC Sebring N. Bone mineral density in estrogen-deficient young women. J Clin Endocrinol Metab. (2009) 94:2277–83. doi: 10.1210/jc.2008-1878 PMC2708959 19401379

[342] Bachelot A Rouxel A Massin N Dulon J Courtillot C Matuchansky C. Phenotyping and genetic studies of 357 consecutive patients presenting with premature ovarian failure. Eur J endocrinology. (2009) 161:179–87. doi: 10.1530/EJE-09-0231 19411303

[343] Samad N Nguyen HH Hashimura H Pasco J Kotowicz M Strauss BJ. Abnormal trabecular bone score, lower bone mineral density and lean mass in young women with premature ovarian insufficiency are prevented by oestrogen replacement. Front Endocrinol. (2022) 13. doi: 10.3389/fendo.2022.860853 PMC9162038 35663323

[344] Svejme O Ahlborg HG Nilsson JÅ Karlsson MK. Early menopause and risk of osteoporosis, fracture and mortality: a 34-year prospective observational study in 390 women. BJOG: an Int J obstetrics gynaecology. (2012) 119:810–6. doi: 10.1111/j.1471-0528.2012.03324.x 22531019

[345] Kiriakova V Cooray SD Yeganeh L Somarajah G Milat F Vincent AJ. Management of bone health in women with premature ovarian insufficiency: Systematic appraisal of clinical practice guidelines and algorithm development. Maturitas. (2019) 128:70–80. doi: 10.1016/j.maturitas.2019.07.021 31561827

[346] Nguyen HH Milat F Vincent AJ. New insights into the diagnosis and management of bone health in premature ovarian insufficiency. Climacteric: J Int Menopause Society. (2021) 24:481–90. doi: 10.1080/13697137.2021.1917539 33955314

[347] Almeida M Laurent MR Dubois V Claessens F O'Brien CA Bouillon R. Estrogens and androgens in skeletal physiology and pathophysiology. Physiol Rev. (2017) 97:135–87. doi: 10.1152/physrev.00033.2015 PMC5539371 27807202

[348] Nguyen HH Milat F Vincent A. Premature ovarian insufficiency in general practice: Meeting the needs of women. Aust Family physician. (2017) 46:360–6. 28609590

[349] Cardona Attard C Cameron-Pimblett A Puri D Elliot J Wilson JC Talaulikar VS. Fracture rate in women with oestrogen deficiency - Comparison of Turner syndrome and premature ovarian insufficiency. Clin Endocrinol (Oxf). (2019) 91:743–9. doi: 10.1111/cen.14110 31612507

[350] Meczekalski B Niwczyk O Bala G Szeliga A. Managing early onset osteoporosis: the impact of premature ovarian insufficiency on bone health. J Clin Med. (2023) 12:4042. doi: 10.3390/jcm12124042 37373735 PMC10299102

[351] Ferrari S Bianchi ML Eisman JA Foldes AJ Adami S Wahl DA. Osteoporosis in young adults: pathophysiology, diagnosis, and management. Osteoporosis international: J established as result cooperation between Eur Foundation Osteoporosis Natl Osteoporosis Foundation USA. (2012) 23:2735–48. doi: 10.1007/s00198-012-2030-x 22684497

[352] Słopień R. Mood disorders in women with premature ovarian insufficiency. Menopausal Review. (2018) 17:124–6. doi: 10.5114/pm.2018.78556 PMC6196781 30356982

[353] Xi D Chen B Hui Tao H Xu Y Chen G. The risk of depressive and anxiety symptoms in women with premature ovarian insufficiency: a systematic review and meta-analysis. Arch Women's Ment Health. (2023) 26:1–10. doi: 10.1007/s00737-022-01289-7 36705738 PMC9908676

[354] Rooney KL Domar AD. The relationship between stress and infertility. Dialogues Clin Neurosci. (2018) 20:41–7. doi: 10.31887/DCNS.2018.20.1/klrooney PMC6016043 29946210

[355] Moukhah S Ghorbani B Behboodi-Moghadam Z Zafardoust S. Perceptions and experiences of women with premature ovarian insufficiency about sexual health and reproductive health. BMC Womens Health. (2021) 21:54. doi: 10.1186/s12905-021-01197-5 33557799 PMC7869211

[356] Calik-Ksepka A Grymowicz M Rudnicka E Skórska J Machura P Pięta W. Signs and symptoms, evaluation, and management of genitourinary tract consequences of premature ovarian insufficiency. Menopausal Review. (2018) 17:131–4. doi: 10.5114/pm.2018.78558 PMC6196777 30357024

[357] Maciejewska-Jeske M Szeliga A Męczekalski B. Consequences of premature ovarian insufficiency on women’s sexual health. Menopausal Review. (2018) 17:127–30. doi: 10.5114/pm.2018.78557 PMC6196782 30357022

[358] Scavello I Maserol E Di Stasi V Vignozzi L. Sexual health in menopause. Medicina. (2019) 55:559. doi: 10.3390/medicina55090559 31480774 PMC6780739

[359] Vignozzi L Maseroli E. Hormones and sex behavior. Endocrinology. (2020), 1–28.31676839

[360] Graziottin A Basson R. Sexual dysfunction in women with premature menopause. Menopause. (2004) 11:766–77. doi: 10.1097/01.GME.0000139926.02689.A1 15543028

[361] Gosset A Jeanne Marie Claeys JM Huyghe E Tremollieres F. Sexual function and quality of life in women with idiopathic premature ovarian insufficiency. J Sexual Med. (2023) 20:626–32. doi: 10.1093/jsxmed/qdad006 36881744

[362] Blümel JE Chedraui P Vallejo MS Dextre M Elizalde A Escalante C. Genitourinary symptoms and sexual function in women with primary ovarian insufficiency. Climacteric: J Int Menopause Soc. (2024) 27:269–74. doi: 10.1080/13697137.2024.2306278 38308574

[363] Angelou K Grigoriadis T Diakosavvas M Zacharakis D Athanasiou S. The genitourinary syndrome of menopause: an overview of the recent data. Cureus. (2020) 12:e7586. doi: 10.7759/cureus.7586 32399320 PMC7212735

[364] Kanis JA McCloskey EV Johansson H Cooper C Rizzoli R Reginster JY. European guidance for the diagnosis and management of osteoporosis in postmenopausal women. Osteoporosis international: J established as result cooperation between Eur Foundation Osteoporosis Natl Osteoporosis Foundation USA. (2013) 24:23–57. doi: 10.1007/s00198-012-2074-y PMC3587294 23079689

[365] Bove R Secor E Chibnik LB Barnes LL Schneider JA Bennett DA. Age at surgical menopause influences cognitive decline and Alzheimer pathology in older women. Neurology. (2014) 82:222–9. doi: 10.1212/WNL.0000000000000033 PMC3902759 24336141

[366] Swee DS Javaid U Quinton R. Estrogen replacement in young hypogonadal women—Transferrable lessons from the literature related to the care of young women with premature ovarian failure and transgender women. Front Endocrinology. (2019) 10:685. doi: 10.3389/fendo.2019.00685 PMC6798086 31681164

[367] Yilmazer M Fenkci V Fenkci S Sonmezer M Aktepe O Altindis M. Hormone replacement therapy, C-reactive protein, and fibrinogen in healthy postmenopausal women. Maturitas. (2003) 46:245–53. doi: 10.1016/S0378-5122(03)00217-2 14625121

[368] Chu MC Cosper P Nakhuda GS Lobo RA. A comparison of oral and transdermal short-term estrogen therapy in postmenopausal women with metabolic syndrome. Menopause. (2006) 86:1669–75. doi: 10.1016/j.fertnstert.2006.04.043 17074346

[369] Crofton PM Evans N Bath LE Warner P Whitehead TJ Critchley HOD. Physiological versus standard sex steroid replacement in young women with premature ovarian failure: effects on bone mass acquisition and turnover. Clin Endocrinol (Oxf). (2010) 73:707–14. doi: 10.1111/j.1365-2265.2010.03868.x 20738314

[370] O'Donnell RL Warner P Lee RJ Walker J Bath LE Kelnar CJ. Physiological sex steroid replacement in premature ovarian failure: randomized crossover trial of effect on uterine volume, endometrial thickness and blood flow, compared with a standard regimen. Hum Reprod (Oxford). (2012) 27:1130–8. doi: 10.1093/humrep/des004 22343553

[371] Webber L Anderson RA Davies M Janse F Vermeulen N. HRT for women with premature ovarian insufficiency: a comprehensive review. Hum Reprod Open. (2017) 2017:hox007. doi: 10.1093/hropen/hox007 30895225 PMC6276684

[372] Cleemann L Holm K Kobbernagel H Kristensen B Skouby SO Jensen AK. Dosage of estradiol, bone and body composition in Turner syndrome: a 5-year randomized controlled clinical trial. Eur J endocrinology. (2017) 176:233–42. doi: 10.1530/EJE-16-0582 27881458

[373] Jayasena CN Devine K Barber K Comninos AN Conway GS Crown A. Society for endocrinology guideline for understanding, diagnosing and treating female hypogonadism. Clin Endocrinol. (2024). doi: 10.1111/cen.15097 39031660

[374] Burgos N Cintron D Latortue-Albino P Serrano V Rodriguez Gutierrez R Faubion S. Estrogen-based hormone therapy in women with primary ovarian insufficiency: a systematic review. Endocrine. (2017) 58:413–25. doi: 10.1007/s12020-017-1435-x PMC5765545 29039146

[375] Cintron D Rodriguez-Gutierrez R Serrano V Latortue-Albino P Erwin PJ Murad MH. Effect of estrogen replacement therapy on bone and cardiovascular outcomes in women with turner syndrome: a systematic review and meta-analysis. Endocrine. (2017) 55:366–75. doi: 10.1007/s12020-016-1046-y 27473099

[376] Popat VB Calis KA Kalantaridou SN Vanderhoof VH Koziol D Troendle JF. Bone mineral density in young women with primary ovarian insufficiency: results of a three-year randomized controlled trial of physiological transdermal estradiol and testosterone replacement. J Clin Endocrinol Metab. (2014) 99:3418–26. doi: 10.1210/jc.2013-4145 PMC4154086 24905063

[377] Cartwright B Robinson J Seed PT Fogelman I Rymer J. Hormone replacement therapy versus the combined oral contraceptive pill in premature ovarian failure: A randomized controlled trial of the effects on bone mineral density. J Clin Endocrinol Metab. (2016) 101:3497–505. doi: 10.1210/jc.2015-4063 27340881

[378] Zaiem F Alahdab F Al Nofal A Murad MH Javed A. Oral versus transdermal estrogen in turner syndrome: A systematic review and meta-analysis. Endocrine Pract. (2017) 23:408–21. doi: 10.4158/EP161622.OR 28095041

[379] Nabhan ZM Dimeglio LA Qi R Perkins SM Eugster EA. Conjugated oral versus transdermal estrogen replacement in girls with turner syndrome: A pilot comparative study. J Clin Endocrinol Metab. (2009) 94:2009–14. doi: 10.1210/jc.2008-2123 19318455

[380] Casanova G Spritzer PM. Effects of micronized progesterone added to non-oral estradiol on lipids and cardiovascular risk factors in early postmenopause: a clinical trial. Lipids Health Dis. (2012) 11:133. doi: 10.1186/1476-511X-11-133 23046709 PMC3508911

[381] Stute P Neulen J Wildt L. The impact of micronized progesterone on the endometrium: a systematic review. Climacteric: J Int Menopause Soc. (2016) 19:316–28. doi: 10.1080/13697137.2016.1187123 27277331

[382] Furness S Roberts H Marjoribanks J Lethaby A. Hormone therapy in postmenopausal women and risk of endometrial hyperplasia. Cochrane Database systematic Rev. (2012) 2012:CD000402. doi: 10.1002/14651858.CD000402.pub4 PMC7039145 22895916

[383] Collaborative Group on Hormonal Factors in Breast Cancer. Type and timing of menopausal hormone therapy and breast cancer risk: individual participant meta-analysis of the worldwide epidemiological evidence. Lancet. (2019) 394:1159–68. doi: 10.1016/S0140-6736(19)31709-X PMC6891893 31474332

[384] Sergison JE Maldonado LY Gao X Hubacher D. Levonorgestrel intrauterine system associated amenorrhea: a systematic review and metaanalysis. Obstet Gynecol. (2019) 220:440,448.e8. doi: 10.1016/j.ajog.2018.12.008 PMC6512461 30527945

[385] Davis SR Taylor S Hemachandra C Magraith K Ebeling PR Jane F. The 2023 practitioner’s toolkit for managing menopause. Climacteric. (2023) 26:517–36. doi: 10.1080/13697137.2023.2258783 37902335

[386] Gemzell-Danielsson K Cagnacci A Chabbert-Buffet N Douxfils J Foidart JM Kubba A. A novel estetrol-containing combined oral contraceptive: European expert panel review. Eur J contraception Reprod Health care: Off J Eur Soc Contraception. (2022) 27:373–83. doi: 10.1080/13625187.2022.2093850 35862627

[387] Piedade KC Spencer H Persani L Nelson LM. Optimizing fertility in primary ovarian insufficiency: case report and literature review. Front Genet. (2021) 12:676262. doi: 10.3389/fgene.2021.676262 34249096 PMC8261244

[388] Nordenström A Ahmed SF van den Akker E Blair J Bonomi M Brachet C. Pubertal induction and transition to adult sex hormone replacement in patients with congenital pituitary or gonadal reproductive hormone deficiency: an Endo-ERN clinical practice guideline. Eur J Endocrinol. (2022) 186:G9–G49. doi: 10.1530/EJE-22-0073 35353710 PMC9066594

[389] Federici S Persani L Bonomi M. Approccio diagnostico alla paziente con insufficienza ovarica primaria. L’Endocrinologo. (2022) 23:59–62. doi: 10.1007/s40619-022-01109-1

[390] Burt E Davies MC Yasmin E Cameron-Pimblett A Mavrelos D Talaulikar V. Reduced uterine volume after induction of puberty in women with hypogonadism. Clin Endocrinol (Oxf). (2019) 91:798–804. doi: 10.1111/cen.14092 31487390

[391] Davenport ML. Approach to the patient with Turner syndrome. J Clin Endocrinol Metab. (2010) 95:1487–95. doi: 10.1210/jc.2009-0926 20375216

[392] Gawlik AM Hankus M Szeliga K Antosz A Gawlik T Soltysik K. Late-onset puberty induction by transdermal estrogen in turner syndrome girls—A longitudinal study. Front Endocrinol (Lausanne). (2018) 9:23. doi: 10.3389/fendo.2018.00023 29472893 PMC5810248

[393] Hamoda H. The British Menopause Society and Women's Health Concern recommendations on the management of women with premature ovarian insufficiency. Post Reprod Health. (2017) 23:22–35. doi: 10.1177/2053369117699358 28381102

[394] Deli T Orosz M Jakab A. Hormone replacement therapy in cancer survivors – review of the literature. Pathol Oncol Res. (2020) 26:63. doi: 10.1007/s12253-018-00569-x 30617760 PMC7109141

[395] Benetti-Pinto C Soares PM Magna LA Petta CA Santos CCD. Breast density in women with premature ovarian failure using hormone therapy. Gynecological endocrinology: Off J Int Soc Gynecological Endocrinology. (2008) 24:40–3. doi: 10.1080/09637480701690543 18224543

[396] Wu JY Chen WG Chen XS Huang O He JR Zhu L. Outcomes of adjuvant endocrine therapy and hormone receptor status change following neoadjuvant chemotherapy in breast cancer patients. Int J Biol Markers. (2014) 29:380–6. doi: 10.5301/jbm.5000113 25385240

[397] Bosze P Toth A Torok M. Hormone replacement and the risk of breast cancer in turner's syndrome. New Engl J Med. (2006) 355:2599–600. doi: 10.1056/NEJMc062795 17167149

[398] Manchanda R Gaba F Talaulikar V Pundir J Gessler S Davies M. Risk-reducing salpingo-oophorectomy and the use of hormone replacement therapy below the age of natural menopause: scientific impact paper no. 66 october 2021: scientific impact paper no. 66. BJOG: an Int J obstetrics gynaecology. (2022) 129:e16–34. doi: 10.1111/1471-0528.16896 PMC7614764 34672090

[399] Bernstein-Molho R Friedman E Evron E. Controversies and open questions in management of cancer-free carriers of germline pathogenic variants in BRCA1/BRCA2. Cancers. (2022) 14:4592. doi: 10.3390/cancers14194592 36230512 PMC9559251

[400] Hickey M Basu P Sassarini J Stegmann ME Weiderpass E Nakawala Chilowa K. Managing menopause after cancer. Lancet (London England). (2024) 403:984–96. doi: 10.1016/S0140-6736(23)02802-7 38458217

[401] Taboada M Santen R Lima J Hossain J Singh R Klein KO. Pharmacokinetics and pharmacodynamics of oral and transdermal 17β Estradiol in girls with turner syndrome. J Clin Endocrinol Metab. (2011) 96:3502–10. doi: 10.1210/jc.2011-1449 PMC3205885 21880799

[402] Pacello PCC Yela DA Rabelo S Giraldo PC Benetti-Pinto C. Dyspareunia and lubrication in premature ovarian failure using hormonal therapy and vaginal health. Climacteric: J Int Menopause Society. (2014) 17:342–7. doi: 10.3109/13697137.2013.860116 24188246

[403] Janse F Tanahatoe SJ Eijkemans MJC Fauser BCJM. Testosterone concentrations, using different assays, in different types of ovarian insufficiency: a systematic review and meta-analysis. Hum Reprod Update. (2012) 18:405–19. doi: 10.1093/humupd/dms013 22525963

[404] Davis SR Baber R Panay N Bitzer J Perez SC Islam RM. Global consensus position statement on the use of testosterone therapy for women. Maturitas. (2019) 128:89–93. doi: 10.1016/j.maturitas.2019.07.001 31484631

[405] Sassarini J Lumsden MA. Non-hormonal management of vasomotor symptoms. Climacteric. (2013) 16:31–6. doi: 10.3109/13697137.2013.805525 23848489

[406] Pinkerton JV Santen RJ. Managing vasomotor symptoms in women after cancer. Climacteric. (2019) 22:544–52. doi: 10.1080/13697137.2019.1600501 31081391

[407] Guthrie KA Lacroix AZ Ensrud KE Joffe H Newton KM Reed SD. Pooled analysis of six pharmacologic and nonpharmacologic interventions for vasomotor symptoms. Obstet Gynecol. (2015) 126:413–22. doi: 10.1097/AOG.0000000000000927 PMC4526122 26241433

[408] Neal-Perry G Cano A Lederman S Nappi RE Santoro N Wolfman W. Safety of fezolinetant for vasomotor symptoms associated with menopause: A randomized controlled trial. Obstetrics gynecology (New York. 1953). (2023) 141:737–47. doi: 10.1097/AOG.0000000000005114 PMC10026946 36897180

[409] Patel B S. Dhillo W. Menopause review: Emerging treatments for menopausal symptoms. Best Pract Res Clin obstetrics gynaecology. (2022) 81:134–44. doi: 10.1016/j.bpobgyn.2021.10.010 34965909

[410] ESHRE Guideline Group on Female Fertility Preservation Anderson RA Amant F Braat D D'Angelo A Chuva de Sousa Lopes SM. ESHRE guideline: female fertility preservation. Hum Reprod Open. (2020) 2020:hoaa052. doi: 10.1093/hropen/hoaa052 33225079 PMC7666361

[411] Walker Z Lanes A Ginsburg E. Oocyte cryopreservation review: outcomes of medical oocyte cryopreservation and planned oocyte cryopreservation. Reprod Biol Endocrinol. (2022) 20:10–0. doi: 10.1186/s12958-021-00884-0 PMC8740039 34996479

[412] Oktay K Bedoschi G Berkowitz K Bronson R Kashani B McGovern P. Fertility preservation in women with turner syndrome: A comprehensive review and practical guidelines. J Pediatr Adolesc Gynecol. (2016) 29:409–16. doi: 10.1016/j.jpag.2015.10.011 PMC5015771 26485320

[413] Gayete-Lafuente S Turan V Oktay KH. Oocyte cryopreservation with *in vitro* maturation for fertility preservation in girls at risk for ovarian insufficiency. J Assist Reprod Genet. (2023) 40:2777–85. doi: 10.1007/s10815-023-02932-7 PMC10656385 37715873

[414] Pfeifer S Goldberg J Lobo R Thomas M Pisarska M Widra E. Fertility preservation in patients undergoing gonadotoxic therapy or gonadectomy: a committee opinion. Fertil Steril. (2019) 112:1022–33. doi: 10.1016/j.fertnstert.2019.09.013 31843073

[415] Rosario R Anderson RA. Novel approaches to fertility restoration in women with premature ovarian insufficiency. Climacteric: J Int Menopause Society. (2021) 24:491–7. doi: 10.1080/13697137.2020.1856806 33427510

[416] Grynberg M Bidet M Benard J Poulain M Sonigo C Cédrin-Durnerin I. Fertility preservation in Turner syndrome. Fertil Steril. (2016) 105:13–9. doi: 10.1016/j.fertnstert.2015.11.042 26677790

[417] Huang JYJ Tulandi T Holzer H Lau NM MacDonald S Tan SL. Cryopreservation of ovarian tissue and *in vitro* matured oocytes in a female with mosaic Turner syndrome: Case Report. Hum Reprod. (2008) 23:336–9. doi: 10.1093/humrep/dem307 18056118

[418] Zhai J Yao G Dong F Bu Z Cheng Y Sato Y. *In vitro* activation of follicles and fresh tissue auto-transplantation in primary ovarian insufficiency patients. J Clin Endocrinol Metab. (2016) 101:4405–12. doi: 10.1210/jc.2016-1589 PMC5095246 27571179

[419] Prasath EB Chan MLH Wong WHW Lim CJW Tharmalingam MD Hendricks M. First pregnancy and live birth resulting from cryopreserved embryos obtained from *in vitro* matured oocytes after oophorectomy in an ovarian cancer patient. Hum Reprod. (2014) 29:276–8. doi: 10.1093/humrep/det420 24327539

[420] Uzelac PS Delaney AA Christensen GL Bohler HCL Nakajima ST. Live birth following *in vitro* maturation of oocytes retrieved from extracorporeal ovarian tissue aspiration and embryo cryopreservation for 5 years. Fertil Steril. (2015) 104:1258–60. doi: 10.1016/j.fertnstert.2015.07.1148 26297647

[421] Kawamura K Cheng Y Suzuki N Deguchi M Sato Y Takae S. Hippo signaling disruption and Akt stimulation of ovarian follicles for infertility treatment. Proc Natl Acad Sci. (2013) 110:17474–9. doi: 10.1073/pnas.1312830110 PMC3808580 24082083

[422] Suzuki N Yoshioka N Takae S Sugishita Y Tamura M Hashimoto S. Successful fertility preservation following ovarian tissue vitrification in patients with primary ovarian insufficiency. Hum Reprod. (2015) 30:608–15. doi: 10.1093/humrep/deu353 25567618

[423] Kim HK Kim TJ. Current status and future prospects of stem cell therapy for infertile patients with premature ovarian insufficiency. Biomolecules. (2024) 14:242. doi: 10.3390/biom14020242 38397479 PMC10887045

[424] Takahashi A Yousif A Hong L Chefetz III. Premature ovarian insufficiency: pathogenesis and therapeutic potential of mesenchymal stem cell. J Mol Med. (2021) 99:637–50. doi: 10.1007/s00109-021-02055-5 33641066

[425] Cakiroglu Y Saltik A Yuceturk A Karaosmanoglu O Kopuk SY Scott RT. Effects of intraovarian injection of autologous platelet rich plasma on ovarian reserve and IVF outcome parameters in women with primary ovarian insufficiency. Aging (Albany NY). (2020) 12:10211. doi: 10.18632/aging.v12i11 32507764 PMC7346073

[426] Moustaki M Kontogeorgi A Tsangkalova G Tzoupis H Makrigiannakis A Vryonidou A. Biological therapies for premature ovarian insufficiency: what is the evidence? Front Reprod Health. (2023) 5. doi: 10.3389/frph.2023.1194575 PMC10512839 37744287

[427] Pellicer N Cozzolino M Diaz-García C Galliano D Cobo A Pellicer A. Ovarian rescue in women with premature ovarian insufficiency: facts and fiction. Reprod biomedicine online. (2023) 46:543–65. doi: 10.1016/j.rbmo.2022.12.011 36710157

[428] Hikabe O Hamazaki N Nagamatsu G Obata Y Hirao Y Hamada N. Reconstitution *in vitro* of the entire cycle of the mouse female germ line. Nature. (2016) 539:299–303. doi: 10.1038/nature20104 27750280

[429] Yoshino T Suzuki T Nagamatsu G Yabukami H Ikegaya M Kishima M. Generation of ovarian follicles from mouse pluripotent stem cells. Science. (2021) 373:eabe0237. doi: 10.1126/science.abe0237 34437124

[430] Yamashiro C Sasaki K Yabuta Y Kojima Y Nakamura T Okamoto I. Generation of human oogonia from induced pluripotent stem cells *in vitro* . Science. (2018) 362:356–60. doi: 10.1126/science.aat1674 30237246

[431] Chen D Gell JJ Tao Y Sosa E Clark AT. Modeling human infertility with pluripotent stem cells. Stem Cell Res. (2017) 21:187–92. doi: 10.1016/j.scr.2017.04.005 PMC5498156 28431857

[432] Saitou M Hayashi K. Mammalian *in vitro* gametogenesis. Science. (2021) 374:. doi: 10.1126/science.aaz6830 34591639

[433] Nelson LM Spencer H Hijane K Thinuan P Nelson CW Vincent AJ. My 28 Days - a global digital women's health initiative for evaluation and management of secondary amenorrhea: case report and literature review. Front Endocrinol (Lausanne). (2023) 14:1227253. doi: 10.3389/fendo.2023.1227253 37772077 PMC10523024

